# Herbarium specimens reveal a cryptic invasion of polyploid *Centaurea stoebe* in Europe

**DOI:** 10.1111/nph.20212

**Published:** 2024-10-23

**Authors:** Christoph Rosche, Olivier Broennimann, Andriy Novikov, Viera Mrázová, Ganna V. Boiko, Jiří Danihelka, Michael T. Gastner, Antoine Guisan, Kevin Kožić, Marcus Lehnert, Heinz Müller‐Schärer, Dávid U. Nagy, Ruben Remelgado, Michał Ronikier, Julian A. Selke, Natalia M. Shiyan, Tomasz Suchan, Arpad E. Thoma, Pavel Zdvořák, Patrik Mráz

**Affiliations:** ^1^ Institute of Geobotany Martin Luther University Halle‐Wittenberg Halle 06108 Germany; ^2^ German Centre for Integrative Biodiversity Research (iDiv) Halle‐Jena‐Leipzig Leipzig 04103 Germany; ^3^ Department of Ecology and Evolution University of Lausanne Lausanne 1015 Switzerland; ^4^ Institute of Earth Surface Dynamics University of Lausanne Lausanne 1015 Switzerland; ^5^ State Museum of Natural History, National Academy of Sciences of Ukraine Lviv 79008 Ukraine; ^6^ Department of Botany Charles University Prague 12801 Czech Republic; ^7^ M.G. Kholodny Institute of Botany, National Academy of Sciences of Ukraine Kyiv 01601 Ukraine; ^8^ Department of Botany and Zoology Masaryk University Brno 60200 Czech Republic; ^9^ Institute of Botany, Czech Academy of Sciences Průhonice 25243 Czech Republic; ^10^ Information and Communication Technology Cluster Singapore Institute of Technology Singapore 828608 Singapore; ^11^ Department of Biology University of Fribourg Fribourg 1700 Switzerland; ^12^ College of Resources & Environment Huazhong Agricultural University Wuhan 430070 China; ^13^ W. Szafer Institute of Botany, Polish Academy of Sciences Kraków 31‐512 Poland; ^14^ Faculty of Informatics and Data Science University of Regensburg Regensburg 93040 Germany; ^15^ Herbarium collections Charles University Prague 12801 Czech Republic

**Keywords:** *Centaurea stoebe* (spotted knapweed), climatic niche, colonization ability, cryptic invasion, herbarium specimens, polyploidy, range expansion, ruderal habitats

## Abstract

- Numerous plant species are expanding their native ranges due to anthropogenic environmental change. Because cytotypes of polyploid complexes often show similar morphologies, there may be unnoticed range expansions (i.e. cryptic invasions) of one cytotype into regions where only the other cytotype is native.
- We critically revised herbarium specimens of diploid and tetraploid *Centaurea stoebe*, *collected across* Europe between 1790 and 2023. Based on their distribution in natural and relict habitats and phylogeographic data, we estimated the native ranges of both cytotypes.
- Diploids are native across their entire European range, whereas tetraploids are native only to South‐Eastern Europe and have recently expanded their range toward Central Europe. The proportion of tetraploids has exponentially increased over time in their expanded but not in their native range. This cryptic invasion predominantly occurred in ruderal habitats and enlarged the climatic niche of tetraploids toward a more oceanic climate.
- We conclude that spatio‐temporally explicit assessments of range shifts, habitat preferences and niche evolution can improve our understanding of cryptic invasions. We also emphasize the value of herbarium specimens for accurate estimation of species´ native ranges, with fundamental implications for the design of research studies and the assessment of biodiversity trends.

## Introduction

Many invasive plants do not only spread in terms of ‘classical’, transcontinental invasions but may simultaneously expand their native range within continents (van Kleunen *et al*., 2015; Essl *et al*., 2019; Lustenhouwer *et al*., 2024). In Europe, for example, the number of naturalized, non‐native plants with intracontinental origin exceeds those with transcontinental origin (Zhang *et al*., 2023). However, our understanding of such gradual range expansions is limited because the precise borders of the ‘true’ native ranges of species are usually unknown or speculative (Lustenhouwer & Parker, 2022; Zhang *et al*., 2023). This lack of information may be particularly common in polyploid complexes as closely related diploid and polyploid cytotypes often show similar morphologies. Consequently, range expansions of a cytotype can go unnoticed in areas where the other cytotype is already present (Rüegg *et al*., 2017; Mezhzherin *et al*., 2022; Kúr *et al*., 2023). These expansions can be regarded as ‘cryptic invasions’ (*sensu* Novak, 2011), a phenomenon believed to be much more widespread and ecologically significant than currently recognized (Bickford *et al*., 2007; Morais & Reichard, 2018).

There are several examples among polyploid complexes where the polyploid cytotype is a more successful invader than its diploid counterpart (reviewed by te Beest *et al*., 2012). Moreover, polyploids are presumed to be more successful in ruderal habitats than diploids (van Drunen & Johnson, 2022). These patterns indicate that polyploids, relative to diploids, might benefit from the steadily increasing anthropogenic activity under global change (Sessa, 2019; van de Peer *et al*., 2021). Analyzing shifts in cytotype frequencies within sympatric ranges of polyploid complexes, such as in the case of cryptic invasions, may provide evidence of the superior colonization abilities of polyploids. However, there is no empirical quantification of a cryptic invasion by a polyploid plant expanding into the range of its diploid relative.

Herbarium collections provide invaluable resources for tracking such spatio‐temporal dynamics of species occurrences (Delisle *et al*., 2003; Lang *et al*., 2019; Rosche *et al*., 2022). Furthermore, herbarium labels often contain information in which habitat type the specimen was collected. This is important because during range expansions, species are much more likely to be found in ruderal rather than in natural habitats (Theoharides & Dukes, 2007; Dainese *et al*., 2017; Essl *et al*., 2019). Thus, studying spatio‐temporal shifts in the proportion of polyploid vs diploid cytotypes, while differentiating between ruderal and natural habitats, may help uncover cryptic invasions in polyploid complexes.

Further applications of herbarium records in invasion research include reconstructing temporal peaks of range expansions (Delisle *et al*., 2003) and assessing how climatic niche breadths change across space and time (Broennimann *et al*., 2014). Given that anthropogenic factors can have a comparable or even stronger effect than macroclimate on species distributions (McKeon *et al*., 2023), our understanding of the key determinants of range expansions may be improved by integrating spatio‐temporally explicit data on human activities and climate (Skokanová *et al*., 2023). The emerging spatio‐temporally explicit databases on human settlements (Schiavina *et al*., 2023) and transport systems (Garcia‐López *et al*., 2022) offer hitherto unexplored tools to disentangle the relative roles of climate, space, dispersal corridors and urbanization in range expansions. Regarding polyploid vs diploid distribution patterns, such integrative approaches could significantly contribute to understanding the circumstances under which cytotype shifts have occurred in the past and may continue doing so in the future.

Here we study the cryptic invasion of tetraploid *Centaurea stoebe* L. (spotted knapweed; Asteraceae). Diploids of this species are suggested to be native to large parts of Europe, including Central Europe (Ochsmann, 2000). As native, we define regions where taxa have naturally occurred before the strong anthropogenic activities of the last centuries have shifted the distribution of many species (Essl *et al*., 2019). By contrast, tetraploids are supposed to be only native to South‐Eastern Europe from where they might have recently expanded their range toward Central Europe (Ochsmann, 2000; Mráz *et al*., 2012a; Rosche *et al*., 2016). Tetraploid *C. stoebe* is among the most successful plant invaders of North America, whereas diploids have never been recorded outside their native range (Mráz *et al*., 2011). This distribution pattern has made *C. stoebe* a prominent model to study the benefits of polyploidy in invasion success (te Beest *et al*., 2012). However, while the North American invasion history of tetraploid *C. stoebe* is well‐documented (Broennimann *et al*., 2014), the cryptic invasion across Central Europe has remained speculative. The need to address this knowledge gap became evident when we reviewed 52 recent papers that used ‘native’ tetraploid populations from Europe to compare them with non‐native tetraploid populations from North America (Supporting Information Table S1). Intriguingly, all the reviewed studies involved Central European tetraploid populations (which might be *not* native) and treated them as native in their comparisons. This underscores the necessity of accurately delineating native and expanded ranges for eco‐evolutionary studies.

To unravel the cryptic invasion of tetraploid *C. stoebe*, we analyzed spatio‐temporally explicit occurrence data, including habitat type information, from herbarium specimens and cytogeographic data (i.e. chromosome counts or flow cytometry analyses). We addressed the following principal objectives:

1. Fostering our understanding of how to distinguish between native and expanded species ranges: We examined the geographical ranges of diploid and tetraploid *C. stoebe* and employed a conceptual framework predominantly focusing on the distribution in natural and relict habitats to delineate the native and expanded ranges of both cytotypes. As a proof of concept, we investigated how the proportion of tetraploids, relative to diploids, has changed over time in the native and expanded ranges. More specifically, we asked whether these temporal dynamics have been similarly or differently pronounced in ruderal and natural habitats in either range.
2. Facilitating our understanding of the characteristics and determinants of the cryptic invasion of tetraploids: We investigated how the geographical range size and the climatic niche breadth of tetraploids evolved over time in ruderal and natural habitats of their expanded range. Finally, we examined the relative importance of climatic conditions vs anthropogenic factors, such as dispersal corridors and urbanization, in both the initial spread and the current occurrence of tetraploids.

## Materials and Methods

### Total dataset

We critically revised 13 078 *Centaurea stoebe* L. herbarium specimens from 167 herbaria (Table S2; Fig. S1). For cytotype determination, we applied the discrimination key from Mráz *et al*. (2011), which primarily relies on distinguishing the cytotypes based on the combination of the following traits: shape and size of the capitula, shape and size of the involucral bracts and their color and the presence/absence of accessory rosettes (Notes S1). This step was crucial because diploid and tetraploid plants are morphologically similar, and the taxonomic acceptance of the two cytotypes is relatively recent (Mráz *et al*., 2011). Consequently, only 9.6% of the specimens were originally determined to the cytotype level on their specimen labels and even from those specimens, 18.2% were found to be misidentified.

We applied two molecular approaches to validate our cytotype determination: (1) We morphologically determined the cytotype of 463 individuals grown in a common garden at the University of Fribourg and subsequently verified our determination using flow cytometry (morphological determination accuracy: 98.3%). (2) We genotyped the ITS1 locus of 178 herbarium specimens after morphological determination of their cytotypes (morphological determination accuracy: 97.8%). We choose this subsample (3.5% of the total number of specimens) to represent comparable distributional ranges and collection dates across both cytotypes, including specimens from both the native and expanded ranges of tetraploid *C. stoebe*. Genotyping of the ITS1 locus unambiguously identifies the cytotype, as all tetraploid samples exhibit a unique ribotype B, which has never been found in diploid samples (Mráz *et al*., 2012a). Overall, our complementary validation approaches demonstrated that the morphology‐based determination was very reliable and consistent across the investigated spatial and temporal ranges of our herbarium collections. Details on the validation of our morphological cytotype determination are provided in the Notes S1.

From the 13 078 specimens, we removed duplicates (i.e. specimens collected simultaneously at the same site: 41.6%) and specimens that did not belong to the *C. stoebe* complex (1.5%). We additionally excluded specimens where determining the cytotype was not possible (10.7%) or information on locality or collection date was missing or unreadable (6.8%). We then added 668 cytogeographic records, including 562 published records from 38 publications (Table S3) and 106 new records (Table S4). The new records were collected for the present study and analyzed using flow cytometry following the protocol in Mráz *et al*. (2011). After the data cleaning and adding steps (see Fig. S2 for a flowchart), our total dataset included 5803 occurrences. This total dataset was used to explore the entire Eurasian range of the *C. stoebe* complex and to estimate which part of the ranges are native for both cytotypes.

### Estimation of the native and expanded ranges of tetraploids

To delineate the native and expanded ranges of tetraploids, we developed a conceptual framework combining three approaches commonly employed to unveil native range expansions and cryptic invasions (Morais & Reichard, 2018; Lustenhouwer & Parker, 2022): (1) revision of occurrence data, (2) phylogeographic analyses, that is analyses on contemporary spatial patterns of molecular variation, and (3) review of floristic publications. Among these three approaches, our delineation primarily, but not exclusively, relied on the revision of occurrence data. Below we present a brief overview of the methods. Further details on our estimation are provided in the Notes S2.

First, we defined habitat types in which tetraploids may have existed in the recent past (Essl *et al*., 2019), that is where tetraploids could have naturally occurred before anthropogenic activities completely transformed the European landscape. For the light‐demanding *C. stoebe*, this includes zonal steppes, canopy‐open forests on steep slopes or sandy sediments, and extrazonal, treeless habitats. The latter were hereafter referred to as ‘relict habitats’ and included rock outcrops and naturally treeless habitats at high altitudes. These relict habitats were particularly interesting to us because *C. stoebe* is a predominantly barochorous species, which limits its uphill dispersal (Mráz *et al*., 2012b). Given this limited uphill dispersal, the colonization of relict sites should require the presence of nearby source populations over an extended period. After defining the historical habitat requirements, we employed our total dataset to identify geographic patterns in the habitat‐specific distribution of tetraploids. We considered those geographic regions as native range where tetraploids regularly occur at natural or relict habitats (regardless of whether they also occur in anthropogenic sites within the same region). By contrast, regions were considered as a part of the expanded range where tetraploids did not occur in natural or relict habitats. We did this habitat‐focused assessment independently of collection time. To ensure that the absence of tetraploid records at relict sites was not a signature of insufficient sampling activity in distinct regions, we took into account whether diploids occurred in relict habitats there. Accounting for the occurrence of a reference taxon is a recommended strategy for addressing spatial sampling bias in herbarium studies (Lang *et al*., 2019). Diploids are a suitable reference because they show large‐scale presence in relict habitats across their entire sympatric European range (see Notes S2) and they share comparable ecological niches with tetraploids (Mráz *et al*., 2011; Rosche *et al*., 2018). In regions that were close to the estimated border but were not well represented by neither diploid nor tetraploid herbarium specimens, we conducted extensive field surveys combined with flow cytometric analyses (Tables S3, S4) to aid decision‐making in these critical regions.

Second, we investigated spatial patterns of molecular variation, using data from five published and three unpublished phylogeographic datasets (Notes S2). Analyzing spatial patterns of contemporary genetic diversity is a well‐established method for identifying signatures of recent or historic cryptic invasions (reviewed by Morais & Reichard, 2018). The geographical distribution of genetic diversity within tetraploids, especially with a view on rare alleles, indicates that Central Europe has been colonized rather recently. The distribution of closely related taxa that share ribotypes with allotetraploid *C. stoebe* suggest the Balkans and adjacent regions of Romania and southern Ukraine to be the evolutionary cradle of tetraploid *C. stoebe*. In particular, the second parental species likely existed near the Black Sea before going extinct. Overall, the phylogeographic patterns supported the range delineation estimated from occurrence data. In addition, the phylogeographic data facilitated our assessments in regions where tetraploids are native but currently have sparse distributions (e.g. Ukrainian steppes, which have undergone extensive conversion to agricultural land).

Thirdly, we conducted an extensive literature survey of floristic publications. We found 27 local reports suggesting that specific tetraploid *C. stoebe* populations are not native to their respective location. For all 27 reports, our assessment confirmed their suggestions showing that our assessments were largely congruent with expert knowledge of local botanists (Notes S2).

Finally, we applied the same three approaches and criteria to assess the native range of diploids. In contrast to our findings for tetraploids, we found no credible evidence of a recent range expansion for diploids (for details, see paragraph 3.12 within the Notes S2).

We used ArcGIS 9.2 (ESRI, Redlands, CA, USA) to delineate the estimated boundary of the native range of tetraploids along the geographical barriers that we identified as separating the native and expanded ranges, such as rivers and mountains. Occurrence data within this boundary were classified as native, whereas those lying outside were designated as being part of the expanded range. For occurrences within a 50‐km buffer around the boundary (arbitrarily chosen threshold), we carried out individual verifications to confirm their range affiliation. These individual verifications were conducted to avoid incorrect assignments potentially arising from imprecise border delineation or inaccuracies in georeferencing.

### Focal dataset and study range

Our assessment identified two regions where tetraploids occur beyond their native range: (1) northwest of the native range encompassing Central, Western and Northern Europe and (2) east of the native range, specifically south and east of the Don River. For all analyses described below, we used our ‘focal dataset’ focusing on our ‘study range’, that is the native range and the expansion toward Central, Western and Northern Europe (Fig. S2). Records east of the native range were not included because the estimated delineation of the native range may be less precise in European Russia due to lower sampling efforts. We had 4417 records from Central, Western and Northern Europe but only 330 from the expanded range in European Russia despite both ranges having similar sizes. The lower sampling density in European Russia can be attributed to the relatively sparse distribution of herbaria (Fig. S1).

### Ruderal vs natural habitat type assignment

Habitat information was extracted from the labels of herbarium specimens or field notes from cytogeographic collections. With the available information, we classified the habitat types according to the European classification system of habitats (EUNIS). For 28.8% of our records, we could not retrieve sufficient habitat information for classification. Following the approach outlined in Broennimann *et al*. (2014), habitat types classified as diluvial sediments (EUNIS category C), natural and seminatural grasslands (E), and natural rock outcrops (H) were classified as ‘natural’ habitats. By contrast, agricultural habitats (I) and transport networks, extractive sites, urban and industrial habitats (J) were classified as ‘ruderal’ habitats.

### Data analyses

To predict the probability that a *C. stoebe* population within a given spatial, temporal and environmental context is tetraploid, we fitted generalized additive logistic models (GAMs) using the package mgcv 1.8‐41 (Wood, 2011) in R 4.3.3 (R Core Team, 2024). We modeled the spatio‐temporal dynamics using individual, binomial occurrence data (tetraploid = ‘1’ vs diploid = ‘0’), that is predicting the proportion of tetraploid relative to all *C. stoebe* records. This binomial response accommodates the inherent inconsistencies of herbarium collection efforts across space and time (Lang *et al*., 2019). Specifically, spatio‐temporal dynamics in species occurrences should not be predicted by the absolute numbers of specimens collected but rather in relation to a reference species, and this is particularly important in research on range expansions (Delisle *et al*., 2003). Diploid *C. stoebe* is a suitable reference taxon because diploids show an even distribution across the study range over the last two centuries (see Notes S2). At the same time, using diploids as a reference directly addresses the conundrum that polyploid plants become more frequent than diploids in some, but not all, environmental contexts in the Anthropocene (te Beest *et al*., 2012; van Drunen & Johnson, 2022).

We first predicted the proportion of tetraploids as a function of time in the native vs expanded ranges using a logistic thin plate spline‐based smoother function on the predictors ‘year by range’ + ‘range’. Given that range expansions primarily occur in ruderal habitats (Theoharides & Dukes, 2007), we then compared the temporal patterns in the proportion of tetraploids between ruderal and natural habitats, which we did separately for the native and expanded ranges. These two GAMs thus included the predictors ‘year by habitat type’ + ‘habitat type’. To account for spatial autocorrelation, all GAMs included a spline‐on‐the‐sphere smoothing term based on latitude and longitude (see Table S5 for full model structures). Concurvity among year, latitude and longitude was always below 0.15 indicating very low multicollinearity (Wood *et al*., 2016). To identify significant predictors, model performances were compared based on the Akaike information criterion (AIC), using ΔAIC ≤ −2 as a threshold for significance.

We then assessed the cumulative number of records over time, constructing so‐called invasion curves (Delisle *et al*., 2003) of colonized 10 km × 10 km pixels. To estimate realized climatic niches of both cytotypes, we performed a PCA on the 19 standard Bioclim variables from chelsa 2.1 (Karger *et al*., 2017). The first axis of this PCA was negatively correlated with several precipitation variables, thus representing a gradient from high to low precipitation (Fig. S3). The second axis correlated positively with several temperature variables and negatively with seasonality in temperature, thus representing a temperature gradient from continental toward warmer climate. To estimate climatic niche similarity between diploids and tetraploids across the study range, we calculated dynamic range boxes, which quantify size and overlap of n‐dimensional hyper volumes (Junker *et al*., 2016; Kuppler *et al*., 2017). We used the principal components of the 19 Bioclim variables to calculate the niche breadths of both cytotypes. Box sizes were calculated as mean side lengths of the PC‐axes over 201 quantiles. We similarly compared the overlap between the climatic niches of tetraploids in their native and expanded ranges (Lucas *et al*., 2024).

The site scores of the PCA were used to perform niche‐over‐time plots according to Broennimann *et al*. (2014). To ensure conservative niche limits, we removed occurrence data out of the 10 and 90 percentiles. These outliers may reflect artifacts from the modeled climatic data or sites with unsuitable macroclimate that are significantly influenced by favorable microclimatic conditions (Broennimann *et al*., 2014).

To spatially reconstruct the initial spread routes of the cryptic invasion of tetraploids, we predicted the most parsimonious dispersal routes using a minimum cost arborescence algorithm (Hordijk & Broennimann, 2012). To study relationships between the dispersal routes and climatic dissimilarity, we plotted the dispersal routes on a map showing the niche dissimilarity between the native and the expanded range of tetraploids. The spatial dissimilarity patterns were quantified by a multivariate environmental similarity surface (MESS) analysis, using the R‐package dismo 1.3‐14 (Hijmans *et al*., 2023). The native climatic niche of tetraploids was estimated from climatic data of all grid cells occupied by tetraploids in their native range. We then compared the dissimilarity of this native niche with each grid cell in their expanded range (Broennimann *et al*., 2014).

To evaluate the determinants of the initial spread of tetraploids, we first estimated the residence time of tetraploids in each pixel of the expanded range (i.e. years elapsed since the first record in distinct 10 km × 10 km pixels). This residence time was used as a response variable in a boosted regression tree (BRT) to estimate the relative importance of several predictors for how early or how late distinct pixels got colonized. BRTs are frequently used to identify predictors of species distribution (Elith *et al*., 2008). They are particularly suitable to analyze large datasets with numerous different predictor variables and are relatively insensitive to collinearity and missing values in the predictor variables (Sporbert *et al*., 2022; Rauschkolb *et al*., 2024). We fitted our BRT using the R‐package dismo, assuming a Gaussian error distribution, a bag fraction of training data of 0.5, a tree complexity of 1, a learning rate of 0.01, and a tolerance of 0.01. We then assessed the significance of predictor variables using model simplification based on model‐internal cross‐validations. Our predictor variables included four types of data: (1) spatial data: latitude, longitude and the spatial distance to the native range, (2) climatic data: a precipitation gradient (loadings of the first PCA axis), a temperature gradient (loadings of the second PCA axis) and the climatic distance to the native range (niche dissimilarity from the MESS analysis), (3) dispersal corridor data and (4) urbanization data. The latter two data categories encompassed spatio‐temporally explicit data, extracted from 10 km × 10 km pixels. We used data from 1990 as it was the earliest year with high‐quality data available from all sources. Moreover, by 1990, *c*. half of the pixels had been colonized. The dispersal corridor data included the density of railways (log_e_‐transformed) and roads (log_e_‐transformed), and a connectivity index (log_e_‐transformed). Railway and road density data (km lengths within the 10 km × 10 km pixels) were extracted from the dataset in Garcia‐López *et al*. (2022). The connectivity index was calculated as the reciprocal of the estimated travel time to the nearest city with at least 50 000 inhabitants from the Agglomeration Index database (Uchida & Nelson, 2009). The urbanization data included human population density (log_e_‐transformed) and proportion data of urban vs rural landscape (logit‐transformed), both downloaded from the Global Human Settlement Layer (Schiavina *et al*., 2023), and the percentage of landscape covered by impervious structures (log_e_‐transformed) from the annual maps of global artificial impervious area (Gong *et al*., 2020).

To evaluate the current spread of tetraploids, we first plotted the proportion of tetraploids in the expanded range over time since 1945, that is the onset of the Anthropocene era (Zalasiewicz *et al*., 2015). This plot suggested that the current spread occurs since 1989 (Fig. S4). To analyze the determinants of this current spread, we performed a second BRT, using binomial occurrence data (i.e. diploid vs tetraploid) from 1989 to 2023 as a response variable. This BRT was fitted to the same predictor variables and using the same settings as the previous BRT, except that we assumed a Bernoulli error distribution. Moreover, for the spatio‐temporally explicit predictors related to urbanization and dispersal corridors, we retrieved data from the distinct collection year or used interpolated data for years where no data was available.

## Results

### Tetraploids underwent a cryptic invasion into the range of diploids

**Fig. 1:**
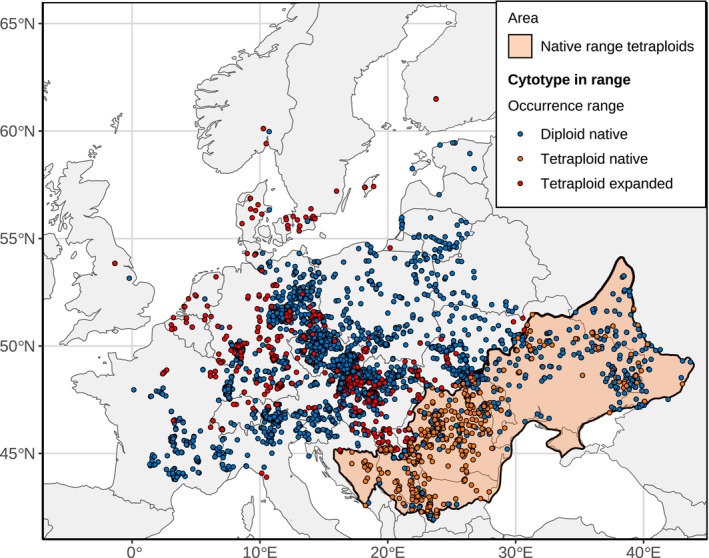
Geographic distribution of diploid and tetraploid *Centaurea stoebe* across the European study range (*n* = 5491). In tetraploids, we differentiate between occurrences in the native (orange dots) and expanded ranges (red dots). In diploids, the entire study range is considered to be part of their native range (blue dots). Occurrences are plotted chronologically with newer occurrences overlapping older ones. The light orange‐colored area indicates the estimated native range of tetraploids. Note that beyond the here presented study range, diploids exhibit a substantially larger range than tetraploids. The complete distribution of both cytotypes across Eurasia is available in Supporting Information Fig. S5. Cartograms illustrating the occurrence data per country are available in Fig. S6.

Our focal dataset included 5465 occurrence data of diploid and tetraploid *C. stoebe* recorded across our European study range between 1790 and 2023 (Fig. 1). This represents the most exhaustive spatio‐temporal dataset on a polyploid complex to date with an exceptional coverage of the sympatric ranges of both cytotypes. Diploids represent the majority cytotype in our focal dataset (3950 diploid and 1533 tetraploid records), however, not taking into account when the specimens were collected. Diploids are native throughout the entire study range.

Tetraploids are not native to large parts of the study range. The native range of tetraploids encompasses mountainous regions in the Balkan countries and Romania. In addition, it includes natural steppes, including forest steppes, stretching from Romania to Western Russia, where the eastern border is delineated by the Don River (see light orange‐colored area in Fig. 1). Tetraploids expanded their range toward Central, Western and Northern Europe. This cryptic invasion resulted in a wide overlap in the distributions of both cytotypes.

### Temporal patterns in the proportion of tetraploids support a distinction between native and expanded ranges

**Fig. 2:**
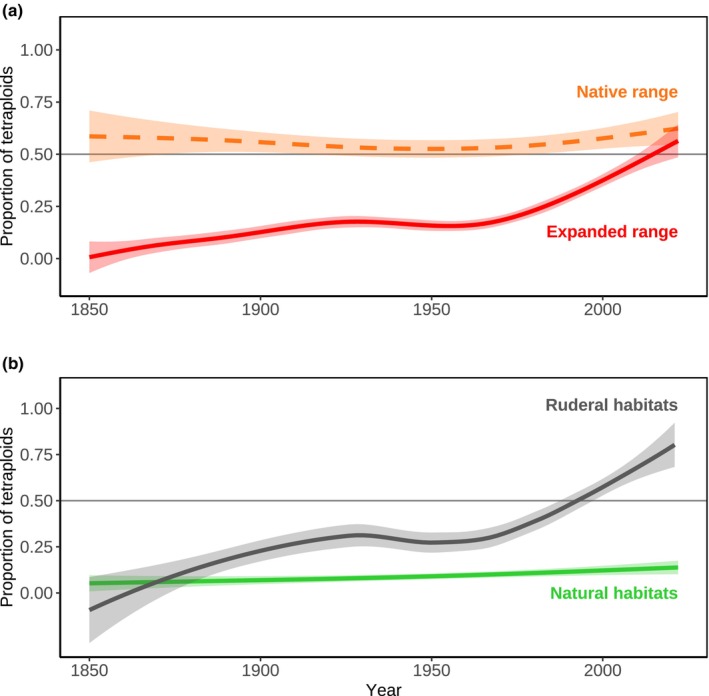
Predicted proportion of tetraploid relative to all *Centaurea stoebe* records as a function of time, range, and habitat type. (a) The proportion of tetraploids in the native (orange) and expanded (red) ranges of tetraploids over time, irrespective of habitat type (*n* = 5491). (b) The proportion of tetraploids in their expanded range over time, differentiated between ruderal (gray) and natural (light green) habitats (*n* = 3340). Model predictions are depicted using generalized additive logistic models, with solid and dashed lines denoting significant and nonsignificant relationships, respectively. The shaded bands show confidence intervals (1σ uncertainty). Thin gray lines indicate a proportion of tetraploids of 50%. Temporal patterns of the proportion of tetraploids in ruderal and natural habitats of the native range of tetraploids are presented in the Supporting Information Fig. S7. Geographical distributions of both cytotypes with respect to habitat types are presented in the Fig. S8.

The temporal patterns of the proportion of tetraploids, relative to all *C. stoebe* records, differed significantly between the native and expanded ranges (ΔAIC = −61, Fig. 2a; Table S5). In the native range, the proportion of tetraploids did not change significantly over time (χ^2^ = 2.4, *P* = 0.124) but stayed at *c*. 50%. By contrast, in the expanded range, the proportion of tetraploids increased significantly over time (χ^2^ = 206.5, *P* < 0.001), rising from 0% of tetraploids in the 1850s to > 50% of tetraploids presently. The contrasting dynamics in both ranges strongly support our distinction between the native and expanded ranges of tetraploids. The cryptic invasion of tetraploids was characterized by three main periods: an initial, modest increase in the proportion of tetraploids from the 1850s to the 1920s, followed by stagnation until the 1950s when a second, exponential increase started, persisting until the present (Fig. 2a). The latter spread coincides with the onset of the Anthropocene, which is an expected temporal pattern for range‐expanding plants (Essl *et al*., 2019).

With respect to the habitat types, we found no differences in the native range between the temporal patterns of the proportion of tetraploids (ΔAIC = +5.7, Table S5): The proportion of tetraploids did not change significantly over time in both ruderal (χ^2^ = 1.8, *P* = 0.176) and natural habitats (χ^2^ = 4.4, *P* = 0.257), but stayed at *c*. 50% in both habitat types (Fig. S7). In the expanded range, however, the temporal dynamics differed significantly between the habitat types (ΔAIC = −6.3, Table S5): In natural habitats, the proportion of tetraploids increased only slightly, even though significantly (χ^2^ = 18.9, *P* < 0.001), rising from 5% in the 1850s to *c*. 10% presently. In ruderal habitats, the proportion of tetraploids increased much stronger over time (χ^2^ = 109.3, *P* < 0.001), rising from 0% in the 1850s to over 75% currently (Fig. 2b). This result shows that the cryptic invasion of tetraploids was primarily due to colonizing ruderal habitats, again representing a typical pattern of recent range expansions (Dainese *et al*., 2017).

### Tetraploids more than doubled their range size

**Fig. 3:**
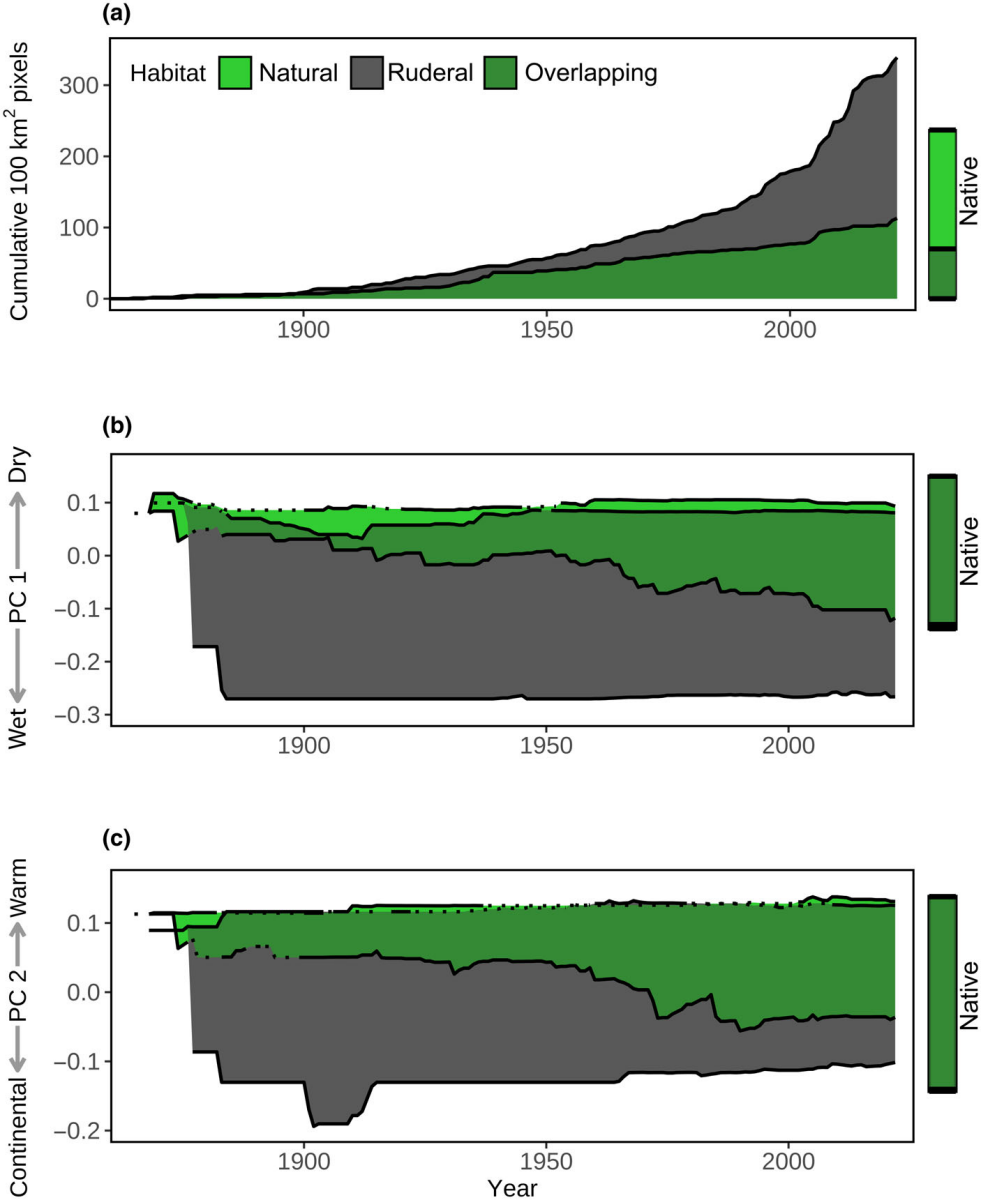
Temporal dynamics of range size and niche breadth of tetraploid *Centaurea stoebe* across its expanded range, with a distinction between ruderal (gray) and natural (light green) habitats (*n* = 744). The habitat‐specific data are visualized as overlapping (dark green), not stacked, areas. (a) Range size dynamics are estimated as a cumulative number of occupied 10 km × 10 km pixels over time (counting first records in distinct pixels). (b) and (c) show the temporal dynamics of the realized niches along a precipitation gradient (the first axis of the PCA, Supporting Information Fig. S3) and along a temperature gradient (second PCA axis), respectively. Lines represent niche limits, with solid and dotted lines indicating significant and nonsignificant differences between populations from ruderal and natural habitats. Reference rectangles on the right side of the plots indicate the range size (a) and niche limits (b, c) of tetraploids in their native range (distinguished between ruderal and natural habitats and their overlap).

Explorative timeline maps spanning 50‐yr intervals showed that there has been no apparent spatial expansion of diploids over time (Fig. S9). By contrast, tetraploids were initially limited to their native range and enlarged this range in the course of their cryptic invasion toward Central Europe. The invasion curves showed that this cryptic invasion more than doubled the range size of tetraploids compared to their native range (+137%) and that tetraploids were much more widespread in ruderal than in natural habitats of their expanded range (+193%, Fig. 3a). We similarly recorded these habitat‐specific invasion curves for tetraploids in their native range, and also for diploids in the expanded range of tetraploids. These two scenarios illustrate range size patterns across the habitat types in native ranges and were diametrically opposed to the cryptic invasion of tetraploids (Fig. S10): Diploids were, within the expanded range of tetraploids, less frequent in ruderal than in natural habitats (−45%). Similarly, tetraploids were in their native range less frequent in ruderal than in natural habitats (−69%). These comparisons indicate that the habitat‐specific patterns in range size development of tetraploids in their expanded range (Fig. 3a) are indicative of a recent colonization rather than merely reflecting habitat availability in Central Europe (see comparison with diploids) or solely reflecting an effect of the ecological niche of tetraploids (see comparison with native tetraploids).

### Tetraploids increased their climatic niche in ruderal habitats

The dynamic range box approach showed that the realized climatic niches were overall comparable between diploids and tetraploids as only 14.3% of their niche spaces did not overlap. However, within tetraploids, the cryptic invasion was associated with a niche shift (62.7% niche nonoverlap). The climate PCA showed that this niche shift went from a continental climate in the native range toward a more oceanic climate in the expanded range (Fig. S3).

The niche‐over‐time plots suggested that the temporal patterns of the niche shift did not mirror the patterns of range size expansion. The climatic niche of tetraploids in their expanded range was largely filled by 1900, when < 5% of the range size was occupied (Fig. 3). However, the niche space was much faster occupied in ruderal than in natural habitats. Presently, ruderal populations still show a substantially broader niche than natural populations (+53%), especially toward wetter climates (+125%). Similarly as we did for the range size dynamics, we explored whether these habitat‐specific niche dynamics were unique to the cryptic invasion of tetraploids by comparing them with the niche dynamics of diploids in the same range and the niche dynamics of tetraploids in their native range. In both native range scenarios, there was no credible evidence for niche differences between the two habitat types (Fig. S11).

### The initial spread of tetraploids was mainly determined by the spatial distance from their native range

**Fig. 4:**
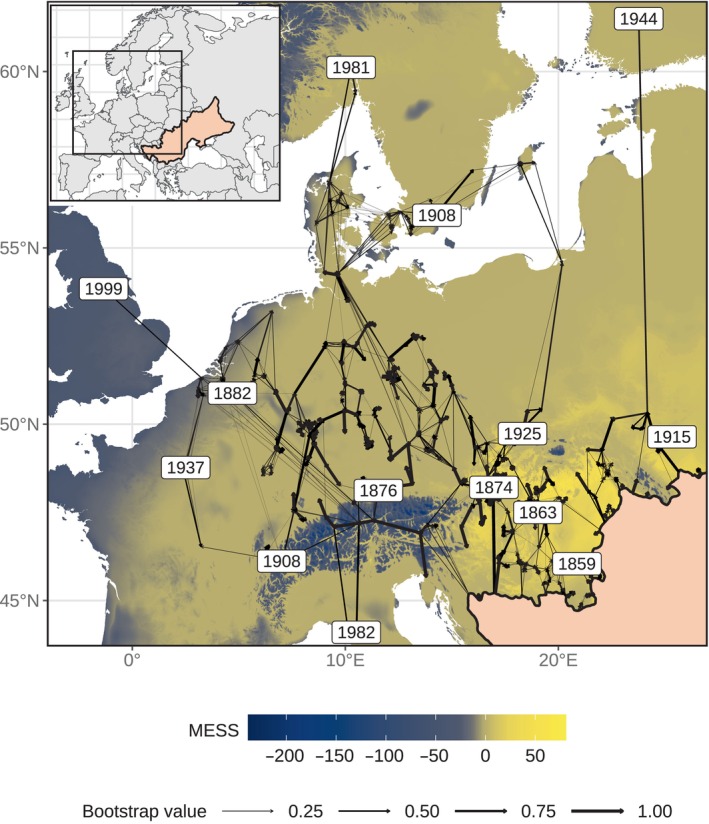
Predicted dispersal routes of the initial spread of tetraploid *Centaurea stoebe* across its expanded range. Dispersal routes were predicted using a minimum cost arborescence algorithm (*n* = 951). Arrows represent these routes with line thicknesses corresponding to their bootstrap support (see legend). The inset map in the upper left corner provides context for the section of the map within Europe. The homogenously light orange‐colored area represents the estimated native range of tetraploids. In the expanded ranges, climatic dissimilarity between the native and expanded ranges was assessed using a multivariate environmental similarity surface (MESS) analysis. Yellow indicates areas with climatic conditions similar to the native niche (positive MESS‐values), while the beige‐blue gradient illustrates the degree of dissimilarity with the climate of the native range (negative MESS‐values, see legend). Mapped years denote when tetraploids were first reported in distinct regions: 1859: Periam, Romania; 1863: Budapest, Hungary; 1874: Devínská Nová Ves, Slovakia; 1876: Munich, Germany; 1882: Louvain, Belgium; 1908: Åhus, Sweden and Salvan, Switzerland; 1915: Ozerna, Ukraine; 1925: Řepiště, Czech Republic; 1937: Paris, France; 1944: Tampere, Finland; 1981: Norderhov, Norway; 1982: Lucca, Italy; 1999: Aberford, United Kingdom.

The minimum cost arborescence algorithm predicted many separate dispersal routes (Fig. 4). The oldest routes went through western Romania (1859), Hungary (1863), Slovakia (1874) and Germany (1876). A MESS analysis revealed no discernible relationship between the predicted spread routes and climatic dissimilarity. For example, many of the climatically suitable regions are still not colonized by tetraploids (e.g. yellow‐beige land north of the native range in Fig. 4).

**Fig. 5:**
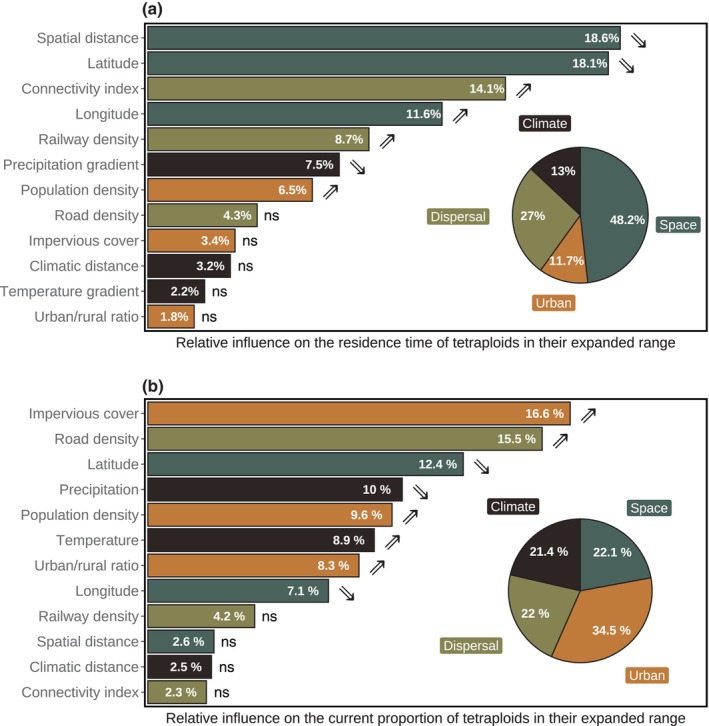
Predictors of the spread of tetraploid *Centaurea stoebe* across its expanded range, assessed with boosted regression trees. (a) Predictors of the initial spread (*n* = 388, cross‐validation correlation (cv) = 0.49). As a proxy of the initial spread, we used the residence time of tetraploids in their expanded range, that is the time since each of 10 km × 10 km pixels had been colonized by tetraploids. (b) Predictors of the current occurrence (*n* = 605, cv = 0.59). As a proxy of the current occurrence, we used the predicted proportion of tetraploid relative to all *C. stoebe* records between 1989 and 2023. Predictors of the two spatio‐temporarily explicit response variables included three variables related to each of four categories: space (dark green), climate (black), dispersal corridors (olive), and urbanization (orange). The pie charts provide an overview of the cumulative contributions of variables categorized by data type. Bar plots show the relative importance of each variable, with upper and lower arrows indicating positive and negative relationships, respectively (‘ns’ denotes no significant effect). For significant predictors, plots of their linear relationships with the residence time or with the proportion of tetraploids can be found in the Supporting Information Figs S13 and S14, respectively.

The BRT analysis showed that spatial data explained more variation in the initial spread of tetraploids than data related to climate, urbanization or dispersal corridors (Fig. 5a). More specifically, the best predictors of the initial spread were the distance to the native range and latitude, both being negatively correlated with the residence time (Fig. S12). In other words, the further localities were away from the native range, the later they had been colonized by tetraploids.

### The current occurrence of tetraploids is mainly associated with urbanization and road‐assisted dispersal

When plotting the spread of tetraploids in the Anthropocene, we found that the proportion of tetraploids increased in the late 1950s, but this increase flattened in the late 1970s (Fig. S4). Since 1989, coinciding with the fall of the iron curtain, and thus increased trade and travel between the native and expanded ranges (Kupková *et al*., 2013), there was another, exponential increase of the proportion of tetraploids, persisting until the present. We, therefore, designated the period since 1989 as representing the time frame of the current spread of tetraploids in their expanded range.

The second BRT analysis revealed that this current spread was more associated with data related to urbanization than with spatial data, dispersal corridor data or climatic data (Fig. 5b). More specifically, the most important predictors of the current occurrence of tetraploids were the cover of impervious structures and the road density, both being positively correlated with the proportion of tetraploids (Fig. S13). This finding aligns with a detailed analysis on different subtypes of ruderal habitats showing that roadsides are currently the most frequently occupied habitat type of tetraploids in their expanded range. In particular, the proportion of roadsides increased from 10% in the 1870s to *c*. 35% at present (Fig. S14). Our field surveys further support these findings, as we recorded 39.3% of the tetraploid populations in the expanded ranges along roadsides (Table S3). Some of these roadside populations appeared to naturalize into adjacent natural and seminatural plant communities (see Figs S15–S17 for field impressions).

## Discussion

Our conceptual framework, considering habitat requirements, phylogeographic data and expert floristic knowledge, represents a unique attempt to delineate the native range of a range‐expanding species. The robustness of our estimation was reinforced by compelling results on the range‐ and habitat‐specific dynamics in the proportion of tetraploids, showing that tetraploid *C. stoebe* is only native to South‐Eastern Europe and has recently expanded its range toward Central Europe. Similar range expansions have been proposed for various plant species, possibly influenced by both climate change and pronounced human activities in Central Europe (Dainese *et al*., 2017; Zhang *et al*., 2023). However, the lack of quantitative assessments on spatio‐temporal range dynamics of these species hampers our ability to identify the overarching drivers behind their ongoing expansions (Essl *et al*., 2019). Further research in other species is needed to improve our understanding on native range expansions in the Anthropocene.

Importantly, our assessment contrasts with 52 studies that treated tetraploid *C. stoebe* populations from the estimated expanded range as native. A careful revision of the spatio‐temporal range dynamics – as we did here – would have been beneficial *before* conducting the samplings of these studies. We consequently encourage researchers to meticulously evaluate the native range of their model species, especially when studying its evolutionary ecology (Lustenhouwer & Parker, 2022). This task may be particularly challenging yet indispensable when dealing with cryptic species, such as cytotypes that show similar morphologies (Šingliarová *et al*., 2011; Nagy *et al*., 2018; Mezhzherin *et al*., 2022). In our study, > 90% of the specimen labels did not differentiate between diploids or tetraploids. Instead, the specimens were stored under various names and frequently misidentified. Unfortunately, most available information on taxa distribution across space and time remains unrevised, representing a pressing problem of biodiversity science (Maldonado *et al*., 2015). We thus stress that both accurate taxonomy and solid knowledge on the native ranges of species are essential for understanding invasion dynamics (Pyšek *et al*., 2013; Skokanová *et al*., 2023) and contemporary biodiversity trends (Hochkirch *et al*., 2021; Lehnert *et al*., 2023).

The cryptic invasion of tetraploids across Central Europe corresponds to the invasion of North America, where exclusively tetraploids were able to establish (Mráz *et al*., 2011). Our research thus supports the observation that polyploids often show greater invasion success than closely related diploids (te Beest *et al*., 2012; Pandit *et al*., 2014). More specifically, our results suggest that tetraploids possess superior colonization abilities over diploids in ruderal habitats, a characteristic that may be particularly prevalent in allopolyploid species like tetraploid *C. stoebe* (Prentis *et al*., 2008; te Beest *et al*., 2012). These superior colonization abilities may relate to the increased longevity of tetraploids, enhancing their tolerance to environmental and demographic stochasticity in ruderal habitats (Mráz *et al*., 2011, 2012b). Moreover, the polycarpic tetraploids show higher re‐sprouting success after severe disturbance (Rosche *et al*., 2018), greater germination success (Kožić *et al*., 2024) and greater life‐span seed production than the monocarpic diploids (Hahn *et al*., 2012). In addition, the genome duplication has been shown to augment adaptive capabilities (Rosche *et al*., 2016) and diminish founder effects (Rosche *et al*., 2016, 2017) in tetraploid compared to diploid *C. stoebe*.

The cryptic invasion of tetraploids involved a niche expansion toward a more oceanic climate. Notably, tetraploids also experienced a niche shift during their North American invasion, but in the opposite direction, toward a more continental climate (Broennimann *et al*., 2014). These intercontinental differences could be mediated by different biotic interactions (Sheng *et al*., 2022; Villasor *et al*., 2024) or the climate conditions prevailing on each continent (Atwater *et al*., 2018; Lee *et al*., 2022). The dynamics of the niche expansion in Europe differed strongly between habitat types. The climatic niche was rapidly occupied in ruderal but not in natural habitats, representing a potentially common, yet understudied, phenomenon in plant invasions (González‐Moreno *et al*., 2015). In the most oceanic regions, natural sites remained uncolonized, probably because they are less suitable for *C. stoebe* (wet conditions with strong interspecific competition) than ruderal sites that could mimic the conditions in the native range (dry with weak interspecific competition).

While the initial spread of tetraploids was determined by the distance to their native range, their current occurrence was predominantly associated with the impervious cover of the landscape and the road density. This result reiterates the crucial role of colonization abilities in ruderal habitats as a primary mechanism for the current success of tetraploid *C. stoebe* (Rosche *et al*., 2016, 2018) and emphasizes the importance of roads as dispersal corridors facilitating range expansions (Follak *et al*., 2018). More generally, our results add to the growing body of research suggesting that polyploidy confers benefits in coping with stressors linked to urbanization (van Drunen & Johnson, 2022; Turcotte *et al*., 2024). Climatic characteristics did not play a major role in either the initial spread or the current occurrence of tetraploids. This finding is consistent with current research concepts emphasizing the growing importance of anthropogenic factors relative to macroclimate as drivers of species distributions (McKeon *et al*., 2023).

In conclusion, we have presented what we believe to be the first robust empirical evidence of a cryptic invasion by a polyploid plant expanding into the range of its diploid relative. Given the increasing accessibility of herbarium collections online (Davis, 2023), we hope to motivate more scientists to critically evaluate the native ranges of their study species. Such endeavors may improve the assessment of biodiversity trends and the design of research studies. Our study also sheds light on the superior colonization ability of tetraploids as a key driver of their cryptic invasion, particularly along roadsides and in habitats with high‐impervious cover. The capacity to occupy such ruderal habitats is crucial in contemporary landscapes because humans are continually increasing loss and fragmentation of natural habitats while increasing the prevalence and connectivity of ruderal habitats (Otto, 2018). More studies on polyploid complexes are warranted to test generality and limitations of our results and to explore the broader implications of our findings for global patterns in the distribution of diploid vs polyploid plants (Rice *et al*., 2019; van Drunen & Johnson, 2022).

Future studies on tetraploid *C. stoebe* could investigate the ecological significance of the proposed native and expanded ranges in Europe. For example, research may test whether the recent range expansion of tetraploids has been accompanied by divergent selection regimes leading to rapid phenotypic changes between native and expanded populations (Lustenhouwer *et al*., 2024; Nagy *et al*., 2024). It would be also interesting to investigate how our proposed range differentiation might influence findings from previous studies, such as the climatic niche shift between the ‘native’ Eurasian and invasive North American ranges.

## Competing interests

None declared.

## Author contributions

CR, OB, AG, HM‐S and PM designed research; CR, AN, VM, GVB, JD, KK, ML, DUN, NMS, MR, TS, AET, PZ and PM performed research; CR, OB, MTG, RR, MR, TS and JAS analyzed data; and CR, OB and PM wrote the paper with substantial input from all co‐authors.

## Supporting information


**Dataset S1** Occurrence database, analyzed in this study.

**Fig. S1** Geographical distribution of the 167 investigated herbaria.
**Fig. S2** Flowchart on creating our dataset.
**Fig. S3** Climatic niche differentiation of diploid and tetraploid *Centaurea stoebe* across the study range in Europe.
**Fig. S4** Predicted proportion of tetraploid relative to all *Centaurea stoebe* records in the ruderal habitats of the expanded range of tetraploids since 1945.
**Fig. S5** Geographical distribution of all *Centaurea stoebe* records.
**Fig. S6** Cartograms illustrating the spatial distribution of diploid and tetraploid *Centaurea stoebe* at the country level across Europe.
**Fig. S7** Predicted proportion of tetraploid relative to all *Centaurea stoebe* records over time within the native range of tetraploids.
**Fig. S8** Habitat preferences of diploid and tetraploid *Centaurea stoebe* across the study range.
**Fig. S9** Range dynamics of diploid and tetraploid *Centaurea stoebe* across 50‐yr time intervals in our study range.
**Fig. S10** Range size over time of diploid and tetraploid *Centaurea stoebe* in their native ranges, distinguished between ruderal and natural habitats.
**Fig. S11** Realized climatic niche breadths over time of diploid and tetraploid *Centaurea stoebe* in their native ranges.
**Fig. S12** Linear predictors of the initial spread of tetraploid *Centaurea stoebe* across its expanded range.
**Fig. S13** Linear predictors of the current occurrence of tetraploid *Centaurea stoebe* across its expanded range.
**Fig. S14** Predicted proportion of ruderal habitat subtypes that have been colonized by tetraploid *Centaurea stoebe* in its expanded range over time.
**Fig. S15** Field impressions from tetraploid *Centaurea stoebe* populations along roadsides in its expanded range.
**Fig. S16** Field impressions from tetraploid *Centaurea stoebe* populations that naturalize into seminatural vegetation in its expanded range.
**Fig. S17** Field impressions from tetraploid *Centaurea stoebe* populations in natural habitats in its native range.
**Notes S1** Details on cytotype determination and its validation.
**Notes S2** Details on the estimation of native and expanded ranges.
**Table S1** Published studies that used tetraploid *Centaurea stoebe* populations from the expanded range and treated them as native populations.
**Table S2** List of the 167 herbaria where the diploid and tetraploid *Centaurea stoebe* herbarium specimens were deposited.
**Table S3** Previously published cytogeographic records of diploid and tetraploid *Centaurea stoebe* populations.
**Table S4** List of unpublished cytogeographic records of *Centaurea stoebe* populations.
**Table S5** Model comparisons of the generalized additive models.
Please note: Wiley is not responsible for the content or functionality of any Supporting Information supplied by the authors. Any queries (other than missing material) should be directed to the *New Phytologist* Central Office.

## Data Availability

The data that support the findings of this study are available in the Supporting Information of this article (Dataset S1). The code for data analysis is available on GitHub (https://github.com/Plant‐Ecology‐Lab/Rosche_2024_New_Phytologist_Centaurea). The sequence data for the ITS1 locus that support the ploidy level estimations are available on zenodo (doi: 10.5281/zenodo.13903690).

## References

[nph20212-bib-0001] Atwater DZ , Ervine C , Barney JN . 2018. Climatic niche shifts are common in introduced plants. Nature Ecology & Evolution 2: 34–43. 29203919 10.1038/s41559-017-0396-z

[nph20212-bib-0002] Bickford D , Lohman DJ , Sodhi NS , Ng PKL , Meier R , Winker K , Ingram KK , Das I . 2007. Cryptic species as a window on diversity and conservation. Trends in Ecology & Evolution 22: 148–155. 17129636 10.1016/j.tree.2006.11.004

[nph20212-bib-0003] Broennimann O , Mráz P , Petitpierre B , Guisan A , Müller‐Schärer H . 2014. Contrasting spatio‐temporal climatic niche dynamics during the eastern and western invasions of spotted knapweed in North America. Journal of Biogeography 41: 1126–1136.

[nph20212-bib-0004] Dainese M , Aikio S , Hulme PE , Bertolli A , Prosser F , Marini L . 2017. Human disturbance and upward expansion of plants in a warming climate. Nature Climate Change 7: 577–580.

[nph20212-bib-0005] Davis CC . 2023. The herbarium of the future. Trends in Ecology & Evolution 38: 412–423. 36549958 10.1016/j.tree.2022.11.015

[nph20212-bib-0006] Delisle F , Lavoie C , Jean M , Lachance D . 2003. Reconstructing the spread of invasive plants: taking into account biases associated with herbarium specimens. Journal of Biogeography 30: 1033–1042.

[nph20212-bib-0007] van Drunen WE , Johnson MTJ . 2022. Polyploidy in urban environments. Trends in Ecology & Evolution 37: 507–516. 35246321 10.1016/j.tree.2022.02.005

[nph20212-bib-0008] Elith J , Leathwick JR , Hastie T . 2008. A working guide to boosted regression trees. Journal of Animal Ecology 77: 802–813. 18397250 10.1111/j.1365-2656.2008.01390.x

[nph20212-bib-0009] Essl F , Dullinger S , Genovesi P , Hulme PE , Jeschke JM , Katsanevakis S , Kühn I , Lenzner B , Pauchard A , Pyšek P *et al*. 2019. A conceptual framework for range‐expanding species that track human‐induced environmental change. Bioscience 69: 908–919.

[nph20212-bib-0010] Follak S , Eberius M , Essl F , Fürdös A , Sedlacek N , Trognitz F . 2018. Invasive alien plants along roadsides in Europe. EPPO Bulletin 48: 256–265.

[nph20212-bib-0011] Garcia‐López M‐À , Pasidis I , Viladecans‐Marsal E . 2022. Congestion in highways when tolls and railroads matter: evidence from European cities. Journal of Economic Geography 22: 931–960.

[nph20212-bib-0012] Gong P , Li X , Wang J , Bai Y , Chen B , Hu T , Liu X , Xu B , Yang J , Zhang W *et al*. 2020. Annual maps of global artificial impervious area (GAIA) between 1985 and 2018. Remote Sensing of Environment 236: 111510.

[nph20212-bib-0013] González‐Moreno P , Diez JM , Richardson DM , Vilà M . 2015. Beyond climate: disturbance niche shifts in invasive species. Global Ecology and Biogeography 24: 360–370.

[nph20212-bib-0014] Hahn MA , Buckley YM , Müller‐Schärer H . 2012. Increased population growth rate in invasive polyploid *Centaurea stoebe* in a common garden. Ecology Letters 15: 947–954. 22727026 10.1111/j.1461-0248.2012.01813.x

[nph20212-bib-0015] Hijmans RJ , Phillips S , Leathwick J , Elith J . 2023. * dismo: species distribution modeling*. R Package v.1.3‐14. [WWW document] URL https://CRAN.R‐project.org/package=dismo [accessed 10th December 2023].

[nph20212-bib-0016] Hochkirch A , Samways MJ , Gerlach J , Böhm M , Williams P , Cardoso P , Cumberlidge N , Stephenson PJ , Seddon MB , Clausnitzer V *et al*. 2021. A strategy for the next decade to address data deficiency in neglected biodiversity. Conservation Biology 35: 502–509. 32656858 10.1111/cobi.13589

[nph20212-bib-0017] Hordijk W , Broennimann O . 2012. Dispersal routes reconstruction and the minimum cost arborescence problem. Journal of Theoretical Biology 308: 115–122. 22706153 10.1016/j.jtbi.2012.06.007

[nph20212-bib-0018] Junker RR , Kuppler J , Bathke AC , Schreyer ML , Trutschnig W . 2016. Dynamic range boxes – a robust nonparametric approach to quantify size and overlap of n‐dimensional hypervolumes. Methods in Ecology and Evolution 7: 1503–1513.

[nph20212-bib-0019] Karger DN , Conrad O , Böhner J , Kawohl T , Kreft H , Soria‐Auza RW , Zimmermann NE , Linder HP , Kessler M . 2017. Climatologies at high resolution for the earth's land surface areas. Scientific Data 4: 170122. 28872642 10.1038/sdata.2017.122 PMC5584396

[nph20212-bib-0020] van Kleunen M , Dawson W , Essl F , Pergl J , Winter M , Weber E , Kreft H , Weigelt P , Kartesz J , Nishino M *et al*. 2015. Global exchange and accumulation of non‐native plants. Nature 525: 100–103. 26287466 10.1038/nature14910

[nph20212-bib-0021] Kožić K , Hartmann M , Callaway RM , Hensen I , Nagy DU , Mráz P , Al‐Gharaibeh MM , Bancheva S , Diaconu A , Danihelka J *et al*. 2024. Performance in the recruitment life stage and its potential contribution to invasive success in the polyploid invader *Centaurea stoebe* . Neobiota 20: 127654.

[nph20212-bib-0022] Kupková L , Bičík I , Najman J . 2013. Land cover changes along the Iron Curtain 1990–2006. Geografie 118: 95–115.

[nph20212-bib-0023] Kuppler J , Höfers MK , Trutschnig W , Bathke AC , Eiben JA , Daehler CC , Junker RR . 2017. Exotic flower visitors exploit large floral trait spaces resulting in asymmetric resource partitioning with native visitors. Functional Ecology 31: 2244–2254.

[nph20212-bib-0024] Kúr P , Gregor T , Jandová M , Mesterházy A , Paule J , Píšová S , Šemberová K , Koutecký P , Ducháček M , Schneeweiss GM . 2023. Cryptic invasion suggested by a cytogeographic analysis of the halophytic *Puccinellia distans* complex (Poaceae) in Central Europe. Frontiers in Plant Science 14: 1249292. 37929170 10.3389/fpls.2023.1249292 PMC10620967

[nph20212-bib-0025] Lang PLM , Willems FM , Scheepens JF , Burbano HA , Bossdorf O . 2019. Using herbaria to study global environmental change. New Phytologist 221: 110–122. 30160314 10.1111/nph.15401 PMC6585664

[nph20212-bib-0026] Lee BR , Miller TK , Rosche C , Yang Y , Heberling JM , Kuebbing SE , Primack RB . 2022. Wildflower phenological escape differs by continent and spring temperature. Nature Communications 13: 7157. 10.1038/s41467-022-34936-9 PMC9684453 36418327

[nph20212-bib-0027] Lehnert M , Monjau T , Rosche C . 2023. Synopsis of Osmunda (royal ferns; Osmundaceae): towards reconciliation of genetic and biogeographic patterns with morphologic variation. Botanical Journal of the Linnean Society 20: 341–364.

[nph20212-bib-0028] Lucas MS , Hensen I , Barratt CD , Callaway RM , Durka W , Lekberg Y , Nagy DU , Onstein RE , Shah MA , van Dam NM *et al*. 2024. Re‐focusing sampling, design and experimental methods to assess rapid evolution by non‐native plant species. Biological Invasions 26: 1327–1343.

[nph20212-bib-0029] Lustenhouwer N , Chaubet TM , Melen MK , van der Putten WH , Parker IM . 2024. Plant–soil interactions during the native and exotic range expansion of an annual plant. Journal of Evolutionary Biology 20: voae040. 10.1093/jeb/voae040 38536056

[nph20212-bib-0030] Lustenhouwer N , Parker IM . 2022. Beyond tracking climate: niche shifts during native range expansion and their implications for novel invasions. Journal of Biogeography 49: 1481–1493.

[nph20212-bib-0031] Maldonado C , Molina CI , Zizka A , Persson C , Taylor CM , Albán J , Chilquillo E , Rønsted N , Antonelli A . 2015. Estimating species diversity and distribution in the era of Big Data: to what extent can we trust public databases? Global Ecology and Biogeography 24: 973–984. 27656106 10.1111/geb.12326 PMC5012125

[nph20212-bib-0032] McKeon CM , Kelly R , Börger L , De Palma A , Buckley YM . 2023. Human land use is comparable to climate as a driver of global plant occurrence and abundance across life forms. Global Ecology and Biogeography 32: 1618–1631.

[nph20212-bib-0033] Mezhzherin SV , Tsyba AA , Kryvokhyzha D . 2022. Cryptic expansion of hybrid polyploid spined loaches Cobitis in the rivers of Eastern Europe. Hydrobiologia 849: 1689–1700.

[nph20212-bib-0034] Morais P , Reichard M . 2018. Cryptic invasions: a review. Science of the Total Environment 613–614: 1438–1448. 10.1016/j.scitotenv.2017.06.133 28648374

[nph20212-bib-0035] Mráz P , Bourchier RS , Treier UA , Schaffner U , Müller‐Schärer H . 2011. Polyploidy in phenotypic space and invasion context: A morphometric study of *Centaurea stoebe* s.l. International Journal of Plant Sciences 172: 386–402.

[nph20212-bib-0036] Mráz P , Garcia‐Jacas N , Gex‐Fabry E , Susanna A , Barres L , Müller‐Schärer H . 2012a. Allopolyploid origin of highly invasive *Centaurea stoebe* s.l. (Asteraceae). Molecular Phylogenetics and Evolution 62: 612–623. 22126902 10.1016/j.ympev.2011.11.006

[nph20212-bib-0037] Mráz P , Španiel S , Keller A , Bowmann G , Farkas A , Šingliarová B , Rohr RP , Broennimann O , Müller‐Schärer H . 2012b. Anthropogenic disturbance as a driver of microspatial and microhabitat segregation of cytotypes of *Centaurea stoebe* and cytotype interactions in secondary contact zones. Annals of Botany 110: 615–627. 22730023 10.1093/aob/mcs120 PMC3400448

[nph20212-bib-0038] Nagy DU , Stranczinger S , Godi A , Weisz A , Rosche C , Suda J , Mariano M , Pal RW . 2018. Does higher ploidy level increase the risk of invasion? A case study with two geo‐cytotypes of *Solidago gigantea* Aiton (Asteraceae). Journal of Plant Ecology 11: 317–327.

[nph20212-bib-0039] Nagy DU , Thoma AE , Al‐Gharaibeh MM , Callaway RM , Flory SL , Frazee LJ , Hartmann M , Hensen I , Jandová K , Khasa DP *et al*. 2024. Among‐population variation in drought responses is consistent across life stages but not between native and non‐native ranges. New Phytologist 243: 922–935. 38859570 10.1111/nph.19895

[nph20212-bib-0040] Novak SJ . 2011. Geographic origins and introduction dynamics. In: Simberloff D , Rejmánek M , eds. Encyclopedia of biological invasions. Berkeley, CA, USA: University of California Press, 273–280.

[nph20212-bib-0041] Ochsmann J . 2000. Morphologische und molekularsystematische Untersuchungen an der *Centaurea stoebe* L.‐Gruppe (Asteraceae‐Cardueae) in Europa. Stuttgart, Germany: J. J. Cramer.

[nph20212-bib-0042] Otto SP . 2018. Adaptation, speciation and extinction in the Anthropocene. Proceedings Biological Sciences, USA 285: 20182047. 10.1098/rspb.2018.2047 PMC6253383 30429309

[nph20212-bib-0043] Pandit MK , White SM , Pocock MJO . 2014. The contrasting effects of genome size, chromosome number and ploidy level on plant invasiveness: a global analysis. New Phytologist 203: 697–703. 24697788 10.1111/nph.12799

[nph20212-bib-0044] van de Peer Y , Ashman T‐L , Soltis PS , Soltis DE . 2021. Polyploidy: an evolutionary and ecological force in stressful times. Plant Cell 33: 11–26. 33751096 10.1093/plcell/koaa015 PMC8136868

[nph20212-bib-0045] Prentis PJ , Wilson JRU , Dormontt EE , Richardson DM , Lowe AJ . 2008. Adaptive evolution in invasive species. Trends in Plant Science 13: 288–294. 18467157 10.1016/j.tplants.2008.03.004

[nph20212-bib-0046] Pyšek P , Hulme PE , Meyerson LA , Smith GF , Boatwright JS , Crouch NR , Figueiredo E , Foxcroft LC , Jarosik V , Richardson DM *et al*. 2013. Hitting the right target: taxonomic challenges for, and of, plant invasions. AoB Plants 5: plt042.

[nph20212-bib-0047] R Core Team . 2024. R: a language and environment for statistical computing. Vienna, Austria: R Foundation for Statistical Computing.

[nph20212-bib-0048] Rauschkolb R , Bucher SF , Hensen I , Ahrends A , Fernández‐Pascual E , Heubach K , Jakubka D , Jiménez‐Alfaro B , König A , Koubek T *et al*. 2024. Spatial variability in herbaceous plant phenology is mostly explained by variability in temperature but also by photoperiod and functional traits. International Journal of Biometeorology 68: 761–775. 38285109 10.1007/s00484-024-02621-9 PMC10963576

[nph20212-bib-0049] Rice A , Šmarda P , Novosolov M , Drori M , Glick L , Sabath N , Meiri S , Belmaker J , Mayrose I . 2019. The global biogeography of polyploid plants. Nature Ecology & Evolution 3: 265–273. 30697006 10.1038/s41559-018-0787-9

[nph20212-bib-0050] Rosche C , Baasch A , Runge K , Brade P , Träger S , Parisod C , Hensen I . 2022. Tracking population genetic signatures of local extinction with herbarium specimens. Annals of Botany 129: 857–868. 35670810 10.1093/aob/mcac061 PMC9292615

[nph20212-bib-0051] Rosche C , Durka W , Hensen I , Mráz P , Hartmann M , Müller‐Schärer H , Lachmuth S . 2016. The population genetics of the fundamental cytotype‐shift in invasive *Centaurea stoebe* s.l.: genetic diversity, genetic differentiation and small‐scale genetic structure differ between cytotypes but not between ranges. Biological Invasions 18: 1895–1910.

[nph20212-bib-0052] Rosche C , Hensen I , Lachmuth S . 2018. Local pre‐adaptation to disturbance and inbreeding‐environment interactions affect colonisation abilities of diploid and tetraploid *Centaurea stoebe* . Plant Biology 20: 75–84. 28921779 10.1111/plb.12628

[nph20212-bib-0053] Rosche C , Hensen I , Mráz P , Durka W , Hartmann M , Lachmuth S . 2017. Invasion success in polyploids: the role of inbreeding in the contrasting colonization abilities of diploid versus tetraploid populations of *Centaurea stoebe* s.l. Journal of Ecology 105: 425–435.

[nph20212-bib-0054] Rüegg S , Raeder U , Melzer A , Heubl G , Bräuchler C . 2017. Hybridisation and cryptic invasion in *Najas marina* L. (Hydrocharitaceae)? Hydrobiologia 784: 381–395.

[nph20212-bib-0055] Schiavina M , Melchiorri M , Pesaresi M , Politis P , Carneiro Freire SM , Maffenini L , Florio P , Ehrlich D , Goch K , Carioli A *et al*. 2023. GHSL data package 2023. Luxembourg City, Luxembourg: Publications Office of the European Union.

[nph20212-bib-0056] Sessa EB . 2019. Polyploidy as a mechanism for surviving global change. New Phytologist 221: 5–6. 30488604 10.1111/nph.15513

[nph20212-bib-0057] Sheng M , Rosche C , Al‐Gharaibeh MM , Bullington LS , Callaway RM , Clark T , Cleveland CC , Duan W , Flory SL , Khasa DP *et al*. 2022. Acquisition and evolution of enhanced mutualism—an underappreciated mechanism for invasive success? The ISME Journal 16: 2467–2478. 35871251 10.1038/s41396-022-01293-w PMC9561174

[nph20212-bib-0058] Šingliarová B , Hodálová I , Mráz P . 2011. Biosystematic study of the diploid‐polyploid *Pilosella alpicola* group with variation in breeding system: patterns and processes. Taxon 60: 450–470.

[nph20212-bib-0059] Skokanová K , Španiel S , Šingliarová B , Mereďa P Jr , Hodálová I , Svitok M . 2023. Contrasting invasion patterns of two closely related Solidago alien species. Journal of Biogeography 51: 1–14.

[nph20212-bib-0060] Sporbert M , Jakubka D , Bucher SF , Hensen I , Freiberg M , Heubach K , König A , Nordt B , Plos C , Blinova I *et al*. 2022. Functional traits influence patterns in vegetative and reproductive plant phenology – a multi‐botanical garden study. New Phytologist 235: 2199–2210. 35762815 10.1111/nph.18345

[nph20212-bib-0061] Te Beest M , Le Roux JJ , Richardson DM , Brysting AK , Suda J , Kubesová M , Pyšek P . 2012. The more the better? The role of polyploidy in facilitating plant invasions. Annals of Botany 109: 19–45. 22040744 10.1093/aob/mcr277 PMC3241594

[nph20212-bib-0062] Theoharides KA , Dukes JS . 2007. Plant invasion across space and time: factors affecting nonindigenous species success during four stages of invasion. New Phytologist 176: 256–273. 17822399 10.1111/j.1469-8137.2007.02207.x

[nph20212-bib-0063] Turcotte MM , Kaufmann N , Wagner KL , Zallek TA , Ashman T‐L . 2024. Neopolyploidy increases stress tolerance and reduces fitness plasticity across multiple urban pollutants: support for the “general‐purpose” genotype hypothesis. Evolution Letters 20: qrad072. 10.1093/evlett/qrad072 PMC11134461 38818423

[nph20212-bib-0064] Uchida H , Nelson A . 2009. Agglomeration index: towards a new measure of urban concentration. In: Beall J , Guha‐Khasnobis B , Ravi Kanbur SM , eds. Urbanization and development: multidiciplinary perspectives. Oxford, UK: Oxford University Press, 41–59.

[nph20212-bib-0065] Villasor C , Robertson K , Becker T , Cahill JF , Deák B , Hensen I , Otfinowski R , Rosche C , Borovyk D , Vakhlamova T *et al*. 2024. Invasion success of three cool‐season grasses in the northern prairie: a test of three hypotheses. Oikos 2024: e10266.

[nph20212-bib-0066] Wood SN . 2011. Fast stable restricted maximum likelihood and marginal likelihood estimation of semiparametric generalized linear models. Journal of the Royal Statistical Society, Series B: Statistical Methodology 73: 3–36.

[nph20212-bib-0067] Wood SN , Pya N , Säfken B . 2016. Smoothing parameter and model selection for general smooth models. Journal of the American Statistical Association 111: 1548–1563.

[nph20212-bib-0068] Zalasiewicz J , Waters CN , Williams M , Barnosky AD , Cearreta A , Crutzen P , Ellis E , Ellis MA , Fairchild IJ , Grinevald J *et al*. 2015. When did the Anthropocene begin? A mid‐twentieth century boundary level is stratigraphically optimal. Quaternary International 383: 196–203.

[nph20212-bib-0069] Zhang Z , Yang Q , Fristoe TS , Dawson W , Essl F , Kreft H , Lenzner B , Pergl J , Pyšek P , Weigelt P *et al*. 2023. The poleward naturalization of intracontinental alien plants. Science Advances 9: eadi1897. 37792943 10.1126/sciadv.adi1897 PMC10550228

