# Supplementary material for: Herbarium specimens reveal a cryptic invasion of polyploid Centaurea stoebe in Europe
Source: New Phytol. 2024 Oct 23;245(1):392–405. doi: 10.1111/nph.20212 (PMC11617643; doi:10.1111/nph.20212)
Supplement: Supplementary file 2 — Fig. S1 Geographical distribution of the 167 investigated herbaria. Fig. S2 Flowchart on creating our dataset. Fig. S3 Climatic niche differentiation of diploid and tetraploid Centaurea stoebe across the study range in Europe. Fig. S4 Predicted proportion of tetraploid relative to all Centaurea stoebe records in the ruderal habitats of the expanded range of tetraploids since 1945. Fig. S5 Geographical distribution of all Centaurea stoebe records. Fig. S6 Cartograms illustrating the spatial distribution of diploid and tetraploid Centaurea stoebe at the country level across Europe. Fig. S7 Predicted proportion of tetraploid relative to all Centaurea stoebe records over time within the native range of tetraploids. Fig. S8 Habitat preferences of diploid and tetraploid Centaurea stoebe across the study range. Fig. S9 Range dynamics of diploid and tetraploid Centaurea stoebe across 50‐yr time intervals in our study range. Fig. S10 Range size over time of diploid and tetraploid Centaurea stoebe in their native ranges, distinguished between ruderal and natural habitats. Fig. S11 Realized climatic niche breadths over time of diploid and tetraploid Centaurea stoebe in their native ranges. Fig. S12 Linear predictors of the initial spread of tetraploid Centaurea stoebe across its expanded range. Fig. S13 Linear predictors of the current occurrence of tetraploid Centaurea stoebe across its expanded range. Fig. S14 Predicted proportion of ruderal habitat subtypes that have been colonized by tetraploid Centaurea stoebe in its expanded range over time. Fig. S15 Field impressions from tetraploid Centaurea stoebe populations along roadsides in its expanded range. Fig. S16 Field impressions from tetraploid Centaurea stoebe populations that naturalize into seminatural vegetation in its expanded range. Fig. S17 Field impressions from tetraploid Centaurea stoebe populations in natural habitats in its native range. Notes S1 Details on cytotype determination and its validation. Notes S2 De [file NPH-245-392-s001.pdf]

New Phytologist Supporting Information

Article title: Herbarium specimens reveal a cryptic invasion of polyploid *Centaurea stoebe* in Europe

Authors: Christoph B. Rosche, Olivier Broennimann, Andriy Novikov, Viera Mrázová, Ganna V. Boiko, Jiří Danihelka, Michael T. Gastner, Antoine Guisan, Kevin Kožić, Marcus Lehnert, Heinz Müller-Schärer, Dávid U. Nagy, Ruben Remelgado, Michał Ronikier, Julian A. Selke, Natalia M. Shiyan, Tomasz Suchan, Arpad E. Thoma, Pavel Zdvořák, and Patrik Mráz

Article acceptance date: 1 October 2024.

The following Supporting Information is available for this article:

Supplementary Note S1. Details on cytotype determination and its validation.

Supplementary Note S2: Details on the estimation of native and expanded ranges.

Supplementary Fig. S1. Geographical distribution of the 167 investigated herbaria.

Supplementary Fig. S2. Flowchart on creating our dataset.

Supplementary Fig. S3. Climatic niche differentiation of diploid and tetraploid *Centaurea stoebe* across the study range in Europe.

Supplementary Fig. S4. Predicted proportion of tetraploid relative to all *Centaurea stoebe* records in the ruderal habitats of the expanded range of tetraploids since 1945.

Supplementary Fig. S5. Geographical distribution of all *Centaurea stoebe* records.

Supplementary Fig. S6. Cartograms illustrating the spatial distribution of diploid and tetraploid *Centaurea stoebe* at the country level across Europe.

Supplementary Fig. S7. Predicted proportion of tetraploid relative to all *Centaurea stoebe* records over time within the native range of tetraploids.

Supplementary Fig. S8. Habitat preferences of diploid and tetraploid *Centaurea stoebe* across the study range.

Supplementary Fig. S9. Range dynamics of diploid (blue dots) and tetraploid *Centaurea stoebe* across 50-year time intervals in our study range.

Supplementary Fig. S10. Range size over time of diploid and tetraploid *Centaurea stoebe* in their native ranges, distinguished between ruderal (gray) and natural (light green) habitats.

Supplementary Fig. S11. Realized climatic niche breadths over time of diploid and tetraploid *Centaurea stoebe* in their native ranges.

Supplementary Fig. S12. Linear predictors of the initial spread of tetraploid *Centaurea stoebe* across its expanded range.

Supplementary Fig. S13. Linear predictors of the current occurrence of tetraploid *Centaurea stoebe* across its expanded range.

Supplementary Fig. S14. Predicted proportion of ruderal habitat subtypes that have been colonized by tetraploid *Centaurea stoebe* in its expanded range over time.

Supplementary Fig. S15. Field impressions from tetraploid *Centaurea stoebe* populations along roadsides in its expanded range.

Supplementary Fig. S16. Field impressions from tetraploid *Centaurea stoebe* populations that naturalize into semi-natural vegetation in its expanded range.

Supplementary Fig. S17. Field impressions from tetraploid *Centaurea stoebe* populations in natural habitats in its native range.

Supplementary Table S1. Published studies that used tetraploid *Centaurea stoebe* populations from the expanded range and treated them as native populations.

Supplementary Table S2. List of the 167 herbaria where the diploid and tetraploid *Centaurea stoebe* herbarium specimens were deposited.

Supplementary Table S3. Previously published cytogeographic records of diploid and tetraploid *Centaurea stoebe* populations.

Supplementary Table S4. List of unpublished cytogeographic records of *Centaurea stoebe* populations.

Supplementary Table S5. Model comparisons of the generalized additive models (GAMs).

Supplementary References

Supplementary Note S1. Details on cytotype determination and its validation.

The cytotype determination of the *Centaurea stoebe* herbarium specimens was carried out using the identification key from Mráz *et al.* (2011). This key primarily relies on distinguishing cytotypes based on the shape of the capitula and the presence/absence of accessory rosettes (Note S1: Fig. N1.1). It is important to note that the cytotypes show overlapping morphological variation making cytotype discrimination a challenging task (Mráz *et al.*, 2011). Therefore, all cytotype identifications were done by P. Mráz, a dedicated plant taxonomist with extensive experience in the morphological variation of *C. stoebe* in the wild and in experimental cultivation.

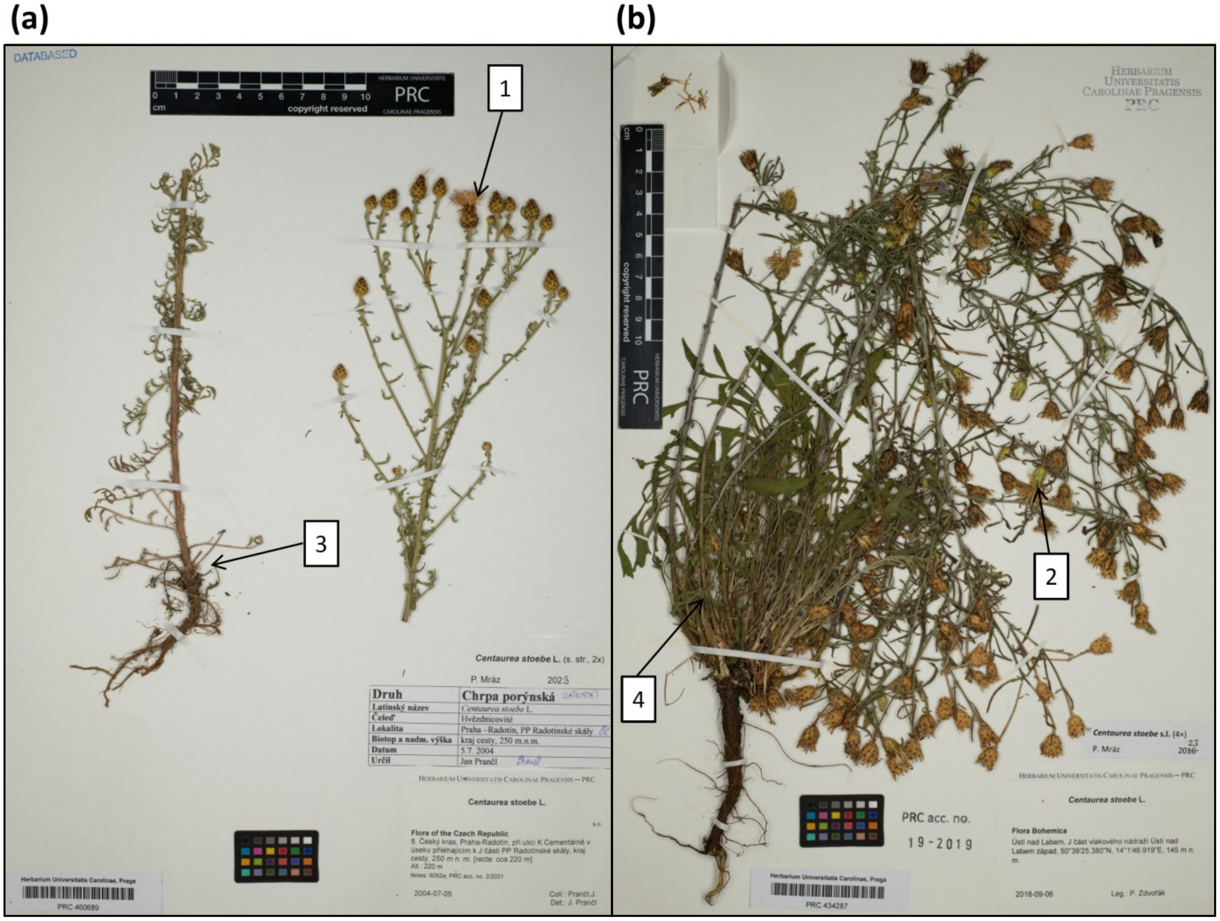

Supplementary Fig. N1.1. Exemplary herbarium specimens of (a) diploid and (b) tetraploid *Centaurea stoebe*. Cytotype determination followed the identification key in Mráz *et al.* (2011). The cytotypes can be distinguished by the combination of the following key traits that are indicated by the numbers in the boxes next to the arrows. [1] The shape of the capitula is more rounded in diploids, with an average length/width ratio of 1.2, whereas in tetraploids, [2] it is more elongated with an average length/width ratio of 1.35. [3] Diploid plants are annual or biennial without formation of overwintering accessory rosettes after withering of the shoot(s) and are usually one- to few-stemmed. [4] Tetraploid plants are short-lived perennial, often forming overwintering accessory rosettes after withering of the shoot(s) and are frequently few- to many-stemmed.

To assess whether there was a geographical bias in our morphological determination of cytotypes, we compared a distribution map based on our revised herbarium specimens with a map based on available cytogeographic data. For both cytotypes, we observed consistent distribution patterns between the two data sources (Note S1: Fig. N1.2).

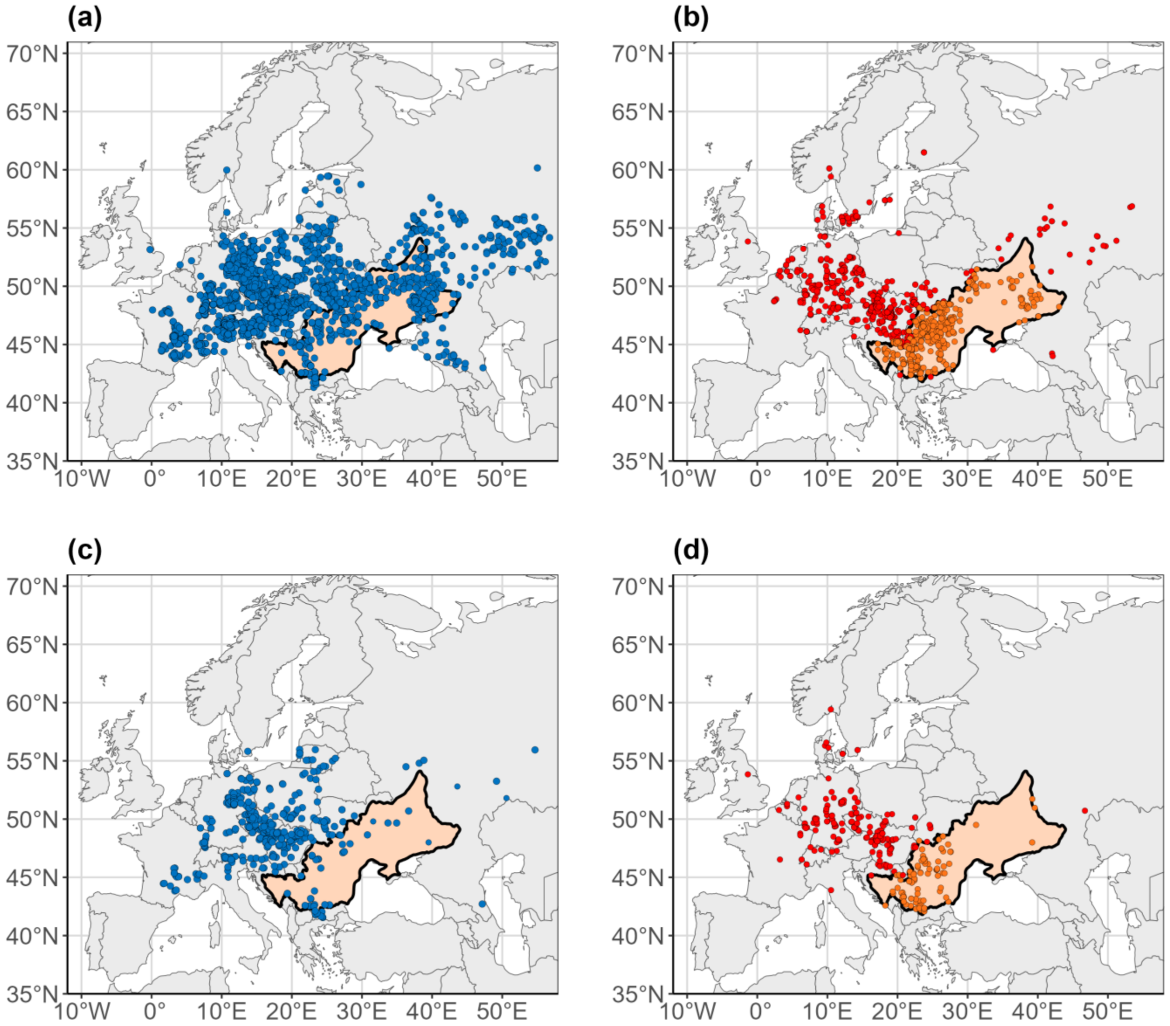

Supplementary Fig. N1.2. Visual comparison between two sources of the records of *Centaurea stoebe*. (a and b) herbarium specimen records from which the cytotype had been identified by morphology vs. (c and d) records from which the cytotype had been identified by flow cytometry measurements or chromosome counts. The records are shown for diploids (blue dots) and tetraploids across their sympatric European range (i.e., from the total dataset, see Fig. S2 for details on the dataset). In tetraploids, occurrences in the native (orange dots) and expanded ranges (red dots) are distinguished. The light orange-colored area represents the estimated native range of tetraploids. Occurrences are plotted chronologically with newer occurrences overlapping older ones. Upper panels present herbarium specimen of (a) diploids and (b) tetraploids. Lower panels present cytogeographic data (i.e., data that have been validated with chromosome counts or flow cytometry) for (c) diploids and (d) tetraploids. Comparing upper and lower panels suggests that the visual determination of herbarium specimen resulted in a similar distribution as that of lab-confirmed cytogeographic data. The sources of herbarium data are provided in Table S2 and Fig. S1. The sources of cytogeographic data are given in Tables S3 and S4.

We then used two molecular approaches to validate the accuracy of the morphology-based determination. First, we made use of a formerly established experimental plot at the University of Fribourg (Switzerland) with an artificial mixed-cytotype population. This population was created for a demography experiment by Hahn *et al.* (2012a), established in 2009 using offspring from 12 diploid and 22 tetraploid populations, randomly distributed across the experimental plot. The experiment was finished in 2010 and the experimental plot was abandoned. The remaining experimental plants and their offspring built a self-perpetuating mixed-cytotype population. After six years, this artificial mixed-cytotype population was revisited, consisting of 463 individuals at this time. Ploidy level was morphologically assigned to each of the 463 individuals by P. Mráz. Subsequently, ploidy level of all individuals was verified using flow cytometry according to the method by Mráz *et al.* (2011). The accuracy rate of morphological determination was 98.3%. The accuracy rate of morphological determination was 98.3%.

Second, we genotyped 178 herbarium specimens after morphological determination of their cytotype (see Note S1: Table N1.1 for details on ITS1 amplification, sequencing, bioinformatics and focal nucleotide positions for discriminating ribotypes). We choose this subsample (3.5% of the total number of specimens) to represent comparable distributional ranges and collection dates for both cytotypes (see Note S1: Fig. N1.3 for specimen distribution). Details on the sampled specimens are given in the table *Centaurea_stoebe_ITS_sample_list.xlsx* which is publicily available on zenodo (https://doi.org/10.5281/zenodo.13894116). The sequences of the specimens are also deposited at this link. A posteriori comparison of sequence-based and morphology-based assignments showed 97.8% congruence. In conclusion, our complementary approaches demonstrated that the visual, morphology-based determination approach was very reliable and consistent across the investigated ranges.

Supplementary Table N1.1. Amplification, sequencing and bioinformatics of the ITS1-region.

Step	Details on procedure
DNA extraction	DNA was isolated from 10 mg of dry leaf material per sample using the Qiagen DNeasy Mini Kit (Qiagen, Hilden, Germany), according to manufacturer's instructions.
ITS locus	Genotyping of nuclear ribosomal ITS1 locus was performed using a metabarcoding approach that consisted of two PCR steps. PCR products were screened for two ribotypes: ribotype A and ribotype B sensu Mráz *et al.* (2012a) who found that ribotype A was present in all diploid and tetraploid samples whereas ribotype B occurred only in tetraploid *C. stoebe*.
PCR 1	The library construction followed Suchan *et al.* (2019), using Trac01 primers to amplify the ITS1-locus. These primers were tailed with a technical sequence (i.e., including a partial Illumina adapter).
PCR 2	Double-indexing Illumina primers were used on this template to uniquely tag sequences belonging to each specimen.
Avoidance of contamination	Reactions were prepared under laminar-flow hood that was UV and 10% bleach-decontaminated. Filter tips were used for all liquid handling. Thirteen blank samples were used to control for contamination during lab procedures.
Checking PCR products and library quantification	Final reaction products were checked on 1.5% agarose gel, pooled and purified using AMPure XP (Beckman Coulter, Indianapolis, IN, U.S.A.) at 1x ratio of the PCR product to AMPure XP. Pooled and purified libraries were checked on TapeStation 4200 (Agilent Technologies, Santa Clara, CA, U.S.A.) and quantified using Qubit fluorometer (Thermo Fisher Scientific, Waltham, MA, U.S.A).
Sequencing	Final reaction products were sequenced with 15% PhiX spike-in on a MiSeq sequencer (Illumina, San Diego, CA, U.S.A.) using the 500-cycle MiSeq Reagent Kit v2 according to the manufacturer's instructions.
Sequence merging	The obtained sequences were merged using PEAR 0.9.6 (Zhang *et al.*, 2014). Only sequences were kept for which the overlapping paired-end reads could be merged.
Sequence trimming	Primer sequences were trimmed with CUTADAPT 3.2 (Martin, 2011) using the linked adapters option. Only fragments that had full primer sequences on both ends were trimmed and kept for the next steps.
Read filtering and clustering	Reads were filtered and clustered using VSEARCH 2.15.2. Filtering based on the expected error (maxee) = 1, minimum length = 250, and a maximum number of undetermined nucleotides = 0. The filtered reads were dereplicated, which collapses identical reads into a single representative read and generates a count of each unique sequence. The reads obtained from all the samples were then combined together, dereplicated and clustered into operational taxonomic units (OTUs) using the UNOISE3 algorithm (Edgar, 2016). OTUs were cleaned from chimeras using the UCHIME3 algorithm and the original reads were mapped to the OTUs using the usearch_global algorithm to generate an OTU table. We aligned the reads with the MAFFT L-INS-i method (Katoh & Toh, 2008). In total, we considered twenty sequence variants in the analysis. Criteria for consideration were that the variants assigned to *C. stoebe* and had over 1000 reads. Within a sample, all variants with frequency exceeding 1% of total reads were retained.
Focal nucleotide positions	For discriminating ribotypes, we considered the nucleotide positions 108 and 230 which define ribotype A (108: G and 230: T) and ribotype B (108: A and 230: C). Note that these positions translate to positions 77 and 199 in Mráz *et al.* (2012a).
Result	Ribotype B and its minor derivatives were found in 72 genomes. These 72 samples have been identified as tetraploid. By contrast, 109 samples did not show ribotype B variants and were considered diploid.

Note: Distribution of diploid and tetraploid *Centaurea stoebe* herbarium specimens used for ITS1 sequencing is presented in Note S1: Fig. N1.3.

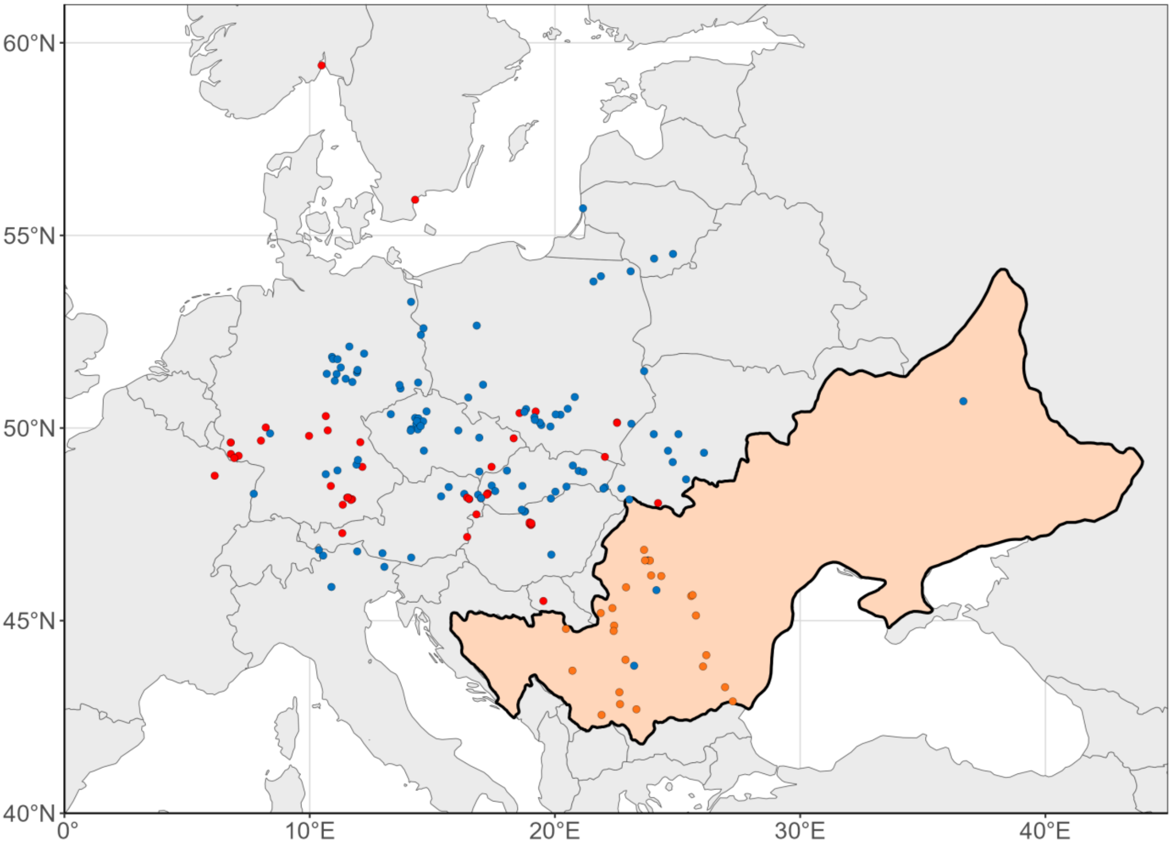

Supplementary Fig. N1.3. Collection sites of 178 diploid (blue) and tetraploid *Centaurea stoebe* herbarium specimens used for sequencing of the ITS1-locus (nuclear ribosomal DNA). In tetraploids, occurrences in the native (orange dots) and expanded ranges (red dots) are distinguished. The light orange-colored area represents the estimated native range of tetraploids. Occurrences are plotted chronologically with newer occurrences overlapping older ones. Sequencing was performed for the sake of molecular cytotype confirmation based on the known ribotype differentiation between the cytotypes (Mráz *et al.*, 2012a). We used specimens from the following herbaria: BNL (10 specimens), HAL (15 specimens), KRAM (33 specimens), M (30 specimens), O (1), PRC (79 specimens) and SAAR (10 specimens). Herbarium acronyms can be found in Table S2. Details on ITS1 amplification, sequencing and bioinformatics can be found in Note S1: Table N1.1.

Supplementary Note S2: Details on the estimation of native and expanded ranges.

Identifying native range expansions and cryptic invasions is inherently difficult, but their correct recognition is crucial to understand their determinants and consequences (Morais & Reichard, 2018; Lustenhouwer & Parker, 2022). In developing our conceptual framework, we aimed for a standardized and neutral approach for distinguishing the native and expanded ranges of tetraploids. We combined three approaches to delineate the native and expanded ranges of tetraploids: 1) We conducted an extensive literature survey of publications proposing a non-native origin of distinct tetraploid *C. stoebe* populations in Central, Western and Northern Europe*.* 2) We considered available phylogeographic datasets of the polyploid complex of *C. stoebe* and closely related taxa. 3) We defined what we understand as natural habitats of tetraploids and assigned geographical regions where tetraploids occur in such habitats as native range.

1. *Survey of literature commenting on the native range of tetraploids*

Combining occurrence data from herbarium specimens with information gleaned from local floristic publications is a suitable approach to uncover range expansions (D'Andrea *et al.*, 2009; Lustenhouwer & Parker, 2022). The first publication that explicitly postulated that a distinct European population of tetraploid *C. stoebe* is not native was Vollmann (1914), who documented its establishment in Munich, Germany. Note that the routine ploidy level analyses, i.e., chromosome counting, were infrequent during this period. However, Vollmann (1914) described plants morphologically corresponding to the tetraploid cytotype and assigned them to a different taxon ("*C. maculosa* ssp. *micrantha*") than diploids ("*C. maculosa* ssp. *rhenana*"). In the following decades, many other botanists reported tetraploid populations outside their native range from various regions of Europe (Note S2: Table N2.1). Whenever the European expansion of tetraploids was discussed in the reviewed literature, the common opinion was that tetraploids are native to the South-Eastern Europe and from there expanded toward Central Europe throughout the last two centuries (e.g., Vollmann, 1914; Welss *et al.*, 2008; Otisková *et al.*, 2014; Klotz, 2015; Rosche *et al.*, 2016). This opinion was particularly supported by Ochsmann (2000), who provided the most detailed, but still rough geographical hypothesis about the native and expanded ranges of European tetraploids. No publication presented a clear definition of the native vs. expanded ranges of tetraploids so far. Note that there was no indication that any parts of the European distribution of diploids could be non-native in the reviewed literature.

Supplementary Table N2.1. Non-exhaustive collection of reports suggesting that local tetraploid *Centaurea stoebe* populations from Central, Western and Northern Europe are not native.

Region	Publication
Vienna, Austria	Rechinger (1950)
Lower Austria, Austria	Kiehn *et al.* (1991)
Skåne, Sweden	Tyler (1999)
Yorkshire, UK	Taylor (2000), Clement (2002)
Czechia	Otisková *et al.* (2014)
Netherlands	Tamis (2005)
Hessia, Germany	Gregor (2001), Becker (2005), Malten (2005), Gregor & Hand (2008)
Saxony-Anhalt, Germany	John & Stolle (2006; 2007), Brennenstuhl (2020)
Thuringia, Germany	Zündorf *et al.* (2006), Klug (2007)
Bavaria, Germany	Vollmann (1914), Welss *et al.* (2008), Meierrott (2008), Klotz (2015)
Saxony, Germany	Gutte & Fischer (2012)
Brandenburg, Germany	Landeck *et al.* (2013)
Rhine valley, Germany	Korneck (2004; 2006; 2016)
Baden-Württemberg, Germany	Adler *et al.* (2017)
Saarland, Germany	Schneider *et al.* (2020)

Note that for the early records (e.g., Vollmann, 1914), ploidy analyses were not yet developed during this period. However, some botanist described plants morphologically corresponding to the tetraploid cytotype and explicitly assigned them to a different taxon.

2. *Phylogeographic considerations on the native range of tetraploids*

Analyzing spatial patterns of contemporary genetic diversity is a conventional method for identifying signatures of recent or historic cryptic invasions (reviewed by Morais & Reichard, 2018). For tetraploid and diploid *C. stoebe* and closely related taxa, there have been molecular studies on ribosomal DNA sequences (Mráz *et al.*, 2012a; Mráz *et al.*, unpubl. data), plastid DNA sequences (Hufbauer & Sforza, 2008; Mráz *et al.*, 2012a; Kožić *et al.*, unpubl. data), amplified fragment length polymorphisms (Kožić *et al.*, unpubl. data) and nuclear microsatellites (Marrs *et al.*, 2008; Mráz *et al.*, 2012b; Rosche *et al.*, 2016). Together these studies indicate a hybridogeneous origin of tetraploids which arose multiple times. Within tetraploid *C. stoebe*, rare alleles were found to be more frequent in South-Eastern Europe than in Central and Western Europe (Rosche *et al.*, 2016; Kožić *et al.*, unpubl. data), which indicates that the latter region may have been colonized rather recently (Schönswetter & Tribsch, 2005). Note that diploids did not show such biogeographical differences in the distribution of rare alleles (Rosche *et al.*, 2016; Kožić *et al.*, unpubl. data). Isolation by distance characterized the geographic patterns of genetic differentiation in diploids but was not apparent in tetraploids (Marrs *et al.*, 2008; Rosche *et al.*, 2016; Kožić *et al.*, unpubl. data). The lack of isolation by distance in tetraploids may result from a rather recent spread across their European range (Rosche *et al.*, 2016).

With regard to the allotetraploid genome, Mráz *et al.* (2012a) estimated that ribotype A arose 1.4–5.2 mya, while ribotype B is younger and arose 0.6–2.5 mya. Ribotype B has not been found in diploid but in tetraploid *C. stoebe* (see Note S1: Table N1.1 for details), and in addition, in another two tetraploid *Centaurea* species occurring in natural habitats in the region of the Black Sea: *C. sarandinikae* (Mráz *et al.*, 2012a) and *C. arenaria* (Mráz *et al.*, unpubl. data). *Centaurea sarandinikae* is a morphologically distinct species from the *C. stoebe* group and is endemic to Crimea and NW Ciscaucasia (Dobrochayeva, 1965; Ryff, 2009). *Centaurea arenaria* is morphologically close to tetraploid *C. stoebe* and shows a larger distributional range which is centered on the region of the Black Sea (Ochsmann, 2000). Thus, the geographical distribution of ribotype B indicates that the steppic areas around the Black Sea are likely the evolutionary cradle of taxa bearing ribotype B, including tetraploid *C. stoebe* (Mráz *et al.*, 2012a).

3. *Conceptual framework of the native and expanded ranges of tetraploids based on their occurrence in zonal steppes and relict habitats*

Previous studies that used occurrence data to delineate native and expanded ranges have typically focused on spatio-temporal distribution patterns. Specifically, regions where occurrences had been recorded before or after a certain temporal threshold were regarded as being part of the native and expanded range, respectively (Morais & Reichard, 2018; Lustenhouwer & Parker, 2022). However, this approach can be strongly biased by spatio-temporal patterns in collection efforts (reviewed by Lang *et al.*, 2019). Instead of relying on a spatio-temporal approach, we focused on distribution patterns in natural habitats, independently of collection time.

We first defiened which type of natural habitats currently harbor tetraploids and may have already existed in the recent past (Essl *et al.*, 2019). For the light-demanding *C. stoebe* (Ellenberg indicator value for light = 8; Ellenberg *et al.*, 1992), this definition encompasses habitats that were permanently open (i.e., not covered by forest vegetation) before strong human activities completely transformed the European landscape. These transformations began during the Neolithic Age (approx. 6,000 BCE in Europe) and include especially the time since the Industrial Revolution (from 1760), including the Anthropocene (from 1945; Zalasiewicz *et al.*, 2015). The mentioned events caused massive deforestation, unprecedented rate of transportation of goods and people, widespread agricultural usage and the founding of human settlements (Kaplan *et al.*, 2009). While any range expansion of tetraploids before the Industrial Revolution is difficult to estimate, we have clear evidence from numerous publications (e.g., Note S2: Table N2.2), our own field surveys (Tables S3 & S4), and our data presented in the main manuscript that the spread of tetraploids across Central and Western Europe happened during the last 200 years and was strongly linked to anthropogenic activities.

Before the mentioned anthropogenic landscape transformation, large-scale canopy-free habitats in Europe include zonal steppes and forest steppes. For reference on the distribution of potential natural vegetation in Europe, we relied on works by Olson *et al.* (2001), Bohn & Gollub (2006), and Ssymank (2013). These sources indicated that climatically determined steppes and forest steppes are zonal

vegetation in parts of Hungary, Bulgaria, Romania, Moldova, Ukraine and Russia. Detailed local estimations of the border of natural steppe vegetation within these countries were obtained from additional publications (see below).

In addition to zonal steppes, suitable sites for tetraploids also included extrazonal habitats such as open-canopy woodland and thermophilous scrubs (e.g., thermophilous oak forests with patches of open vegetation, particularly on steep slopes), open pine and oak forests on sandy sediments in lowlands, and naturally disturbed sites such as alluvial river sediments, sandy dunes and natural cliffs (e.g., steep slopes with patches of open vegetation on shallow soils). Most importantly, extrazonal habitats include naturally treeless rock outcrops and treeless habitats at high altitudes. These habitats are hereafter referred to as "relict habitats".

The occurrences of tetraploid populations in these relict habitats were particularly interesting to us because *C. stoebe* is a predominantly barochorous species, which limits its uphill dispersal (Mráz *et al.*, 2012b). Consequently, the colonization of such relict sites is unlikely to have occurred recently without the presence of nearby source populations over an extended period. However, some relict sites are under long-term human influence, like past human settlements or medieval castles situated at strategic locations (e.g., on rocky hills like Gellért-hegy in Budapest). In such cases, we carefully considered whether tetraploids in a particular region have been recorded also at other relict sites without obvious human influence.

After defining the historical habitat requirements, we used our total dataset to identify geographical regions where tetraploids regularly occur in natural steppes or relict habitats. We did this assessment across eleven geographical regions, aiming for the most accurate estimation of the native range. This approach is in accordance with previous studies of Lustenhouwer & Parker (2022) and D'Andrea *et al.* (2009) who also investigated patterns in occurrence in a region-focused manner to unravel whether specific regions belong to the native or expanded ranges of *Dittrichia graveolens* and *Lactuca serriola*, respectively. In contrast to such previous efforts, our estimation accounted for the occurrence of a reference taxon. This was crucial in ensuring that the absence of tetraploid records at natural sites did not result from insufficient sampling activity (i.e., addressing sampling biases sensu Lang *et al.*, 2019). In particular, in regions where tetraploids were not found in natural steppes or relict habitats, we examined whether diploids occurred at such natural sites. Because both closely related cytotypes share similar ecological niches (Mráz *et al.*, 2011; Rosche *et al.*, 2018a), it was anticipated that they would have had an equal opportunity to occupy relict habitat types in regions where both cytotypes coexisted over an extended period. In other words, if only diploids were observed in the relict habitats of a particular region, it is unlikely that this region was part of the native range of tetraploids.

Diploids show large-scale presence in suitable natural habitats across their entire sympatric European range, from zonal steppes European Russia in the east to volcanic cliffs in the Massif Central in the west, including numerous relict sites across Central Europe. Given its rather slow natural dispersal and steppic-like ecological requirements, this evolutionarily older cytotype must have colonized its

geographical range a long time ago, likely during the periods of expansions of cold and dry steppes in the Pleistocene (Jamrichová *et al.*, 2017; Divíšek *et al.*, 2022). This means that diploids had enough time to colonize many relict habitats at higher elevations and rock outcrops across Central and Western Europe, making them a suitable reference for assessing sampling activity in relict habitats across the geographical regions. Note that we present some concise considerations of the native range of diploids after evaluating the native and expanded ranges of tetraploids.

In the text below, we describe our assessement for tetraploids across the eleven regions in detail. This native range estimation had more data support in some regions (e.g., Central Europe) compared to others (e.g., Russia), and we highlighted regions where data were scarce. In the text, we used superscripted reference numbers for distinct localities mentioned. These numbers correspond to the consecutive numbers of collected specimens as presented in Note S2: Table N2.2.

3.1 Central Europe without Hungary

Central Europe is here defined as the amalgamation of Germany, Poland, the Czech Republic, Switzerland, Austria and Slovakia. Hungary is excluded from this definition because it was the only Central European country that was suggested to be entirely part of the native range of tetraploids in Ochsmann (2000). Within Central Europe, special attention was given to southern Slovakia and eastern Austria because these regions were also previously assumed to be within the native range of tetraploids (Ochsmann, 2000).

While much of Central Europe was historically covered by forest vegetation, there are also many naturally tree-less habitats in this region. These include sandy dunes located in open sandy pine and oak forests (e.g., in northern Poland and northeastern Germany). In addition, there are numerous relict habitats including rock outcrops and river canyons in the Alps, Hercynian Massifs and Western Carpathians. These habitats are frequently occupied by diploids all across Central Europe [1-21]. In contrast, tetraploid populations are absent on these relict sites indicating that tetraploids arrived in Central Europe recently. Since their arrival, there has not been sufficient time for tetraploids to establish populations in these relict habitats, particularly in terms of uphill dispersal.

Parts of Austria and Slovakia have been previously assumed to be part of the native range, mainly because tetraploids have been recorded there for a long time. However, we believe that the types of habitat occupied are a better indicator of the range status than the age of record within a relatively short span of time (herbarium specimens of *C. stoebe* are available from 1790 in our dataset). For Slovakia, the oldest records of tetraploids were found near the city of Bratislava (Devínská Nová Ves in 1874 [22] and Devín in 1877 [23]). There are several relict rock outcrops in this region. Some of them with diploid populations, but none of them harbor tetraploid populations (Španiel *et al.*, 2008; Mráz *et al.*, 2012b). Additionally, in the Western Carpathians, there are many rock outcrops where only diploids occur (Španiel *et al.*, 2008). This provides evidence that tetraploids have rather recently been introduced to Slovakia.

In Austria, there are also no tetraploid but several diploid populations in relict habitats such as steep slopes and rock outcrops [1-5]. The oldest records of tetraploids in Austria are only a few kilometers away from the above-mentioned Slovak populations (e.g., on the other side of the Danube at Hainburg in 1887 [24]). The close proximity to the oldest records from Slovakia indicates that this region could be an introduction centre for the expansion of tetraploids across Central Europe.

Other early introductions of tetraploids are recorded from Munich (Germany) in 1876 [25] and from Salvan (Switzerland) in 1909 [26]. Natural populations of diploids could be found in the past in Bavaria [6-8] and are generally widely distributed in Germany [9-12]. In Switzerland, there are many natural diploid populations in the Alps [13-15]. For the region of first introduction of tetraploids in Switzerland (Valais), however, an endemic, closely related diploid taxon replaces diploid *C. stoebe*: *C. vallesiaca* (DC.) Jord. This taxon can be found regularly at many natural sites in Valais [27-29]. In the northeastern part of the Central European countries, tetraploids were introduced later: in 1925 in Řepiště (Czech Republic) [30] and in 1965 in Tryńcza (Poland) [31]. In both the Czech Republic [16-18] and Poland [19-21], diploids are widely distributed in natural habitats.

3.2 Hungary

The potential natural vegetation for large parts of Hungary is dominated by woody habitats (Somodi *et al.*, 2017). However, semi-natural dry grasslands are also widely distributed across Hungary (see Molnár *et al.*, 2008; Illyés *et al.*, 2009) for a classification and distribution of these grasslands). These include anthropogenically influenced steppe-like habitats (e.g., Hortobágy), and some grasslands east of the Danube and Tisza rivers (Somodi *et al.*, 2017) where forest steppes are assumed to be potential natural vegetation (Ssymank, 2013). However, we are not aware of any populations of tetraploids there. Also, the semi-natural grasslands across Hungary are predominately colonized by diploids [32-36]. There are some semi-natural grasslands in hill slopes around Pécs and along Dráva River where both diploids [37,38] and tetraploids [39-41] are present. The occurrence of both cytotypes in semi-natural sites in this region was also supported by our samplings [42-45]. While it remains difficult to assess whether these populations are part of the native range of tetraploids, we did not include them because the records from the most natural habitats in the region are diploid [46-48] and there are no tetraploid records from relict habitats in this region. Furthermore, our judgment is in accordance with our considerations in the adjacent regions of Croatia, the northernmost parts of Serbia and the westernmost parts of Romania (see below).

Across Hungary, we also checked for available relict sites provided by rock outcrops. We found that such tetraploid populations can only be found in some natural rock outcrops around and within the city of Budapest (e.g., Sas-hegy, Gellért-hegy) [49-52]. However, Budapest is a very old human settlement with intensive traffics and exchange of goods for a long time and Gellért-hegy was under strong anthropogenic pressure throughout at least the last 2,000 years (Bónis, 1969; Czajlik *et al.*, 2015). Since we are not aware of any other tetraploid population at isolated relict rock outcrops, we consider the entire territory of Hungary as a non-native range of tetraploids.

3.3 Slovenia, Croatia, and Bosnia and Herzegovina

In Slovenia and Croatia, there are many mountains and hills with steep slopes and rock outcrops. However, from these relict places, we recorded only a few diploid populations [53-56], and only one tetraploid population in southernmost Croatia. In particular, this population is located in the exclave Dubrovnik-Neretva County, which is surrounded by Bosnia and Herzegovina (see below). In general, out of the 40 records from Slovenia and Croatia, only four were of tetraploid plants, including only one additional record from a semi-natural habitat in the floodplain of the Drava River [45]. Therefore, we consider the entire territory of Slovenia and Croatia, except for the Dubrovnik-Neretva County in southern Dalmatia, as a non-native range of tetraploids.

The tetraploid population in the Dubrovnik-Neretva County occurs on rock slopes (Carev *et al.*, 2017). Similarly to this site, tetraploids are present in many relict sites across Bosnia and Herzegovina [57-61], and prevail in these habitats over diploids among records from this country in our database. Such relict occurrences of tetraploids were also supported by a recent publication (Pustahija *et al.*, 2013) and our own samplings [62,63]. We therefore consider the entirety of Bosnia and Herzegovina and the Dubrovnik-Neretva County as part of the native range of tetraploids.

3.4 Serbia and Kosovo

Tetraploids are frequent across large parts of Serbia at relict sites such as rock outcrops and narrow river canyons (see below for details). However, in the lowlands of Vojvodina north of the Danube (i.e., towards Hungary), diploids are getting increasingly frequent [64-68]. Here, tetraploids are no longer found on relict sites but only in ruderal [69-71] and some semi-natural habitats [72,73]. In contrast, in the lowlands and low mountain ranges south of the Danube, tetraploids are frequent in semi-natural and relict habitats [74-79] where they are clearly the majority cytotype over diploids.

There are several tetraploid populations in relict places (steep slopes) along the Danube river [80-82]. Our samplings confirmed the occurrence of tetraploids rather than diploids in (semi-)natural and relict sites along the Danube [83] and south of the Danube [84-89]. We therefore draw the northern delineation of the native range of tetraploids in Serbia along the Danube until it reaches the Croatian border. From there our delineation line follows the national border between Serbia and Croatia until reaching Bosnia and Herzegovina (which we consider as part of the native range).

In western Serbia, tetraploids become less frequent toward Montenegro. There are only two (but very old) records from potentially natural sites: one from Drina slopes [77] and another one from the village of Župa near Tutin in southernmost Serbia, situated in a mountainous region [90]. These records indicate that tetraploids may be native here. However, it is difficult to draw a precise range border in this area because sampling activities may have been lower than in other regions and there are many high and remote mountains. Note that there is no diploid population data from this region. We arbitrarily propose the

national border between Serbia and Montenegro until reaching Kosovo, knowing that there are uncertainties where to put the range border in this mountainous region.

It is notable though that the undersampled regions might not necessarily bias the results of our analyses with regards to the native vs. expanded range affiliations of or occurrence data. If a lack of data had resulted in a wrong classification of a certain region as belonging to either the native or expanded range, it also would have meant that this uncertain region was not represented in our statistical analyses due to the lack of data either.

For the mountains in southern and eastern Serbia, there are many records of tetraploid populations (e.g., municipalities of Raška [85-89], Nišava [91-95], Pirot [96-100], and Pčinja [101-105]), suggesting that these parts of Serbia belong to the native range of tetraploids. In Kosovo, tetraploids can be found in relict habitats in the Kopaonik Mts. [106] and Gollak Mts. [107], but there are no records of tetraploids from such sites west and south of these mountains (i.e., towards Albania and North Macedonia) in our occurrence dataset and floristic databases (A. Hajdari, pers. comm.).

Similarly to other Balkan countries, our data for Kosovo are relatively scarce (four occurrence data) which makes our proposed range delineation uncertain. For example, the two abovementioned records from relict places in Kopaonik Mts. [106] and Gollak Mts. [107] originated from our own samplings while we have no herbarium records from these mountains.

3.5 Montenegro, Albania, North Macedonia and Greece

We have no records of *Centaurea stoebe* from Montenegro and Albania. While this may be partly a result of lacking sampling activity, it coincides with the pattern that tetraploids become less frequent in western Serbia and are absent from western Kosovo in our dataset. There are some old records from Albania in the literature (Barina *et al.*, 2018) but the only specimens we found (in herbarium BP) belonged to taxa different from *C. stoebe*. It appears unlikely that tetraploids are or were frequent in Montenegro and Albania and we therefore consider both countries as non-native range of tetraploids.

We also have no firm evidence for tetraploid occurrences from North Macedonia and Greece. We revised 17 specimens from HMMNH, 8 from UPA and another 20 specimens from some other European herbaria (e.g., P, B, SAAR). These specimens were labeled as *C. micranthos*, but they all belonged to taxa different from *C. stoebe*. In other words, it seems unlikely that our lack of data is a signature of lacking sampling activity. Instead, *C. stoebe* seems not to occur (or, if so, only sporadically) in these two countries. Consequently, we excluded them from the native range of tetraploids.

3.6 Bulgaria

Tetraploids can be found frequently at rock outcrops at high altitudes and slopes of the Stara Planina Mts. [107-113], the Vitosha Mt. [114-117] and the Rila Mts. [118-120]. However, west and south of the Rila Mts., tetraploids

are absent and replaced by diploids (e.g., in the Pirin Mts. [121-124] and the Rhodope Mts. [125, 126]) and other taxa (e.g., *C. tartarea* Vel). We do not have evidence for natural sites in the Upper Thracian Plain or south of this plain area either. Previous research (Kuzmanov & Georgieva, 1977; van Loon & van Setten, 1982; Bancheva & Greilhuber, 2006; Mráz *et al.*, 2012a) and our sampling [127-129] confirm the above described patterns in Bulgaria. We, therefore, draw the delineation line from the southern border of Serbia along Kjustendil toward the Struma river, along the Rila Mts. and from there along the border to Upper Thracian Plain until reaching the Black Sea. Similar to other countries in the Balkans (see above), it is difficult to delimit a precise distribution border in this region because there are many high and remote mountains. However, sampling activities have been intermediate (a total of 28 diploid and 49 tetraploid specimens plus 52 specimens that could not be assigned a ploidy level or that did not represent *C. stoebe*), which renders our range estimations in Bulgaria relatively robust.

Climatically determined steppes occur only in the northeast of Bulgaria in Dobruja (Vassilev & Apostolova, 2013) but from this small area we have no records. As for the lowlands north of the Stara Planina Mts., the potential natural vegetation is dominated by thermophilous mixed deciduous forests and forest steppes (Feurdean *et al.*, 2021). This region does not harbor many relict sites. However, there are relict habitats on steep slopes along the Danube from where we have records of tetraploids [130,131]. In general, tetraploids are predominant over diploids (10 records vs. 1 record), including the few relict sites along the Danube. Moreover, Stoyanov & Apostolova-Stoyanova (2024) reported the occurrence of tetraploids in natural habitats of the Rusenski Lom River Valley (Northeast Bulgaria). In this area, tetraploid *C. stoebe* inhabits rocky and dry grasslands dominated by *Chrysopogon gryllus* (S. Stoyanov, pers. comm.). While records of tetraploids from natural habitats north of the Stara Planina Mts. are relatively scarce, we consider the whole region as part of the native range of tetraploids. Additionally, the adjacent area in Romania is part of the native range of tetraploids (see below). In other words, even though tetraploids may have been scattered and scarce across these lowlands in recent times and may be rare in or even absent from Dobruja, we suggest a continuous distribution. It would be difficult to draw a meaningful delineation in these continuous lowlands. Note that if Dobruja would be falsely integrated in the native range of tetraploids, it would not affect our results because the lack of records means that this region is not represented in our statistical analyses (see also above for western Serbia).

3.7 Romania and Moldova

There are many tetraploid populations in rock outcrops and slopes in the Apuşeni Mts. [132-134], the Eastern and Southern Carpathians [135-137] and also on slopes across the low mountains of whole Romania [138-140]. In the Eastern Carpathians, tetraploids become less frequent toward Ukraine. However, in the forelands both to the east and west of the Eastern Carpathians, tetraploids are found in relict habitats [141-143]. Similar to western Serbia, it is difficult to estimate a precise distribution border in the north of the continuous Romanian Carpathians. We therefore consider the whole mountains and low mountains of Romania as

part of the native range of tetraploids. For the distribution in the Carpathians, we propose the Romanian-Ukrainian border as an arbitrary range border to the north.

The lowlands in the west of the Romanian Carpathians toward Hungary and Serbia do not harbor tetraploids in relict habitats. Consistent with the comparable, adjacent areas in Hungary and Serbia, we exclude this region from the native range of tetraploids. Other lowlands include Wallachia, eastern Romania and the entirety of Moldova. In most parts of these regions, thermophilous deciduous forests and forest steppes are the potential natural vegetation (Vassilev & Apostolova, 2013; Feurdean *et al.*, 2021). In these regions, tetraploids are predominant over diploids and occur at many semi-natural dry grasslands [144,145], in open forests [146,147], on river banks and alluvial sediments (e.g., along the Danube [148]), and on slopes of narrow river valleys (e.g., along the Dniester [149,150]). Climatically determined steppes occur only in southeastern Romania (i.e., Dobruja) and southern Moldova (Vassilev & Apostolova, 2013). There are no records of tetraploid populations and only very few records of diploid populations from steppic habitats of this region [151-153]. However, there have been records of *C. stoebe* since the late 19th century (e.g., Brandza & Ştefănescu, 1898), including specifically tetraploids (Tupu & Chifu, 2021). It appears that tetraploids become less frequent toward the climatically determined steppes, but it is difficult to set a clear-cut border. To be consistent with our classification in Bulgaria, we consider the lowlands of southern and eastern Romania and the entirety of Moldova as part of the native range of tetraploids.

3.8 Ukraine

The potential natural vegetation of Ukraine is divided into alpine mountains (i.e., the Ukrainian Carpathians and Crimean Mts.), broadleaf forests in northern and western Ukraine, a large forest steppe belt in the middle of the Ukraine and a large Pontic steppe belt in southern Ukraine (Kajtoch *et al.*, 2016; Semenova & Slizhe, 2020). At high altitudes of the Ukrainian alpine mountains, both diploids and tetraploids are absent. In the broadleaf forests, there are rock outcrops and river slopes that are occupied by diploids [154-158] but not by tetraploids. This pattern coincides with the broadleaf forests in the adjacent countries of Poland, Belarus and Russia where *C. stoebe* populations in natural habitats are exclusively diploid. However, there is one exception for the broadleaf forests in Ukraine, namely the small region southwest of the Carpathians (i.e., the Ciscarpathian and Transcarpathian lowlands in the border region to Romania). Here, the landscape is highly heterogenic, both climatically and floristically (see (Novikov, 2021) for floristic counties in this region). There are some natural populations of tetraploids in the Vygorlat-Gutyn floristic county along the Tysa river [160] and on steep slopes of volcanic low mountains (e.g., at Mt. Chorna near Vynohradiv [161]). We consequently included the low mountains in the Vygorlat-Gutyn floristic county (adjacent to Romania) to the native range of tetraploids but excluded the rest of the broadleaf forests and all alpine mountains from the native range of tetraploids.

In the forest steppe belt, relict populations of tetraploids can be frequently found along river sides and canyons of the Dniester [162,163], Ros [164,165] and Dnieper [166,167] rivers. In this region, tetraploids occur also on steppic slopes [168,169] and hill plateaus [170]. Moreover, literature suggests that both cytotypes occur

on kurgans, i.e., small and remote, man-made hillocks within the agriculturally dominated landscape (Moysiyenko & Sudnik-Wójcikowska, 2010). In general, diploids mostly prevail over tetraploids in the forest steppe belt, except for the Chernivtsi oblast (adjacent to Romania and Moldova) and Kyiv oblast. Interestingly, north of the Chernivtsi oblast, tetraploids become absent in relict habitats whereas diploids are frequently found along the Dniester in this region [171-173]. We therefore consider the forest steppe belt *sensu* Semenova and Slizhe (Semenova & Slizhe, 2020) as part of the native range of tetraploids, except for the regions north and northwest of Chernivtsi oblast.

Within the Pontic steppe belt, both diploids and tetraploids become less frequent with increasing aridity. This pattern coincides with comparable ranges in Dobruja and Moldova (see above). In contrast to the forest steppe belt, there are no records of diploids and tetraploids from kurgans of the Pontic steppe in the literature (Sudnik-Wójcikowska *et al.*, 2011). Shynder (2021) also reported that the *C. stoebe* complex becomes absent southwards in the remnants of Pontic steppes and is there replaced by *C. besseriana* and several local Ukrainian taxa of the *C. arenaria* complex (e.g., *C. odessana*, *C. borysthenica* and *C. savranica*). However, the taxonomic status of these taxa is not yet clarified (Shynder, 2021). It is important to recognize that the vast majority of the Pontic steppes were converted into large agricultural areas several decades ago (Sudnik-Wójcikowska *et al.*, 2011). However, there are various records of tetraploid populations from relict sites on slopes and hill plateaus in the Donbas region [174-177]. This is concordant with the situation in the adjacent regions in southwestern Russia (see below) and shows that tetraploids are capable of existing in the northern Pontic steppes. Note that these localities are slightly less arid than more southern Pontic steppes (e.g., along the coasts of the Black Sea and of the Sea of Azov where *C. stoebe* is absent). In conclusion, the distribution of tetraploids in the Pontic steppes seems to be limited by increasing aridity but, similarly to Dobruja and Moldova, we consider all Pontic steppes in Ukraine as part of the native range of tetraploids. We did so 1) for continuity reasons (difficult to draw a distinct delineation for the continuous Pontic steppes of Ukraine), 2) for phylogenetic reasons (according to the assumed evolutionary cradle of the species group) and 3) for historical habitat suitability reasons (steppes as natural vegetation that have been almost completely transformed to arable land long time ago).

3.9 European part of Russia

The Pontic steppe and forest steppe belts continue in southwestern Russia (Ssymank, 2013). Diploids predominate here in steppic habitats [178-181], but there are also several tetraploid populations from relict habitats, such as steppic or steep river slopes in Belgorodskaya Oblast [182,183], Voronezhskaya Oblast [184], and close to the Don delta (e.g., Nowotscherkassk [185,186]). Tetraploids become generally less frequent east and south of the Don River and are absent from relict habitats, whereas diploids can still be found there (e.g., in steppes south of the Don river [187-189] and along slopes of the Wolga river [190-192]). Diploids also continue to be frequent in steppic and other relict habitats toward the Ural Mts. [193-196]. Similar to Ukraine and the adjacent areas (see above), tetraploids cannot be found in relict habitats north of the forest steppe

belt, whereas diploids are still present [197-200]. We consider tetraploids to be native to the Pontic and forest steppes west and north of the Don River but non-native to the rest of European Russia.

3.10 Rest of Europe

The rest of Europe includes Italy, the Iberian Peninsula, France, the Benelux Union, Great Britain, Scandinavia, the Baltic States and Belarus. These regions are outside the delineation of the native range of tetraploids. We found no tetraploid populations in relict habitats from these regions. There are many diploid populations in relict sites of Italy [201-203], France [204-206] and the Baltic States [207-209], whereas diploids are not present in the other mentioned regions (see below for considerations regarding the native range of diploids). The first introduction of tetraploids in the Benelux Union was recorded in 1882 (Louvain, Belgium [210]). Later followed the colonizations of Scandinavia (Åhus, Sweden in 1908 [211]), Belarus (Rechytsa District in 1919 [212]), France (Paris in 1937 [213]), Italy (Trieste in 1980 [214]) and Great Britain (Leeds in 1999 [215]). In the Iberian Peninsula and the Baltic States, there is no record of tetraploids so far.

3.11 Asia

In the literature, tetraploid *C. stoebe* is often considered a Eurasian species, and more specifically, it is occasionally mentioned that Asia Minor and/or Central Asia may belong to the native range of tetraploids (e.g., Maddox 1979; Hufbauer & Sforza, 2008; Marrs *et al.*, 2008; Rosche *et al.*, 2016). However, we found only one record of tetraploids in Asia, namely from a ruderal site in Turkey (Eregli in 1958 [216]). In contrast, there are many diploid specimens across the continuous steppe and forest steppe belts in Kazakhstan and Asian Russia [217-223].

3.12 Considerations on the native range of diploid *Centaurea stoebe* subsp. *stoebe*

To estimate the native range of diploids, we applied the same criteria as done for tetraploids. Overall, we consider diploids as native across the sympatric ranges of both cytotypes in Europe (Fig. S5 for a map). However, for the Asian distribution, our data are scarce, which does not allow a clear definition of the diploid range there. Particularly from Far East Asia, we have only a few records [224-227].

In Europe, we found that, wherever diploids occur across the above mentioned regions, they inhabit natural habitat types, including relict sites. We assume that diploids spread over its current distributional range at the latest during the period of cold steppes of the last glacial period, together with many other Eurasian steppe species (Kuneš *et al.*, 2015; Jamrichová *et al.*, 2017; Divíšek *et al.*, 2022). At this time, most of the European landscape was open with strong winds from the east (Pokorný *et al.*, 2015; Chytrý *et al.*, 2022). Together with epizoochorous dispersal, the wind may have facilitated the spread of diploids, apparently even at tall rocks. When most European landscapes were later covered by forest vegetation, diploids – like many other species with steppic niches – may have been restricted to

relict, extrazonal habitats such as rock outcrops and open-canopy vegetation at high altitudes (Kirschner *et al.*, 2020).

Since the Neolithic period, the anthropogenic activities have transformed the European landscape by the means of large-scale urbanization, dramatic changes in land uses, including a massive deforestation of Europe (Kaplan *et al.*, 2009). This led to the establishment of many semi-natural grasslands all across Europe (Hejcman *et al.*, 2013), likely with a frequent establishment of diploid populations in these grasslands. Increasing animal herding may have further assisted dispersal to grasslands and to rock outcrops across the distribution of diploids (Wessels-de Wit & Schwabe, 2010). From natural and semi-natural habitats, diploids have also been regularly introduced to ruderal habitats [228-232]. However, diploid populations in ruderal habitats have a high probability of becoming extinct within a few generations whereas tetraploids appear to be better adapted to persist in highly disturbed environments due to their polycarpic life cycle and reduced negative impacts of founder effects (Rosche *et al.*, 2016; 2017; 2018a). There is indeed no indication of any range expansion of diploids in the last two centuries in Europe. This pattern suggests that diploids have a low ability to expand their range under the current environmental settings in the anthropogenic era. Our estimation of a low expansion potential of diploids coincides with the prominent geo-cytotype distribution pattern, that is, diploids are still the majority cytotype in Eurasia but have never been recorded in North America so far (Treier *et al.*, 2009; Mráz *et al.*, 2011).

In some marginal areas of their distribution, diploids may slightly decline, such as at the northwestern edges toward Atlantic climate (e.g., Ochsmann, 2000; Welss *et al.*, 2008; Romahn, 2021). For example, we recorded only one record each from Denmark (1910) [233], Norway (1914) [234], England (1917) [235] and Sweden (1999) [236]. Here, the abandonment of traditional land use forms reduces the abundance of many semi-natural grassland species (e.g., Rosche *et al.*, 2018b; 2022). However, these declines concern only a small proportion of diploid populations whereas their overall distribution appears stable over time. In other words, the expansion of tetraploids does not appear to come at the expense of diploids. This assumption is supported by the fact that mixed-ploidy populations that had been revisited in time series showed rather constant proportions of tetraploids to diploids over time (Mráz *et al.*, 2022). This co-existence may be promoted by the reproductive isolation of both cytotypes due to the triploid block (Mráz *et al.*, 2012b).

New Phytologist

Supplementary Table N2.2. References of herbarium specimens and flow cytometrical records that are supporting the estimation of the native range of tetraploids (i.e., as referred in the text above).

Number	Herbarium/FCM	Reference code
1	GJO	0084373
2	GZU	17*
3	GZU	000271609
4	B	10 0141761
5	B	10 1068138
6	H	C.547094
7	P	02815749
8	STU	13*
9	HBG	528717
10	AMD	34844
11	POLL	Fl. Mainz Rheinhessen no 03/245
12	DR	016831
13	GLM	53*
14	Z	1*
15	BASBG	00029710
16	WA	0000051229
17	PRC	232655
18	BRNM	2259736
19	KRAM	114724
20	BNL	Bot-ZfB-BNL–001663
21	BRNU	574839
22	BRNU	123016
23	BRNU	123019
24	W	cited in Ochsmann (2000): no 65
25	M	cited in Ochsmann (2000): no 9
26	G	cited in Ochsmann (2000): no 124
27	BASBG	00030073
28	UPS	V-707740
29	UPS	V-707737
30	PRC	432*
31	KRAM	011299
32	SIB	115613
33	BRNM	0480628
34	BRNM	0530843
35	BRNM	0480628
36	SAMU	4*
37	BRNM	0480628
38	JPU	14*
39	JPU	4*
40	JPU	5*
41	JPU	1*
42	FCM	Nagy, D. (46.086, 18.238)
43	FCM	Nagy, D. (45.884, 18.219)
44	FCM	Nagy, D. (46.027, 18.470)

45	FCM	Purger, D. (46.097, 17.222)
46	BUCA	29662
47	BUCA	122628
48	BUCA	122640
49	GFW	14525
50	BP	cited in Ochsmann (2000): no 157
51	BP	cited in Ochsmann (2000): no 173
52	P	02814977
53	LJU	10012985
54	LJU	63829
55	LJU	10060856
56	FCM	Purger, D. (45.472, 17.641)
57	H	C.403276
58	SARA	48735
59	SARA	48736
60	SARA	44439
61	SARA	44441
62	FCM	Kožić, K. (44.232, 18.439)
63	FCM	Kožić, K. (43.783, 19.302)
64	BUNS	24455
65	BUNS	24468
66	BUNS	24479
67	LJU	10073837
68	PR	62882
69	BUNS	24466
70	FR	0119021
71	FR	0119020
72	BUNS	24467
73	BUNS	24451
74	BUNS	24499
75	BUNS	24550
76	BUNS	24491
77	P	02815731
78	BUNS	24497
79	BUNS	24551
80	PR	62882
81	LY	cited in Ochsmann (2000): no 51
82	BUNS	24500
83	FCM	Mráz, P. & Mrázová, V. (45.141, 20.254)
84	FCM	Purger, D. (45.178, 19.886)
85	FCM	Mráz, P. & Mrázová, V. (43.311, 20.866)
86	FCM	Mráz, P. & Mrázová, V. (43.411, 20.831)
87	FCM	Kožić, K. (43.313, 20.852)
88	FCM	Kolář, F. (43.306, 20.900)
89	FCM	Mráz, P. & Mrázová, V. (43.306, 20.868)
90	BEO	10637
91	B	10 1068253

92	NIS	6808
93	JE	cited in Ochsmann (2000): no 47
94	PR	380*
95	M	cited in Ochsmann (2000): no 46
96	UPS	V-711022
97	US	NMNH1131821
98	NIS	7937
99	NIS	9186
100	GZU	000070087
101	NIS	7437
102	B	10 1068391
103	BEO	10639
104	B	10 1068267
105	B	10 1068250
106	FCM	Hajdari, A. (42.673, 21.214)
107	FCM	Hajdari, A. (42.914, 20.901)
108	SO	78820
109	BRNU	237624
110	KRAM	432733
111	B	10 1068177
112	HAL	87232
113	HAL	87233
114	KRAM	326570
115	HAL	27044
116	M	cited in Ochsmann (2000): no 5
117	SOM	86053
118	H	C.403252
119	BRNM	443383
120	H	C.403255
121	BRNM	0517325
122	SOM	86217
123	BRNM	443382
124	BRNM	00517321
125	W	cited in Ochsmann (2000): no 214
126	GB	7*
127	FCM	Mráz, P. & Mrázová, V. (42.115, 23.320)
128	FCM	Mráz, P. & Mrázová, V. (43.592, 22.859)
129	FCM	Bancheva, S. (42.251, 23.803)
130	KRAM	432733
131	KRAM	416495
132	FCM	Mráz, P. & Mrázová, V. (46.427, 23.455)
133	CHER	13*
134	GB	25*
135	WU	cited in Ochsmann (2000): no 88
136	IASI	55323
137	CHER	59*
138	BRNU	DA22/002

139	BRNU	DA22/025
140	IASI	73295
141	CL	199213
142	CHER	32*
143	FCM	Mráz, P. & Mrázová, V. (47.401, 23.509)
144	IASI	81088
145	M	cited in Ochsmann (2000): no 54
146	IASI	36445
147	LE	33*
148	IASI	71041
149	KW	48*
150	CHIS	112488
151	CL	127195
152	IASI	71041
153	CHIS	104416
154	KW	512*
155	KW	223*
156	KRAM	115102
157	KW	096256
158	MW	0458971
160	LWS	96303
160	KWHA	25*
161	YALT	29*
162	CHER	85*
163	LWKS	6662
164	KWU	40295
165	KWU	49957
166	KWHU	63*
167	KWU	40332
168	CHER	78*
169	KW	558*
170	KWU	49965
171	CWU	19*
172	LWS	95342
173	KRAM	499008
174	DNZ	27*
175	DNZ	57*
176	KW	140*
177	LE	28*
178	MW	0458842
179	MW	0458831
180	MW	0444714
181	GOET	cited in Ochsmann (2000): no 120
182	L	3035941
183	MW	0458840
184	MW	0458841
185	YALT	50*

186	MW	0458838
187	MW	0633862
188	MW	0633886
189	MW	0633878
190	P	02814968
191	KAZ	51*
192	KAZ	55*
193	BRNU	591297
194	KW	23*
195	UFA	310
196	MW	0444753
197	MW	0444648
198	MW	0444650
199	MW	0444744
200	NS	0019403
201	UPS	V-710836
202	L	3035921
203	B	10 1068156
204	L	3035934
205	NCY	46544
206	L	3035944
207	TAM	0018727
208	H	1447199
209	BILAS	41124
210	BR	0000010749562
211	O	V-578996
212	YALT	42*
213	P	04308884
214	M	cited in Ochsmann (2000): no 38
215	PRC	422*
216	STU	14*
217	MW	0150204
218	KUZ	007630
219	KUZ	007625
220	KUZ	007635
221	KUZ	007638
222	IRK	4081
223	KUZ	007629
224	VLA	VLA398941
225	VLA	VLA291808
226	VLA	VLA281740
227	MW	0954542
228	GJO	80410
229	BRNM	74066
230	B	10 1068246
231	O	V2266550
232	TAM	0065278

233	CL	446520
234	O	V-578992
235	OXF	1*
236	FCM	Lövkvist & Hultgård (1999)

Note: The column Herbarium/FCM indicates whether the reference is a herbarium specimen (if so, the herbarium acronym is given; see Table S2 for the acronyms) or a flow cytometry sample (if so, indicated by “FCM”). The reference codes are herbarium sheet numbers or codes under which the specimens are referred to in our database (Dataset 1). These can be barcodes or similar codes of the respective herbaria. Numbers with asterisks indicate that we could not find a suitable reference code on the specimen labels. In those cases, we gave them a consecutive number under which we stored the scans in our database. Moreover, some specimens have been cited in Ochsmann (2000). For these records, we present the consecutive number under which they were listed in the Appendix of this publication (i.e., “Appendix B”). For the flow cytometry samples, the collector and the geographic coordinates are given. Details for these records can be found in Table S4 and in Dataset 1. Note that the category flow cytometric data also includes one chromosome count (the last record in this table) which was published by Lövkvist & Hultgård (1999).

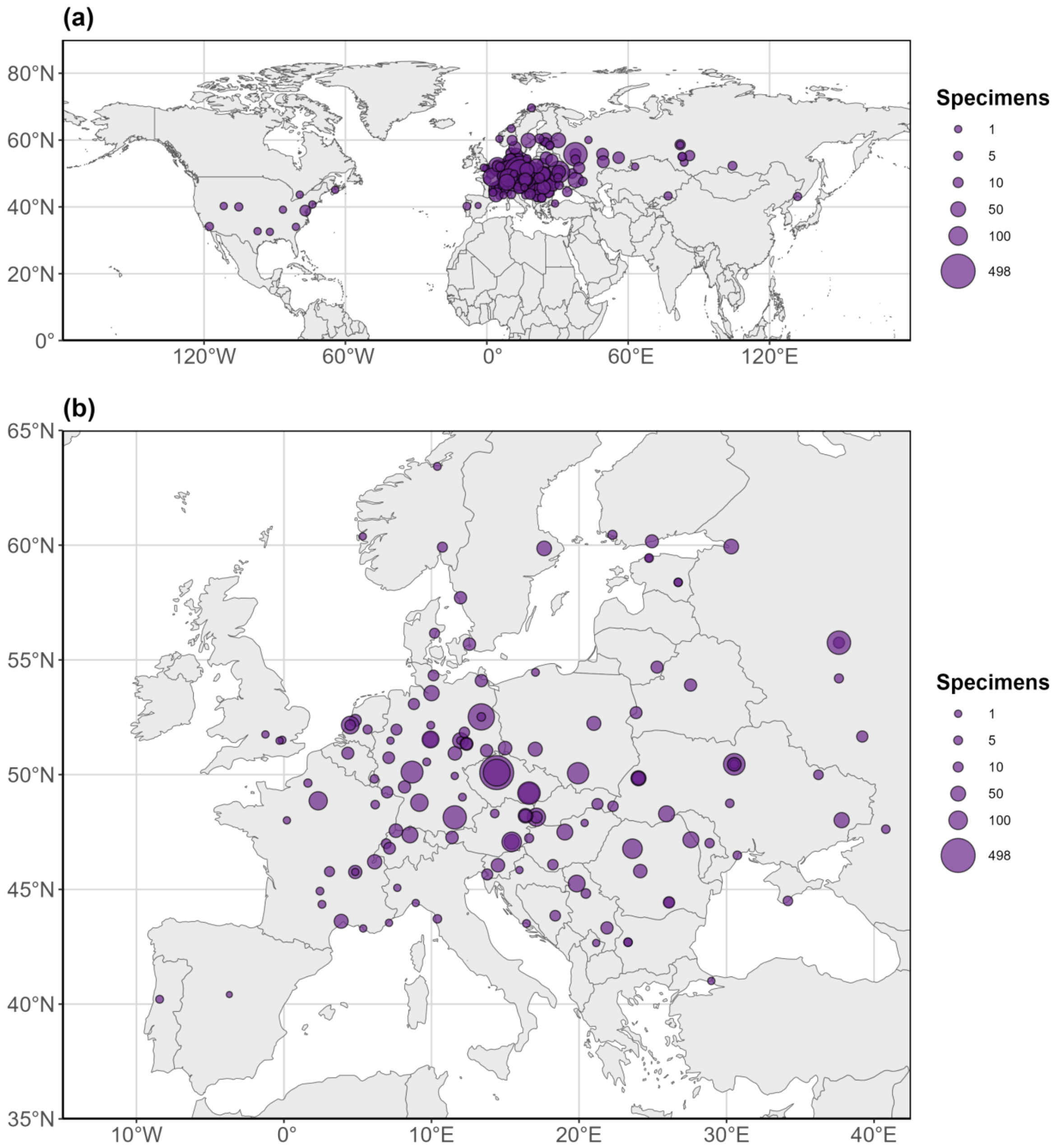

Supplementary Fig. S1. Geographical distribution of the 167 investigated herbaria with dot area corresponding to the number of revised *Centaurea stoebe* specimens (see legend, squished scale). The maps display (a) all herbaria from the Northern Hemisphere and (b) the European herbaria. Note that in some cities more than one herbarium was studied (overlapping dots). In the North American herbaria, we only considered European specimens. A list of all herbaria, their acronyms and the number of recorded specimens are given in Table S2. The herbaria AMD, U and WAG have been recently moved to L, and similarly, BNL to SAAR, and OCHS to HAL. For the sake of clarity, we mapped these herbaria according to their former location (i.e., where they were located during the main sampling activity of their *Centaurea stoebe* collections).

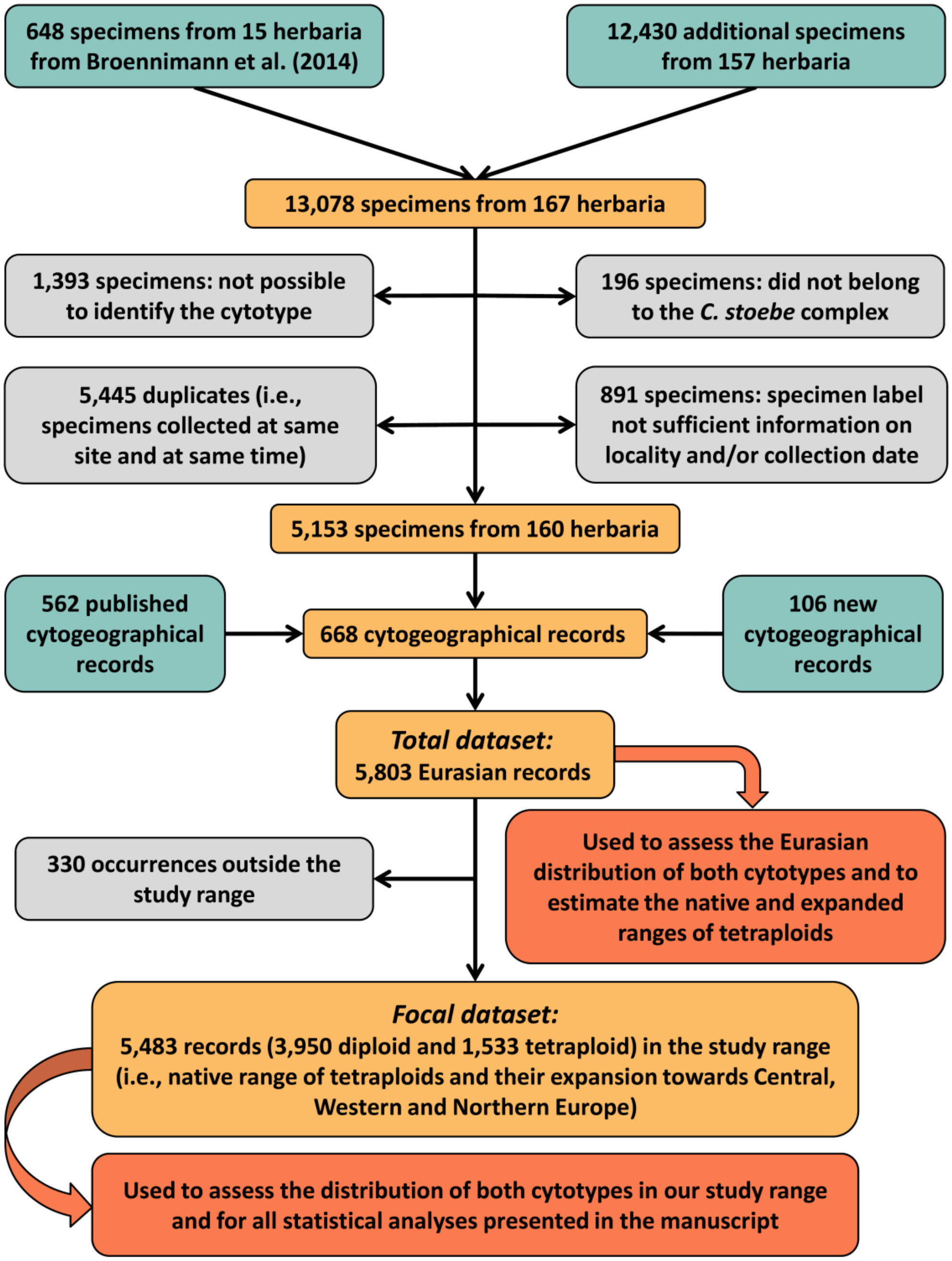

Supplementary Fig. S2. Flowchart on creating our dataset. Turquoise boxes indicate the sources of input data. We started with revising a dataset from Broennimann *et al.* (2014) including 648 European specimens of the *Centaurea stoebe* complex from 15 herbaria. Furthermore, we revised additional 12,430 herbarium specimens from 167 herbaria. These herbaria included five of the 15 herbaria studied in Broennimann *et al.* (2014) which we revisited to complete the dataset (see Table S2 for details). Gray boxes represent steps when we removed data. All Asian occurrences and the occurrences from the expanded range in European Russia were excluded (i.e., 338 occurrences “outside the study range”) as we focused only on the native range of tetraploids and their expansion toward Northern, Western and Central Europe in our analyses (i.e., focal dataset). The yellow boxes summarize the datasets after adding or removing data. The orange boxes show which datasets were used for particular analyses. Note that the number of records of the total dataset (5,803) differs from the sum of 5,153 herbarium and 668 cytogeographical data (which would be 5,821), as some occurrence data have been recorded in both herbarium specimens and flow cytometric data.

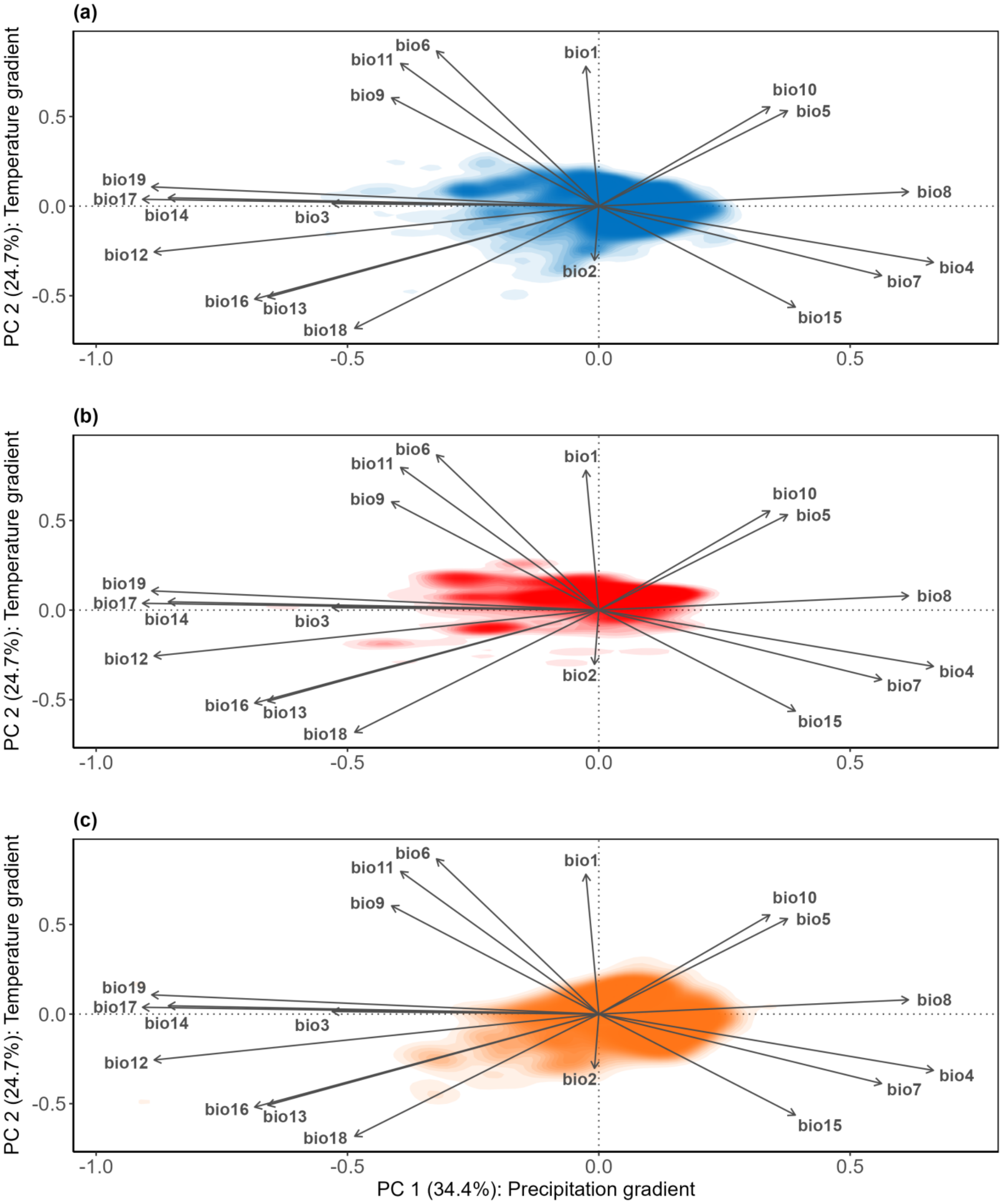

Supplementary Fig. S3. Climatic niche differentiation of diploid and tetraploid *Centaurea stoebe* across the study range in Europe. One PCA was performed with all data while the cytotypes in native and expanded ranges have been plotted separately for the sake of clarity. In particular, the panels show smoothed kernel densities with shading proportional to the occurrence densities of (a) diploids across the entire study range (blue), (b) tetraploids across their expanded range (red), and (c) tetraploids across their native range (orange). Variances explained by the principal components are displayed in parentheses. The plots are presented as biplots including information on the PC scores of the bioclimatic variables as indicated by arrows. Meaning of the bioclimatic variables can be found at https://www.worldclim.org.

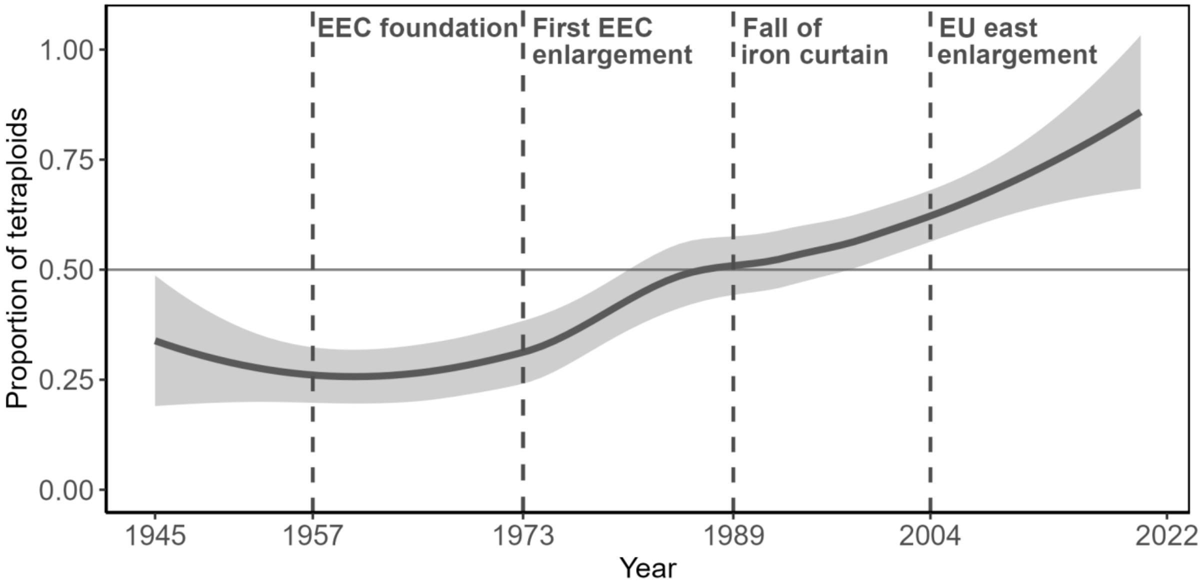

Supplementary Fig. S4. Predicted proportion of tetraploid relative to all *Centaurea stoebe* records in the ruderal habitats of the expanded range of tetraploids since 1945. 1945 was chosen as it marks the onset of the Anthropocene (sensu Zalasiewicz *et al.*, 2015). The line shows the model prediction of the generalized additive logistic model (indicated as solid line referring to a significant relationship; n = 1,262). The band shows the confidence interval (1σ uncertainty). The thin horizontal line indicates a proportion of tetraploids of 50%. We contextualized the temporal patterns with political changes in Europe that influenced travel and trade between and within the native and expanded ranges (see vertical dashed lines). The proportion of tetraploids increased in the late 1950s which coincides with the foundation of the EEC and its first enlargement. This increase flattened in the 1980s. Since 1989, corresponding with the fall of the iron curtain, there was another, exponential increase of the proportion of tetraploids, persisting until the present. Abbreviations: EEC, European Economic Community; EU, European Union.

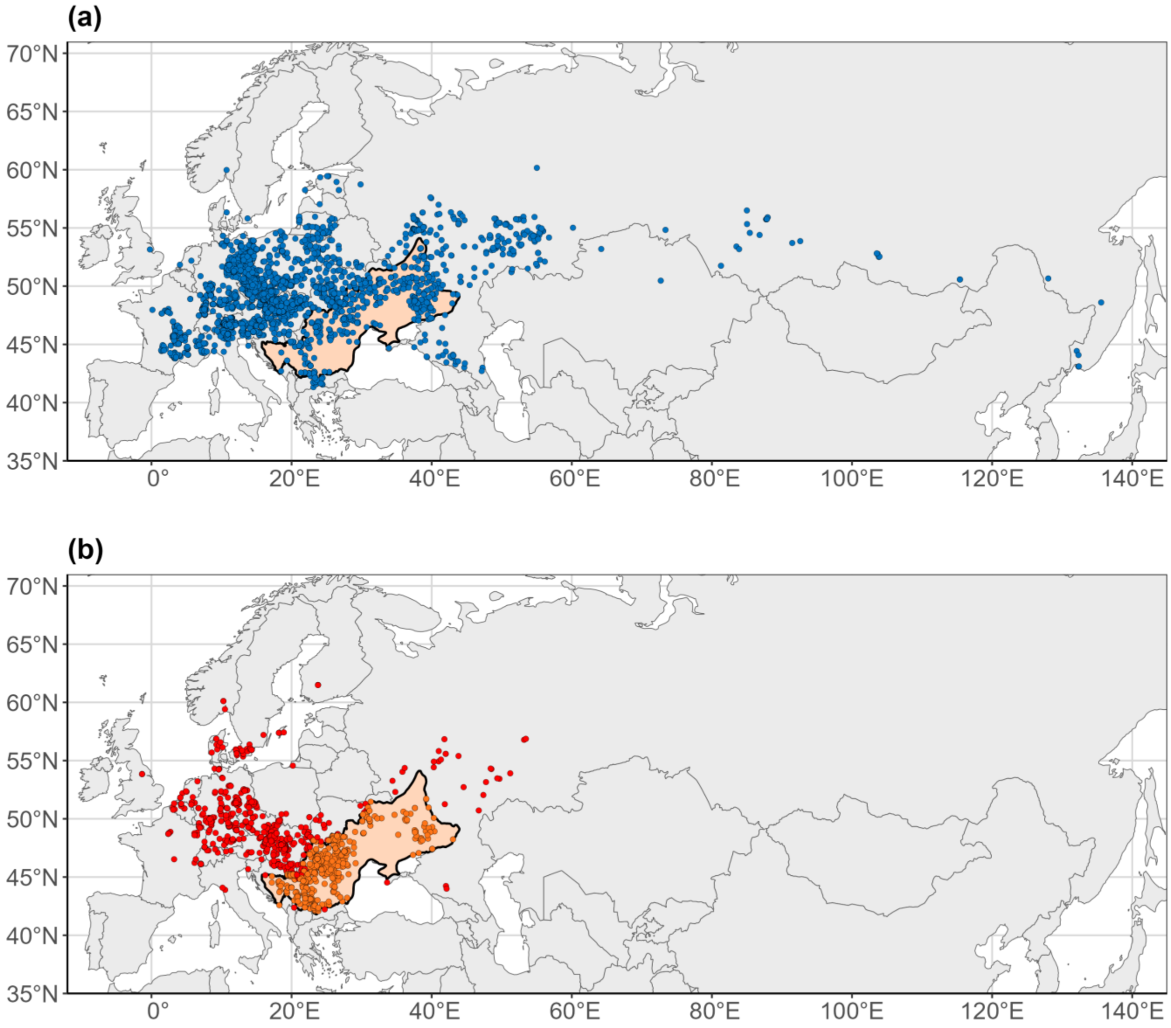

Supplementary Fig. S5. Geographical distribution of all *Centaurea stoebe* records. The maps show the Eurasian distribution of (a) diploids (blue dots) and (b) tetraploids. In tetraploids, occurrences in the native (orange dots) and expanded ranges (red dots) are distinguished. The light orange-colored area represents the estimated native range of tetraploids. Occurrences are plotted chronologically with newer occurrences overlapping older ones. For diploids, the entire European distribution is considered to be their native range. Regarding the Asian distribution, we would need more records from there to adequately quantify the range dynamics of diploids in Asia.

(a)

(b)

(c)

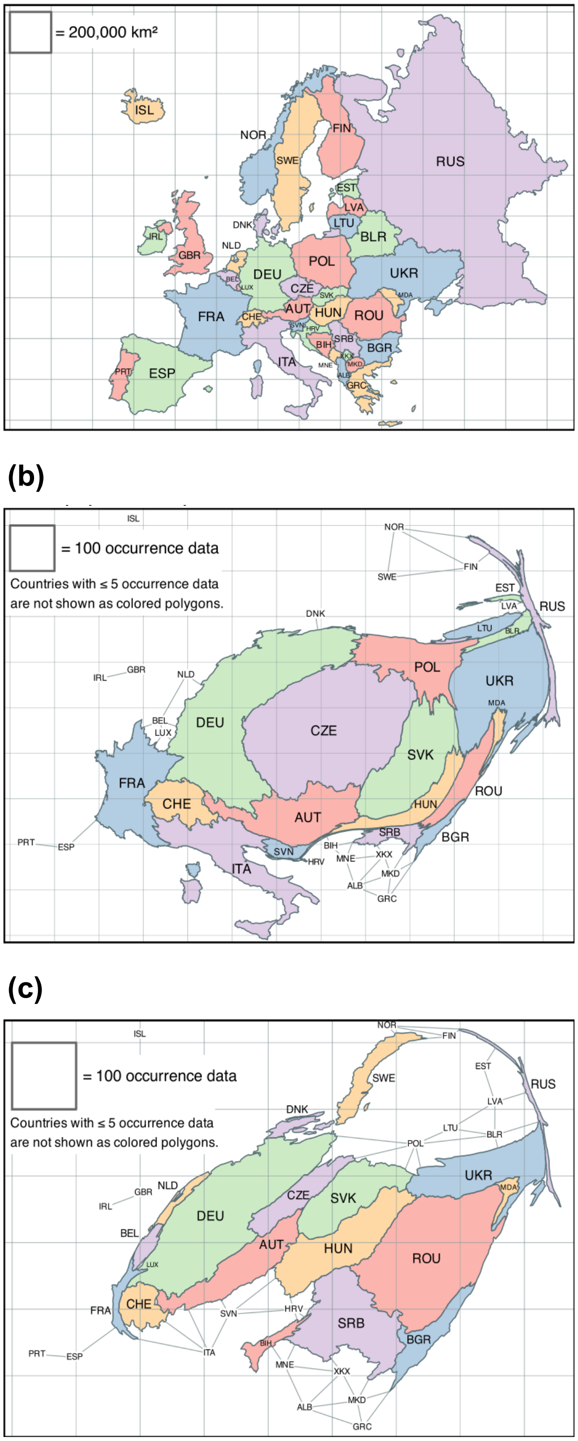

Supplementary Fig. S6. Cartograms illustrating the spatial distribution of diploid and tetraploid *Centaurea stoebe* at the country level across Europe. The areas of European countries are plotted proportionally to (a) their real geographical area, (b) the number of records of diploid populations and (c) the number of records of tetraploid populations. Legends in the top-left corner of the panels indicate the conversion between area and the number represented. For the sake of clarity, Andorra, Cyprus, Liechtenstein, Malta, Monaco, San Marino and the Vatican have been removed due to their small sizes. Countries with five or fewer records are labeled but not shown as colored polygons. The cartograms were created with the method by Gastner *et al.* (2018).

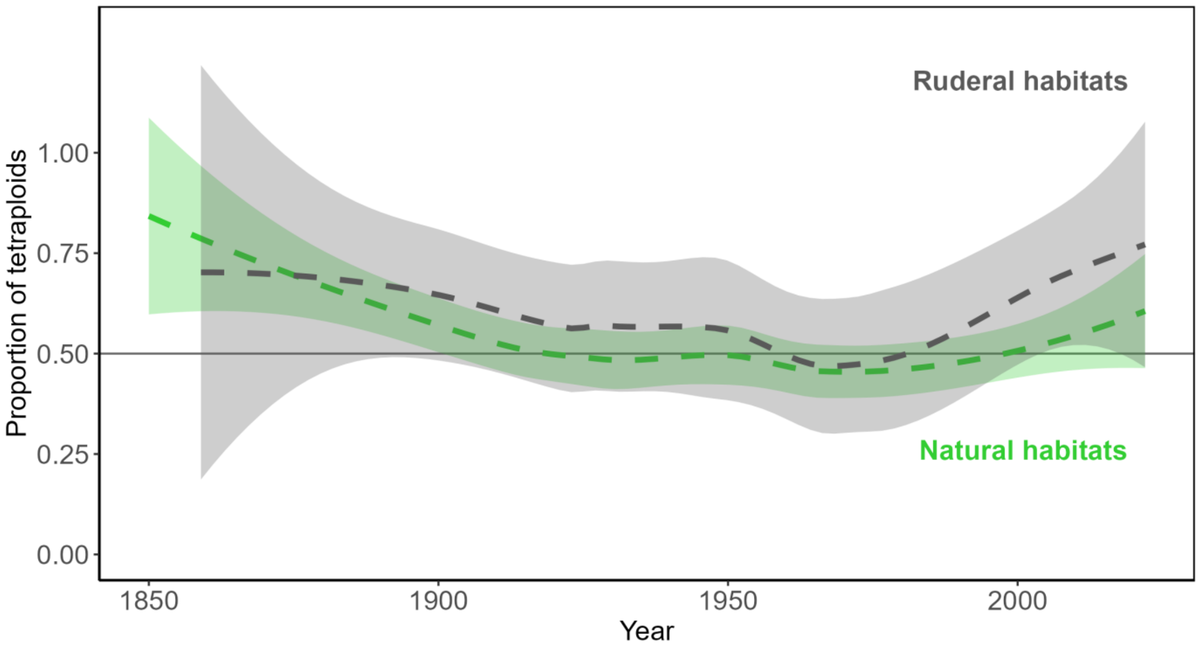

Supplementary Fig. S7. Predicted proportion of tetraploid relative to all *Centaurea stoebe* records over time within the native range of tetraploids. The proportion of tetraploids is shown for ruderal (gray) and natural (green) habitats. Model predictions are depicted using generalized additive logistic models, with dashed lines denoting non-significant relationships (n = 1,091). The shaded bands show confidence intervals (1σ uncertainty). Thin gray line indicates a proportion of tetraploids of 50%. Geographical distribution of ruderal populations and natural populations can be found in Fig. S8.

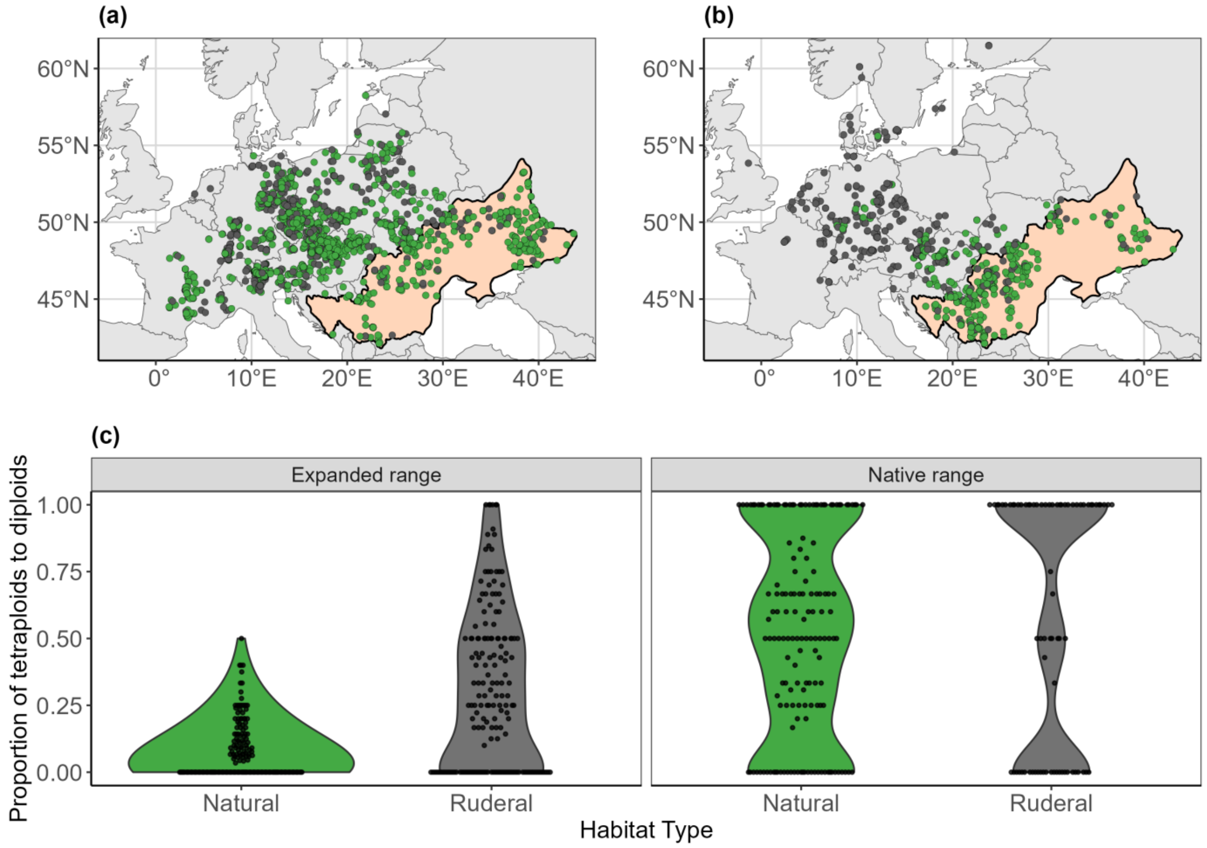

Supplementary Fig. S8. Habitat preferences of diploid and tetraploid *Centaurea stoebe* across the study range. Geographical distribution of ruderal populations (gray dots) and natural populations (green dots) is shown for (a) diploids and (b) tetraploids. The light orange-colored area represents the estimated native range of tetraploids. Occurrences are plotted chronologically with newer occurrences overlapping older ones. (c) Proportion of tetraploid relative to all *C. stoebe* records in both habitat types across the native and the expanded ranges of tetraploids. (a) and (b) show that habitat preferences differed strongly between cytotypes: Tetraploids occurred mostly in ruderal habitats (72%) and less frequently in natural habitats (28%). Diploids showed opposite preferences, with 71% occurrences in natural habitats and 29% occurrences in ruderal habitats. Panel (c) shows that this difference in habitat preference was much stronger pronounced in the expanded than in the native ranges. Plotted in (c) are annual proportion data in the respective habitat type × range combinations in violin plots (horizontal lines include first and third quantiles and the median which sometimes overlap because many annual proportion records were 0 and 1). Note that the different patterns in the proportion of tetraploids should be interpreted with caution. The proportion values may be partially affected by our native range estimation which based, among other considerations, on the current distribution of diploids and tetraploids in natural steppes and relict habitats (these habitats represent 15.2% of what we counted in total for natural habitats). This approach may consequently result, to some extent, in circular reasoning when testing differences between habitat types in native and expanded ranges. Thus, we refrained from statistically analyzing this pattern and rather present the data exploratively. It is important to note, however, that our native range estimation was based on *current* distribution data which thus does not affect the spatio-temporal shifts in the predicted proportion of tetraploids that we are mainly interested in (i.e., slopes as a function of time in Fig. 2 of the main manuscript).

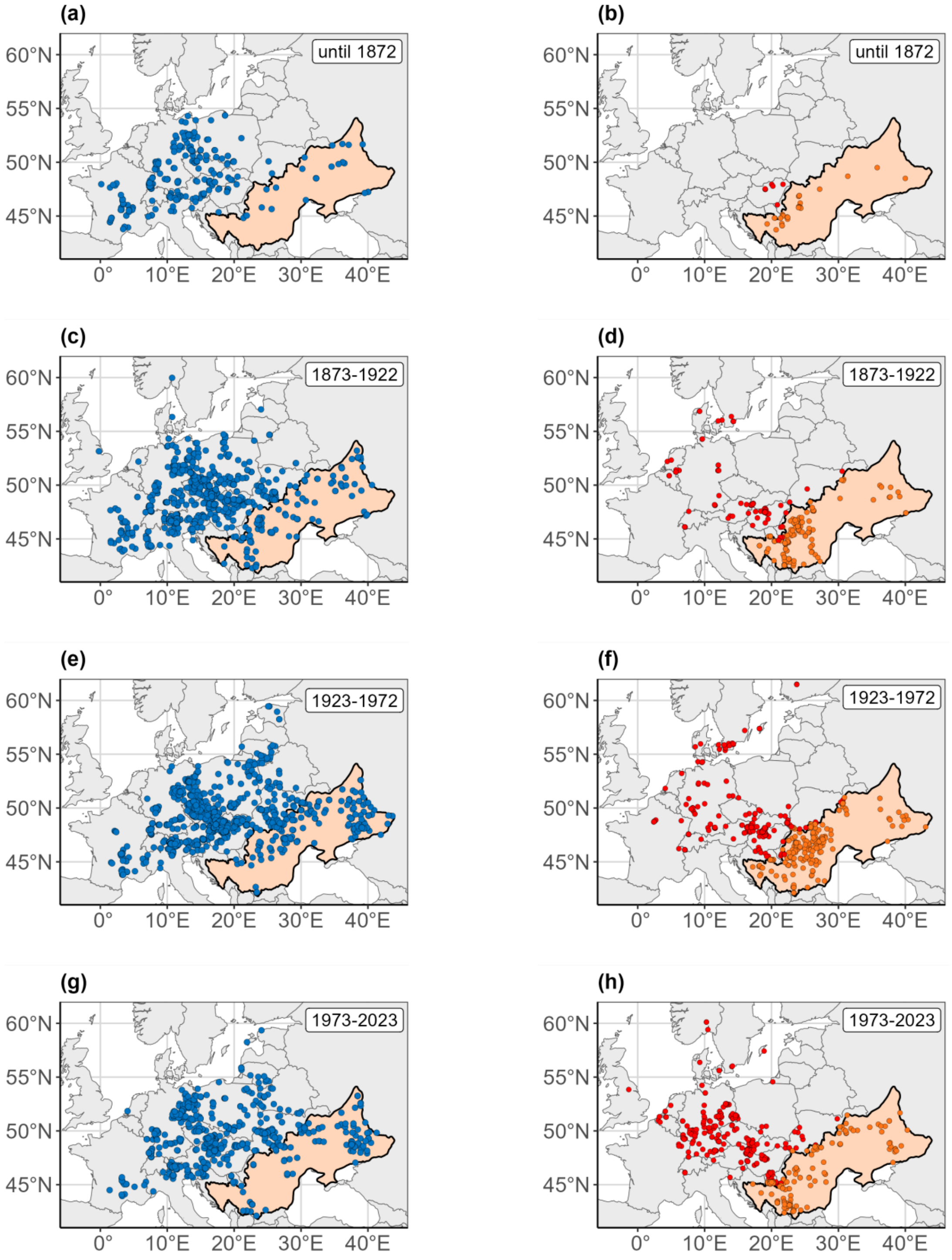

Supplementary Fig. S9. Range dynamics of diploid (blue dots) and tetraploid *Centaurea stoebe* across 50-year time intervals in our study range. In tetraploids, occurrences in the native (orange dots) and expanded ranges (red dots) are distinguished. The light orange-colored area represents the estimated native range of tetraploids. Occurrences are plotted chronologically with newer occurrences overlapping older ones. Panels show (a) diploid records until 1872, (b) tetraploid records until 1872, (c) diploid records between 1873 and 1922, (d) tetraploid records between 1873 and 1922, (e) diploid records between 1923 and 1972, (f) tetraploid records between 1923 and 1972, (g) diploid records between 1973 and 2023 and (h) tetraploid records between 1973 and 2023. The 50-year time intervals were chosen arbitrarily, starting in 1872 because collection activities were low before 1870.

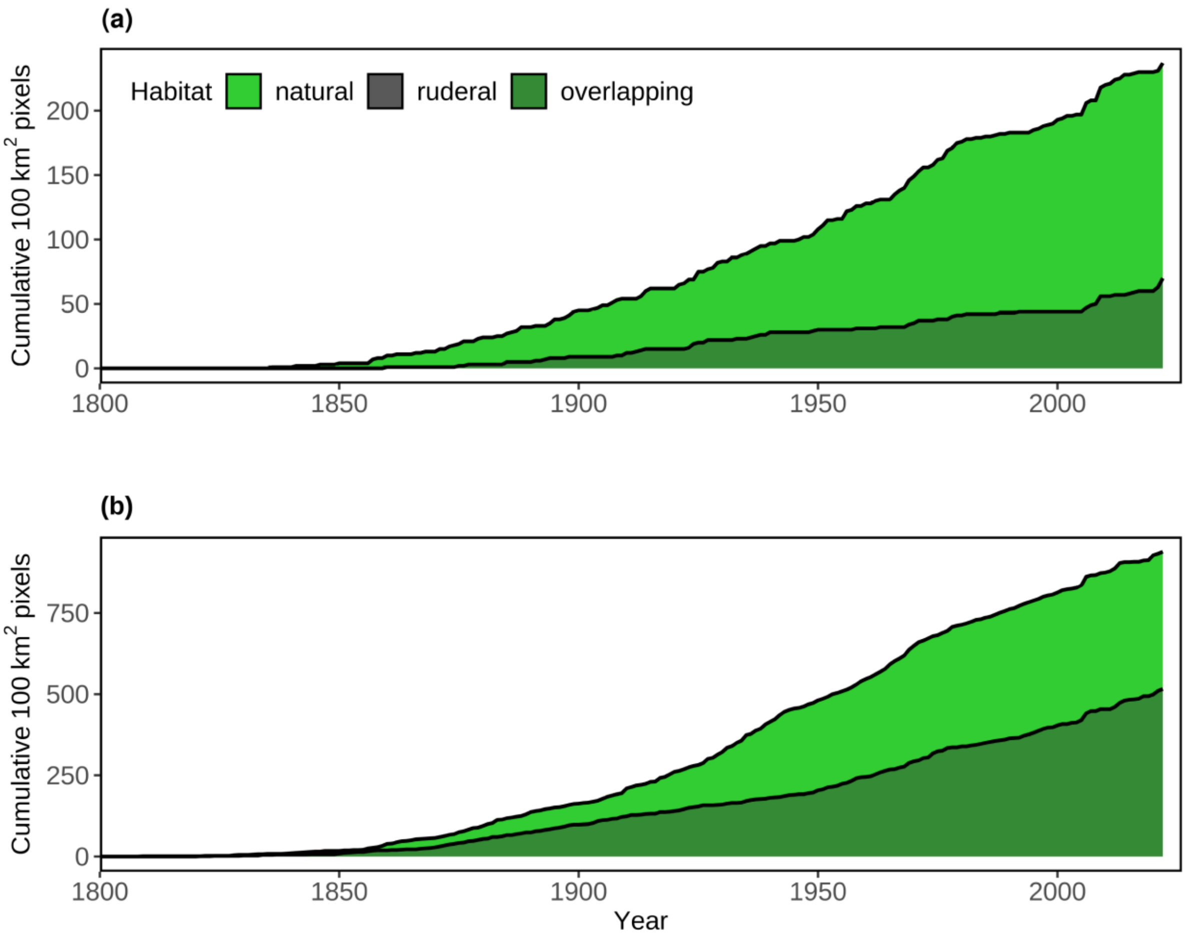

Supplementary Fig. S10. Range size over time of diploid and tetraploid *Centaurea stoebe* in their native ranges, distinguished between ruderal (gray) and natural (light green) habitats. The habitat-specific data are visualized as overlapping (dark green), not stacked, areas. (a) Range size over time of tetraploids across their native range. (b) Range size over time of diploids across the expanded range of tetraploids. Range size dynamics were estimated as the cumulative number of occupied 10 km × 10 km pixels over time (i.e., counting the first records in distinct pixels). Because diploids are native to the expanded range of tetraploids, both scenarios represent range size dynamics in native ranges (i.e., data accumulation patterns of herbarium collections caused by general collection activities). These two scenarios are thus opposed to the patterns of the range size dynamics of tetraploids in their expanded ranges (Fig. 3 in the main manuscript).

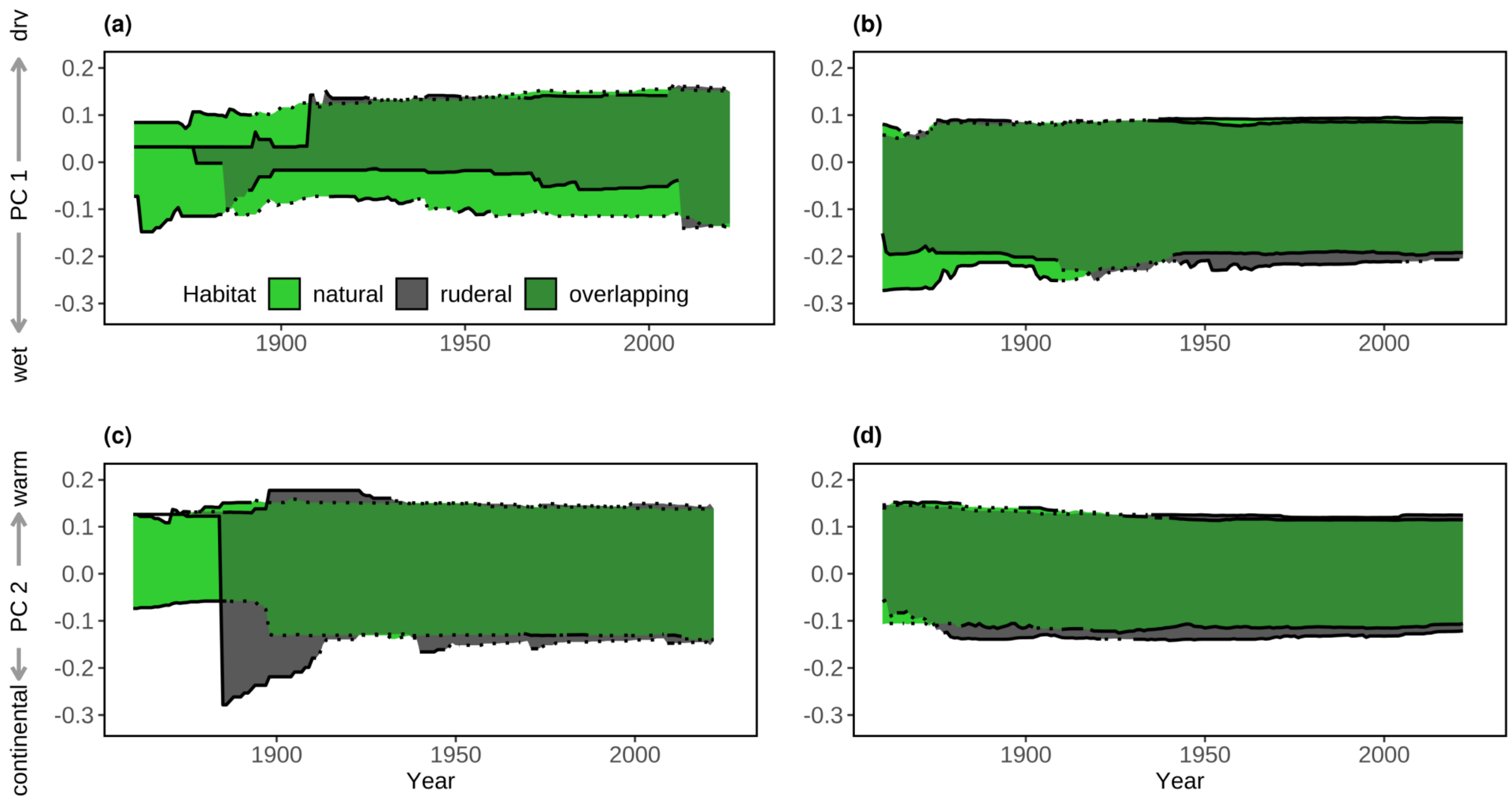

Supplementary Fig. S11. Realized climatic niche breadths over time of diploid and tetraploid *Centaurea stoebe* in their native ranges. The precipitation niche over time is shown for (a) tetraploids across their native range and for (b) diploids across the expanded range of tetraploids. The precipitation gradient ranges from high to low precipitation (first axis of the PCA, see Fig. S3). The temperature niche over time is similarly shown for (c) tetraploids across their native range and (d) diploids across the expanded range of tetraploids. The temperature gradient ranges from continental toward warmer climate (second PCA axis). In each panel, the temporal dynamics of the niches are distinguished between ruderal (gray) and natural (light green) habitats (overlapping areas in dark green). Lines indicate niche limits, with solid and dashed lines denoting significant and non-significant differences between populations from natural and ruderal habitats, respectively. Because diploids are native to the expanded range of tetraploids, all four panels (i.e., two scenarios) represent niche dynamics in native ranges. These two scenarios are thus opposed to the niche dynamics patterns of tetraploids in their expanded range (Fig. 3 in the main manuscript).

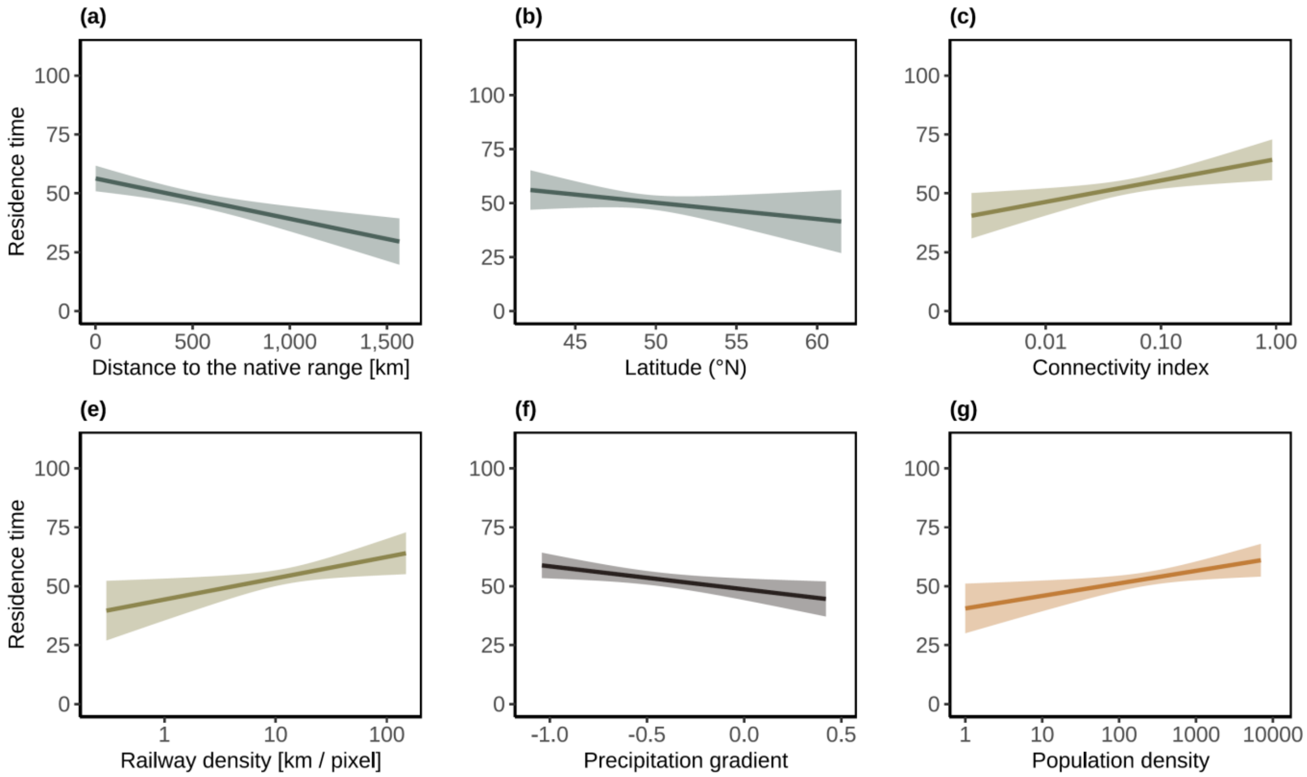

Supplementary Fig. S12. Linear predictors of the initial spread of tetraploid *Centaurea stoebe* across its expanded range. As a proxy for the initial spread, we used the residence time (i.e., the time since each of distinct 10 km × 10 km pixels had been colonized by tetraploids). As predictor variables, three spatial variables (dark green), three climatic variables (black), three variables related to dispersal corridors (olive) and three variables related to urbanization (orange) were tested in a boosted regression tree (BRT) for their effect on the initial spread of tetraploids in their expanded range (Fig. 5a in the main manuscript). The dispersal corridor and urbanization data were data extracted from 10 km × 10 km pixels from the distinct collection year, i.e., spatio-temporally explicit data. From the 12 explanatory variables, we plot the linear relationships of those predictors that had a significant effect on the residence time in the BRT in order of the relative importance of each predictor in the BRT. The predictor variables included (a) distance to the native range, (b) latitude, (c) connectivity index, (d) longitude, (e) railway density, (f) precipitation gradient, and (g) population density. Note that BRTs predict piecewise functions – not a constant slope – over the entire data range. For the sake of clarity, however, we here plot constant linear slopes. The connectivity index, railway density and population density data are $\log_e$-scaled for plotting.

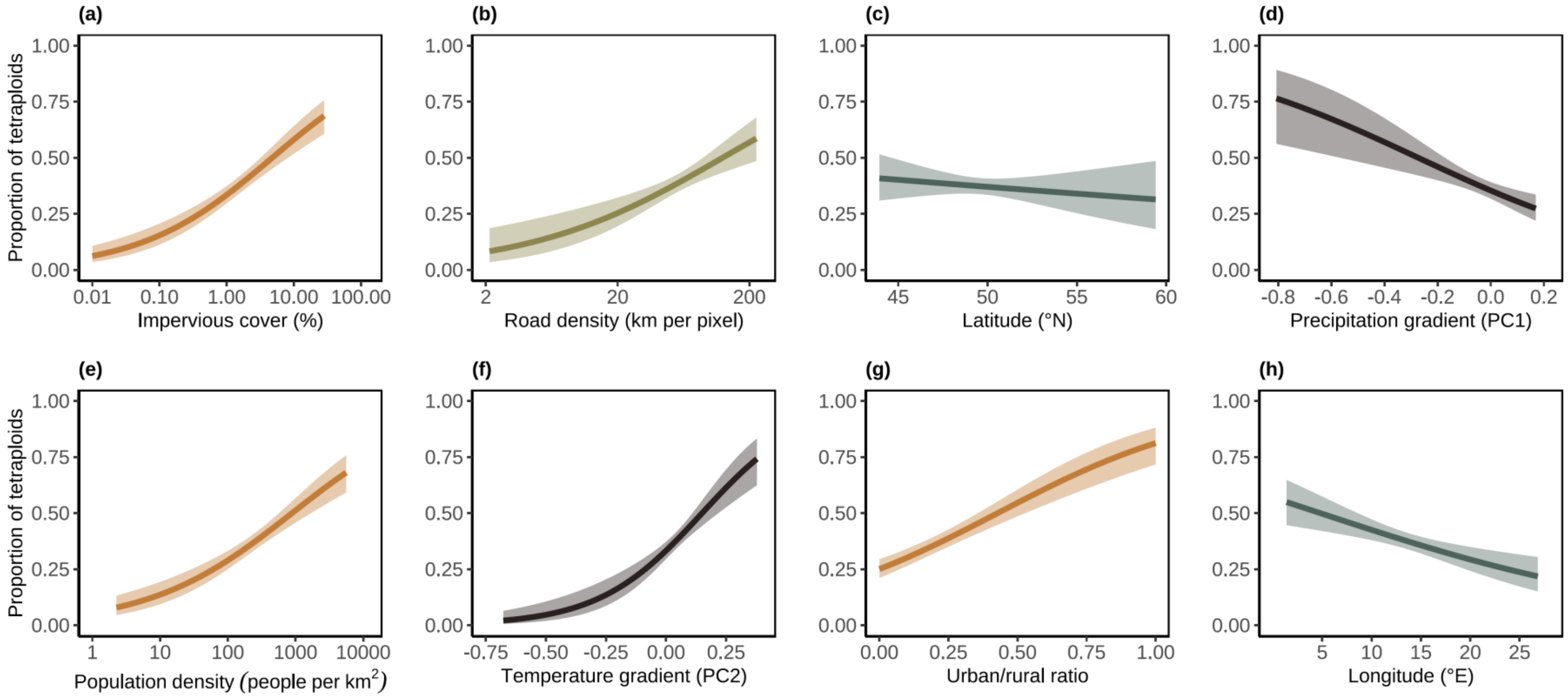

Supplementary Fig. S13. Linear predictors of the current occurrence of tetraploid *Centaurea stoebe* across its expanded range. As a proxy for the current occurrence, we used the proportion of tetraploid relative to all *C. stoebe* records within the timeframe between 1989 and 2023. As predictor variables, three spatial variables (dark green), three climatic variables (black), three variables related to dispersal corridors (olive) and three variables related to urbanization (orange) were tested in a boosted regression tree (BRT) for their effect on the current occurrence of tetraploids in their expanded range (Fig. 5b in the main manuscript). The dispersal corridor and urbanization data were data extracted from 10 km × 10 km pixels from the distinct collection year, i.e., spatio-temporally explicit data. From the 12 explanatory variables, we plot the linear relationships of those predictors that had a significant effect on the proportion of tetraploids in the BRT in order of the relative importance of each predictor in the BRT. The predictor variables included (a) cover of impervious structures, (b) road density, (c) latitude, (d) precipitation gradient, (e) population density, (f) temperature gradient, (g) urban/rural ratio, and (h) longitude. Note that BRTs predict piecewise functions – not a constant linear slope – over the entire data range. For the sake of clarity, however, we here plot constant linear slopes (logit-link function). The impervious cover, road density and population density data are $\log_e$-scaled for plotting.

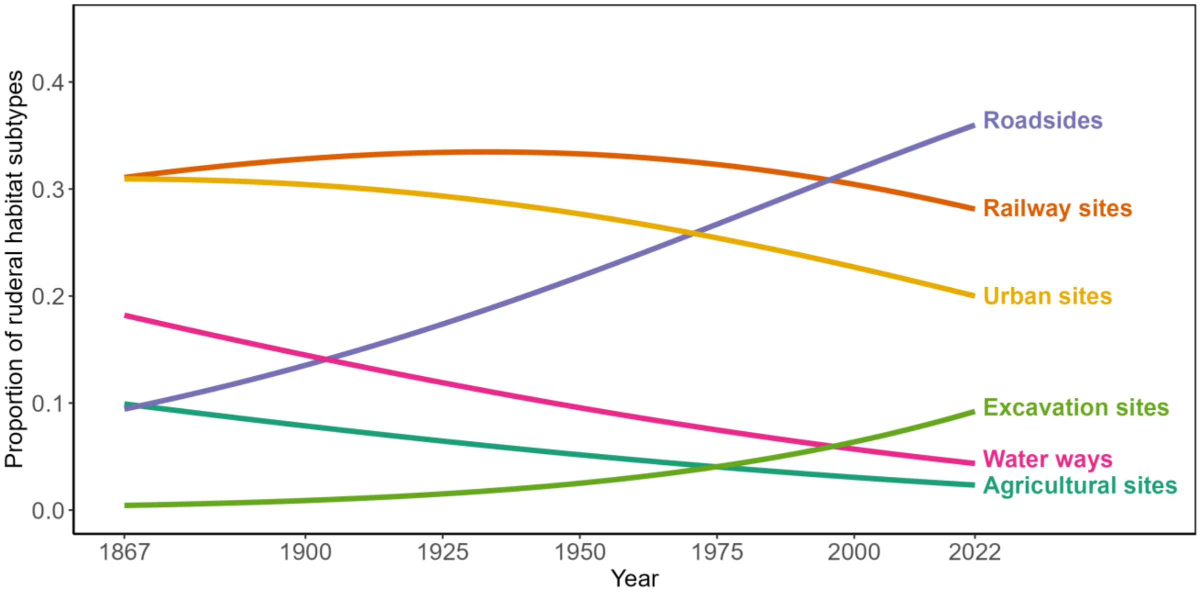

Fig. S14. Predicted proportion of ruderal habitat subtypes that have been colonized by tetraploid *Centaurea stoebe* in its expanded range over time. To get a more detailed picture on which subtypes of ruderal habitats had been colonized in the range expansion of tetraploids, we classified the ruderal habitats into six ruderal habitat subtypes: 1) agricultural sites (farms, fields, vineyards, orchards and fallow land), 2) railway sites (sites along railways, railway embankments and railway stations), 3) roadsides (sites along roads, paths and parking areas), 4) urban sites (variety of habitats related to human life in towns and suburbs such as waste land, cemeteries, public buildings, private property, city walls, gardens, town parks, military areas, airports, camping areas, castles and their ruins), 5) excavation sites (limestone quarries, sand and gravel pits, mining areas and their abandoned fallow lands) and 6) water ways (harbors, dykes, canals and their embankments). We applied multinomial regression to test whether the frequencies of these habitat subtypes changed over time, using the R package nnet 7.3-18 (Venables & Ripley, 2002). The shift in relative habitat subtype frequencies over time was always highly significant, regardless which habitat subtype was set as a reference level in the multinomial regression. Railway sites have been the predominant habitat subtype throughout the first 120 years of observation (relative frequency between 30–35%). However, roadsides have become the most frequently colonized habitat type over the last decades. Presently, almost 40% of tetraploid records in the expanded range are from roadsides.

New Phytologist

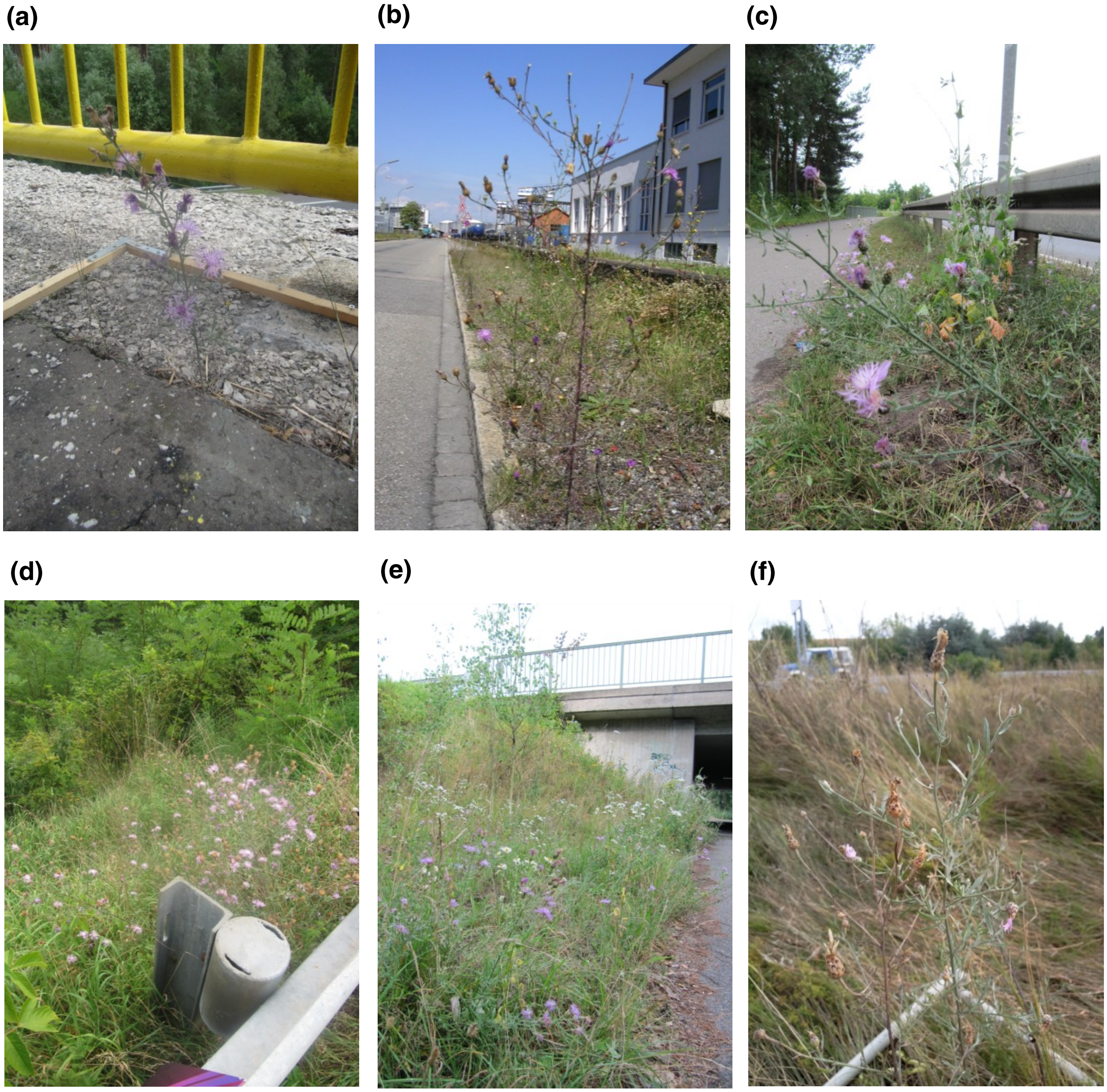

Supplementary Fig. S15. Field impressions from tetraploid *Centaurea stoebe* populations along roadsides in its expanded range. In our field surveys, roadsides were the most frequent habitat type of tetraploids in their expanded range. Tables S3 and S4 list > 500 field sites that we visited over the last 20 years. Photos (a- f) present a disturbance gradient along these roadside populations. (a) Tetraploid individual emerging from asphalt cracks on a highway bridge near Závod, Sklovakia (photo: July 2012 by C. Rosche). (b) Tetraploid individual at a highly disturbed roadside with low vegetation cover in Basel, Switzerland (photo: July 2010 by O. Broennimann) (c) Tetraploid population at a highway edge with moderate vegetation cover near Nuremberg, Germany (photo: July 2012 by M. Hartmann). (d) Tetraploid population in dense vegetation at a roadside in Bitterfeld, Germany (photo: August 2019 by C. Rosche). (e) Tetraploid population in a productive habitat at a roadside in Donji Miholjac, Croatia (photo: August 2023 by C. Rosche). (f) Tetraploid population spreading in a semi-natural grassland adjacent to a roadside near Bovshiv, Ukraine (photo: August 2020 by A. Novikov).

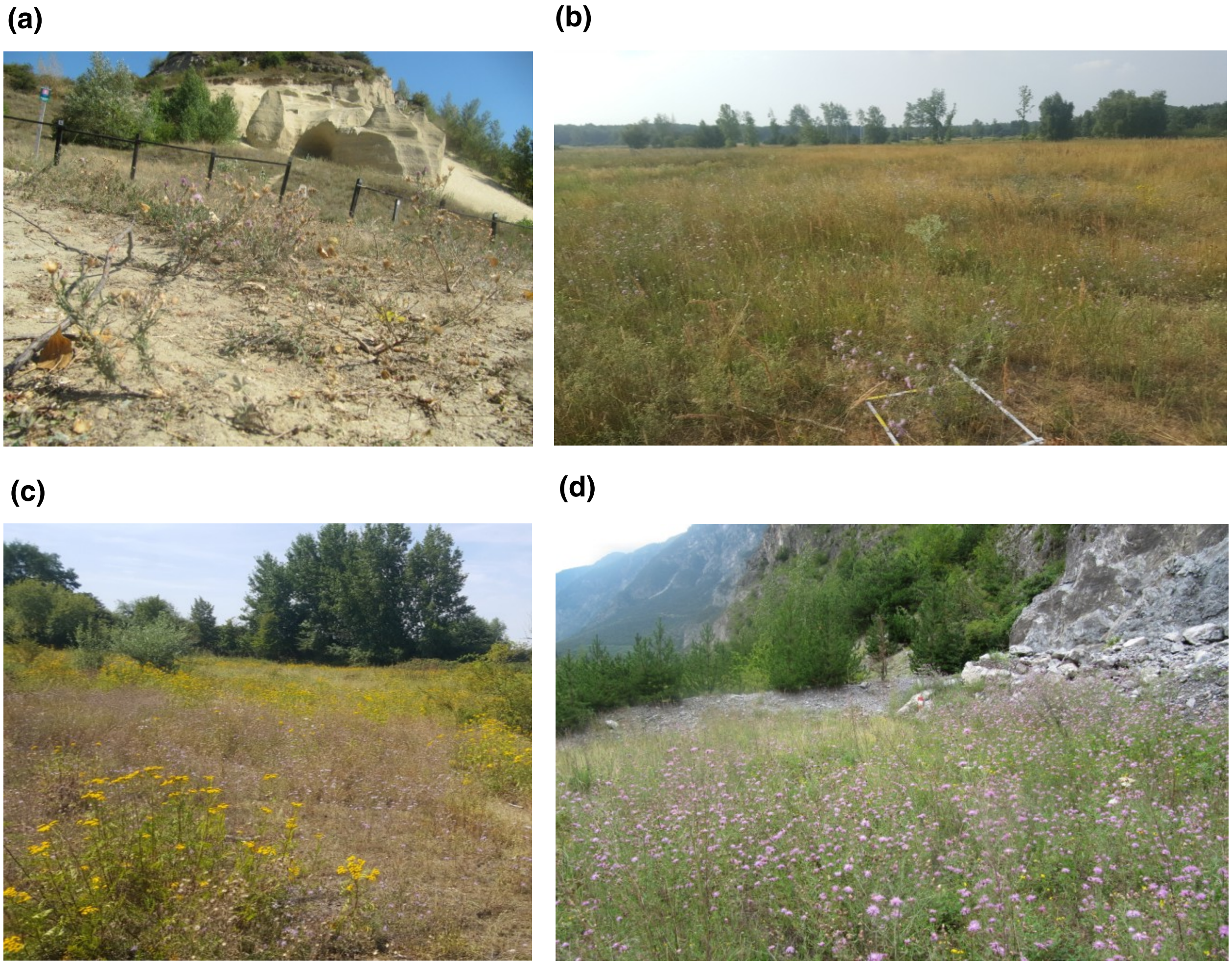

Supplementary Fig. S16. Field impressions from tetraploid *Centaurea stoebe* populations that naturalize into semi-natural vegetation in its expanded range. (a) Tetraploid population spreading into a semi-natural sandy grassland inhabiting a diploid population in the Devínska Kobyla National Nature Reserve, Bratislava, Slovakia (photo: July 2012 by C. Rosche). Spatio-temporal dynamics in the co-occurrences of both cytotypes in this mixed-ploidy population has been monitored in Mráz *et al.* (91). (b) Tetraploid population that naturalizes into a semi-natural sandy grassland near Pánov, Czech Republic (photo: July 2021 by K. Kožić). (c) Tetraploid population that naturalizes into a semi-natural grassland near Nijmegen, Netherlands (photo: August 2020 by Kevin Kožić). (d) Tetraploid population that naturalizes into an alpine rocky meadow near Starkenbach, Austria (photo: August 2012 by M. Hartmann). The plants apparently spread from a close-by parking place.

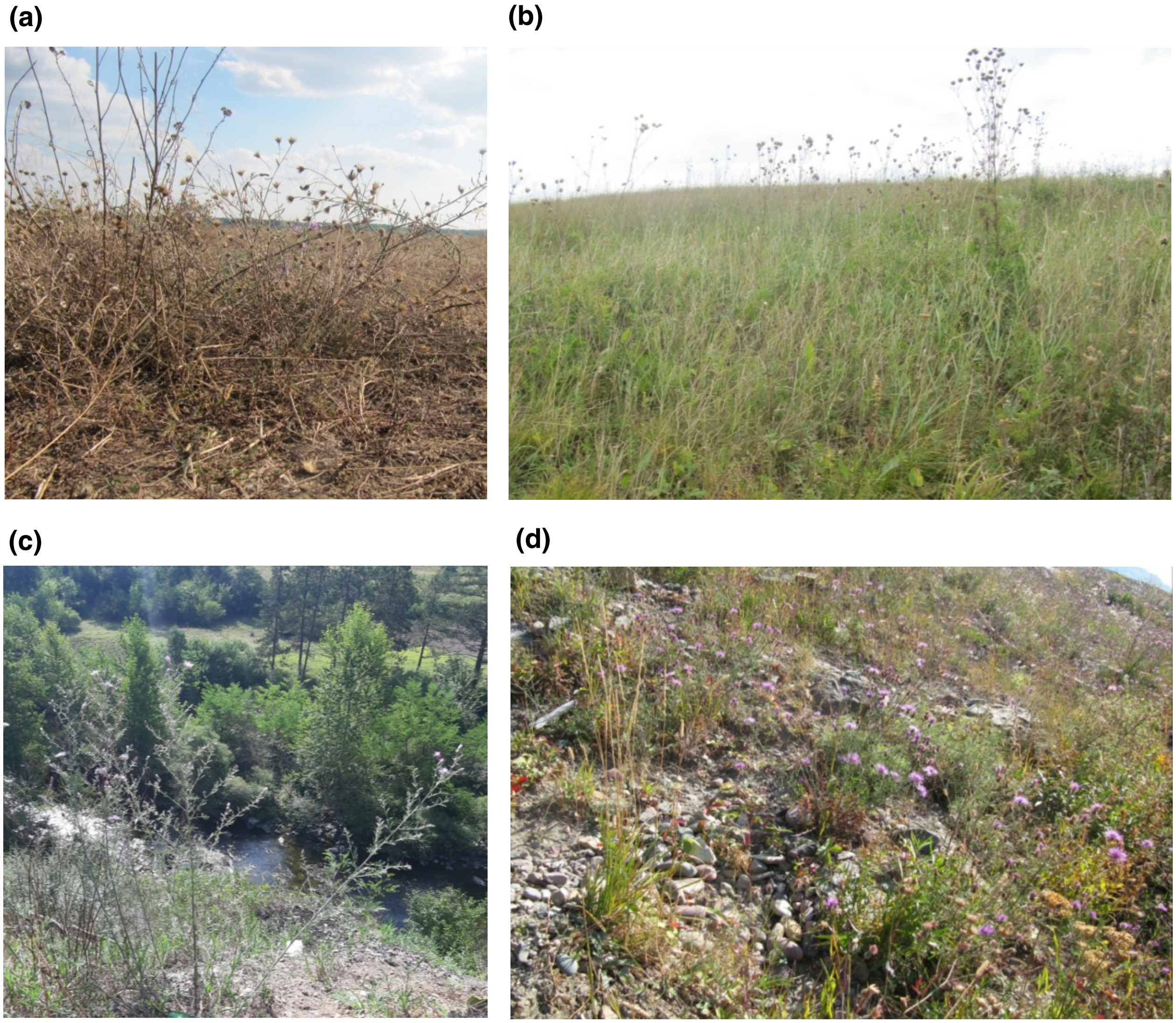

Supplementary Fig. S17. Field impressions from tetraploid *Centaurea stoebe* populations in natural habitats in its native range. (a) Tetraploid population in a steppic grassland near Iași, Romania (photo: July 2012 by C. Rosche). (b) Tetraploid population in a semi-natural dry grassland near Fălticeni, Romania (photo: July 2012 by M. Hartmann). (c) Tetraploid population at a slope near Višegrad, Bosnia and Herzegovina (photo: August 2021 by K. Kožić). (d) Tetraploid population in a mountainous rocky meadow near Poșaga de Jos, Romania (photo: July 2012 by C. Rosche).

Supplementary Table S1. Published studies that used tetraploid *Centaurea stoebe* populations from the expanded range and treated them as native populations.

References	Comparisons made
Broennimann *et al.* (2007)	Between ranges
Broennimann & Guisan (2008)	Between ranges
Broennimann *et al.* (2012)	Between ranges
Broennimann *et al.* (2014)	Between ranges; Between native cytotypes
Broz *et al.* (2009)	Between ranges; Between native cytotypes
Callaway *et al.* (2011)	Between ranges
Collins *et al.* (2011)	Between ranges; Between native cytotypes
Collins & Müller-Schärer (2012)	Between ranges; Between native cytotypes
Collins *et al.* (2013)	Between ranges; Between native cytotypes
Geisen *et al.* (2017)	Between ranges
Geisen *et al.* (2018)	Between ranges
Gfeller *et al.* (2019)	Between ranges
Hahn *et al.* (2012)	Between ranges; Between native cytotypes
Hahn *et al.* (2012b)	Between ranges; Between native cytotypes
Hahn *et al.* (Hahn 2013)	Between ranges; Between native cytotypes
Hahn & Müller-Schärer (2013)	Between ranges; Between native cytotypes
He *et al.* (2009)	Between ranges
He *et al.* (2010)	Between ranges
Henery *et al.* (2010)	Between ranges; Between native cytotypes
Hufbauer & Sforza (2008)	Between ranges; Between native cytotypes
Koorem *et al.* (2018)	Between ranges
Koorem *et al.* (2020)	Between ranges
Koorem *et al.* (2021)	Between ranges
Li *et al.* (2023)	Between ranges
Maron *et al.* (2013)	Between ranges
Maron *et al.* (2014)	Between ranges
Marrs *et al.* (2008)	Between ranges; Between native cytotypes
Mráz *et al.* (2011)	Between ranges; Between native cytotypes
Mráz *et al.* (2012a)	Between ranges; Between native cytotypes
Mráz *et al.* (2014)	Between ranges
Petitpierre *et al.* (2012)	Between ranges
Petitpierre *et al.* (2017)	Between ranges
Ramirez *et al.* (2019)	Between ranges
Ridenour *et al.* (2008)	Between ranges
Rosche *et al.* (2016)	Between ranges; Between native cytotypes
Rosche *et al.* (2017)	Between ranges; Between native cytotypes
Rosche *et al.* (2018)	Between ranges; Between native cytotypes
Schaffner *et al.* (2011)	Between ranges
Shipunov *et al.* (2008)	Between ranges
Španiel *et al.* (2008)	Between native cytotypes

Sun *et al.* (2013)	Between ranges
Sun *et al.* (2014)	Between ranges
Sun *et al.* (2015)	Between ranges
Sun *et al.* (2016)	Between ranges; Between native cytotypes
Thakur *et al.* (2021)	Between ranges
Thébault *et al.* (2010)	Between ranges; Between native cytotypes
Thébault *et al.* (2011)	Between ranges; Between native cytotypes
Thébault *et al.* (2012)	Between ranges
Treier *et al.* (2009)	Between ranges; Between native cytotypes
Wilschut *et al.* (2018)	Between ranges
Wilschut *et al.* (2019)	Between ranges
Wilschut *et al.* (2020)	Between ranges

Note: The reviewed papers focused on one or both of the following comparisons: (1) Between ranges: native vs. non-native tetraploid populations (i.e., investigating post-introduction evolution or ecological differences between the European and North American ranges), and/or (2) Between native cytotypes: diploid vs. tetraploid populations (i.e., investigating pre-adaptive differences between the cytotypes in traits that made tetraploids more likely to become invasive in North America than diploids). We included only studies that explicitly focused on the mentioned comparisons. Doing so, our literature survey was rather conservative because there are many more studies that have used tetraploid populations from their expanded range but without focusing on the mentioned comparison. The listed studies also include comparisons of biotic interactions of tetraploid *C. stoebe* in its native vs. non-native ranges using seeds from competitor species (e.g., Sun *et al.*, 2014) or soil (e.g., Wilschut *et al.* 2018) collected in the *C. stoebe* study populations.

Supplementary Table S2. List of the 167 herbaria where the diploid and tetraploid *Centaurea stoebe* herbarium specimens were deposited.

Acronym	Name of Institution	Location	2x	4x
AA	Kazakhstan Academy of Sciences	Alma-Ata, Kazakhstan	3	0
AAU	Aarhus University	Aarhus, Denmark	1	9
ACAD	Acadia University	Wolfville, Canada	0	1
ALTB	Altai State University	Barnaul, Russia	2	0
AMD	Naturalis Biodiversity Center	Leiden, Netherlands	21	2
AUR	Volcano Museum	Aurillac, France	2	0
B	Botanical Garden and Botanical Museum Berlin	Berlin, Germany	205	56
BASBG	Basel University	Basel, Switzerland	25	11
BEOU	University of Belgrade	Belgrade, Serbia	3	5
BG	University of Bergen	Bergen, Norway	0	1
BIL	Białystok University of Technology	Białystok, Poland	22	0
BILAS	Center for Natural Research	Vilnius, Lithuania	23	0
BM	Natural History Museum	London, U.K.	1	1
BNL	Federal Agency for Nature Conservation	Saarbrücken, Germany	15	5
BOCH	Ruhr-University Bochum	Bochum, Germany	0	1
BOVB	Botanical Society Berlin-Brandenburg	Berlin, Germany	4	0
BP	Hungarian Natural History Museum	Budapest, Hungary	10	52
BR	Meise Botanic Garden	Meise, Belgium	8	15
BRA	Slovak National Museum	Bratislava, Slovakia	70	25
BRE	University of Brittany	Brest, France	16	0
BRIT	Botanical Research Institute of Texas	Fort Worth, U.S.A.	0	1
BRNM	Moravian Museum	Brno, Czech Republic	119	11
BRNU	Masaryk University	Brno, Czech Republic	141	32
BUC	Botanical Garden D. Brandza	Bucharest, Romania	0	11
BRY	Brigham Young University	Provo, U.S.A.	0	1
BUCA	Romanian Academy of Sciences	Bucharest, Romania	8	10
BUNS	University of Novi Sad	Novi Sad, Serbia	7	67
C	University of Copenhagen	Copenhagen, Denmark	22	4
CHE	National Society of Sciences	Cherbourg, France	3	0
CHER	Yu. Fedcovich Chernivtsi State University	Chernivtsi, Ukraine	26	38
CHIS	Academy of Sciences of Moldova	Kishinev, Moldova	3	4
CL	Babes-Bolyai University	Cluj-Napoca, Romania	53	64
CLF	University Clermont Auvergne	Clermont-Ferrand, France	10	0
COI	University of Coimbra	Coimbra, Portugal	2	0
COLO	University of Colorado Museum of Natural History	Boulder, U.S.A.	2	1
CWU	V. N. Karazin National University	Kharkiv, Ukraine	5	3
DNZ	Donetsk Botanical Garden	Donetsk, Ukraine	45	9
DR	Technical University Dresden	Dresden, Germany	22	3
EGR	Eszterházy Károly University of Applied Sciences	Eger, Hungary	0	1
FR	Senckenberg Society for Natural Research	Frankfurt, Germany	115	46
FULD	Association for Natural History in East Hessen	Fulda, Germany	0	2
G	Conservatory and Botanical Garden of Geneva	Geneva, Switzerland	34	9
GB	University of Gothenburg	Gothenburg, Sweden	16	9

GE	University of Genoa	Genoa, Italy	1	0
GFW	Ernst-Moritz-Arndt-University	Greifswald, Germany	23	2
GJO	Universal Museum Joanneum	Graz, Austria	35	17
GLM	Senckenberg Society for Natural Research	Görlitz, Germany	21	14
GOET	University of Göttingen	Göttingen, Germany	38	12
GZU	Karl-Franzens-University Graz	Graz, Austria	97	20
H	University of Helsinki	Helsinki, Finland	16	15
HAL	Martin-Luther-University Halle-Wittenberg	Halle, Germany	45	17
HALN	State Agency for Environmental Protection	Halle, Germany	2	0
HBG	University of Hamburg	Hamburg, Germany	40	15
IASI	University Ion Ionescu de la Brad	Iași, Romania	20	43
IB	University of Innsbruck	Innsbruck, Austria	24	2
IND	Indiana University	Bloomington, U.S.A.	1	0
IRK	Siberian Branch of the Russian Academy of Sciences	Irkutsk, Russia	5	0
ISTE	University of Istanbul	Istanbul, Turkey	1	0
JE	Friedrich Schiller University Jena	Jena, Germany	28	8
JPU	Janus Pannonius University	Pécs, Hungary	7	5
K	Royal Botanic Gardens	London, U.K.	1	0
KAZ	Kazan State University	Kazan, Russia	21	0
KIEL	Christian-Albrechts-University Kiel	Kiel, Germany	10	4
KO	P. J. Šafárik University in Košice	Košice, Slovakia	16	0
KRAM	Polish Academy of Sciences	Kraków, Poland	136	13
KSPI	Kostanay State Pedagogical Institute	Kostanay, Kazakhstan	1	0
KUZ	Siberian Branch of the Russian Academy of Sciences	Kemerovo, Russia	11	0
KW	National Herbarium of Ukraine	Kyiv, Ukraine	135	20
KWHA	Ukrainian National Academy of Sciences	Kyiv, Ukraine	21	6
KWHU	Taras Shevchenko National University of Kyiv	Kyiv, Ukraine	1	4
KWU	O.V. Fomin Botanical Garden	Kyiv, Ukraine	23	14
L	Naturalis Biodiversity Center	Leiden, Netherlands	69	17
LE	Komarov Botanical Institute of RAS	Saint Petersburg, Russia	28	20
LI	Upper Austrian Regional Culture GmbH	Linz, Austria	4	0
LJU	University of Ljubljana	Ljubljana, Slovenia	28	7
LMS	Natural History Museum of Le Mans	Le Mans, France	1	0
LUX	National Museum of Natural History	Luxembourg, Luxembourg	4	0
LW	Ivan Franko National University	Lviv, Ukraine	20	3
LWS	State Museum of Natural History	Lviv, Ukraine	45	8
LWKS	Institute of Ecology of the Carpathians	Lviv, Ukraine	27	8
LY	University Claude Bernard	Lyon, France	27	6
LYJB	Botanical Garden of Lyon	Lyon, France	1	0
LZ	University of Leipzig	Leipzig, Germany	16	15
M	State Natural History Collections of Bavaria	Munich, Germany	116	73
MA	Royal Botanical Garden	Madrid, Spain	0	0
MARS	Aix-Marseille University	Marseille, France	1	0
MHA	Main Botanical Garden Russian Academy of Sciences	Moscow, Russia	16	0
MNVD	Museum of Natural History and Prehistory Dessau	Dessau-Roßlau, Germany	10	2
MPU	University of Montpellier	Montpellier, France	40	0

MSK	National Academy of Sciences of Belarus	Minsk, Belarus	24	0
MSTR	Museum of Natural Science	Münster, Germany	15	0
MSUD	I. I. Mecynikov State University of Odessa	Odessa, Ukraine	4	0
MW	Moscow State University	Moscow, Russia	164	29
NCY	Conservatory and Botanical Gardens of Nancy	Nancy, France	3	1
NEU	University of Neuchâtel	Neuchâtel, Switzerland	6	3
NHMF	Natural History Museum Fribourg	Fribourg, Switzerland	9	12
NHMS	Natural History Museum Split	Split, Croatia	2	0
NIS	Herbarium Moesiacum Niš	Niš, Serbia	11	13
NLOE	State Office for Ecology in Lower Saxony	Hannover, Germany	0	2
NLU	University of Louisiana at Monroe	Monroe, U.S.A.	0	1
NML	Natural History Museum Leipzig	Leipzig, Germany	7	12
NS	Central Siberian Botanical Garden	Novosibirsk, Russia	3	0
NSK	Siberian Branch of Russian Academy of Sciences	Novosibirsk, Russia	4	0
NY	The New York Botanical Garden	New York, U.S.A.	1	0
O	University of Oslo	Oslo, Norway	3	7
OCHS	Herbarium Jörg Ochsmann	Halle, Germany	48	28
OXF	University of Oxford	Oxford, U.K. England	1	0
P	National Museum of Natural History	Paris, France	73	22
PI	University of Pisa	Pisa, Italy	4	0
POLL	Palatinate Museum of Natural History	Bad Dürkheim, Germany	21	3
PR	National Museum in Prague	Prague, Czech Republic	462	36
PRC	Charles University	Prague, Czech Republic	229	48
PRISH	University of Priština	Priština, Kosovo	1	0
PVB	Institute of Ecology of the Volga River Basin	Togliatti, Russia	20	2
REG	Botanical Society of Regensburg	Regensburg, Germany	1	1
RSA	California Botanic Garden	Claremont, U.S.A.	2	1
RV	Southern Federal University	Rostov-on-Don, Russia	4	0
SAAR	Center for Bio-documentation of the Saarland	Saarbrücken, Germany	6	13
SAMU	Savaria County Municipal Museum	Szombathely, Hungary	6	0
SARA	National Museum of Bosnia and Herzegovina	Sarajevo, Bosnia and [illegible]	1	12
SAV	Slovak Academy of Sciences	Bratislava Slovakia	17	4
SIB	Natural History Museum	Sibiu, Romania	8	27
SLA	Sciences et Arts de l'Aveyron	Rodez Cedez, France	2	0
SLTC	Pomeranian University in Słupsk	Słupsk, Poland	2	0
SO	Sofia University	Sofia, Bulgaria	0	4
SOM	Bulgarian Academy of Sciences	Sofia, Bulgaria	3	1
STU	State Museum of Natural History	Stuttgart, Germany	75	8
TAA	Estonian University of Life Sciences	Tartu, Estonia	4	0
TALL	Tallinn Botanic Garden	Tallinn, Estonia	1	0
TAM	Estonian Museum of Natural History	Tallinn, Estonia	5	0
TK	Tomsk State University	Tomsk, Russia	10	1
TKM	The State Institution of Tula Region Culture	Tula, Russia	1	0
TOM	Consolata Missions Institute	Turin, Italy	1	0
TRH	Norwegian University of Science and Technology	Trondheim, Norway	0	2
TROM	The Arctic University of Norway	Tromsø, Norway	0	2

TRT	Royal Ontario Museum	Toronto, Canada	0	1
TSB	University of Trieste	Trieste, Italy	14	2
TU	University of Tartu	Tartu, Estonia	3	0
TUL	Tula State Lev Tolstoy Pedagogical University	Tula, Russia	6	0
TUR	University of Turku	Turku, Finland	0	6
U	Naturalis Biodiversity Center	Leiden, Netherlands	11	2
UBT	University of Bayreuth	Bayreuth, Germany	0	1
UFA	Ufa Scientific Centre, Russian Academy of Sciences	Ufa, Russia	19	0
UM	Uman National University of Horticulture	Uman, Ukraine	0	4
UPS	Museum of Evolution	Uppsala, Sweden	28	19
US	Smithsonian Institution	Washington D.C., U.S.A.	9	5
USCH	University of South Carolina	Columbia, U.S.A.	1	0
UU	Uzhhorod National University	Uzhhorod, Ukraine	0	12
VLA	Far Eastern Branch, Russian Academy of Sciences	Vladivostok, Russia	3	0
VO	Vologda State University	Vologda, Russia	1	0
VOR	Voronezh State University	Voronezh, Russia	17	0
VTA	National Research Institute	Antibes-Juan, France	1	0
W	Natural History Museum Vienna	Vienna, Austria	33	14
WA	University of Warsaw	Warsaw, Poland	36	4
WAG	Naturalis Biodiversity Center	Leiden, Netherlands	6	0
WRSL	Wroclaw University	Wroclaw, Poland	35	6
WU	University of Vienna	Vienna, Austria	11	15
YALT	The State Nikita Botanical Gardens	Yalta, Ukraine	6	2
Z	University of Zurich	Zurich, Switzerland	58	5
ZA	University of Zagreb	Zagreb, Croatia	1	0

Note: Herbarium acronyms follow Thiers (2023). BOVB, NIS, NLOE, OCHS, and PRISH are not listed in Thiers (2023), but are our created abbreviations. 2x and 4x refer to the numbers of diploid and tetraploid specimens that have been included in our total dataset after filtering, respectively (see Fig. S2). BRA, CL, LE and SAV have been surveyed in Broennimann *et al.* (2014). TSB, BP, W, LY, GOET and G were surveyed in Ochsmann (2000). M, JE, P, WU and OCHS have been partly surveyed in Ochsmann (2000) and were revisited to include collections after 2000.

Supplementary Table S3. Previously published cytogeographic records of diploid and tetraploid *Centaurea stoebe* populations.

Sources	Populations
Guinochet (1957)	3
Baksay (1958)	1
Skalińska *et al.* (1959)	7
Záborský (1970)	2
Gochu (1973)	2
Damboldt & Matthas (1975)	2
Kuzmanov & Georgieva (1976)	2
Kuzmanov & Georgieva (1977)	1
van Loon & de Jong (1978)	1
Kuzmanov *et al.* (1981)	2
Andreev (1981)	1
Löve & Löve (1982)	1
Lovrić (1982)	2
van Loon & van Setten (1982)	3
Georgiadis (1983)	4
Kuzmanov *et al.* (1986)	1
Agapova *et al.* (1990)	1
Kiehn *et al.* (1991)	1
Ochsmann (1999)	14
Lövkvist & Hultgård (1999)	1
Kiehn *et al.* (2000)	1
Nagel (2000)	14
Bancheva & Greilhuber (2006)	6
Gregor & Hand (2008)	14
Španiel *et al.* (2008)	41
Treier *et al.* (2009)	83
Callaway *et al.* (2011)	5
Mráz *et al.* (2011)	2
Mráz *et al.* (2012a)	19
Mráz *et al.* (2012b)	16
Probatova *et al.* (2013)	1
Pustahija *et al.* (2013)	2
Otisková *et al.* (2014)	123
Rosche *et al.* (2016)	42
Carev *et al.* (2017)	1
Mártonfiová *et al.* (2018)	1
Šmarda *et al.* (2019)	1
Kožić *et al.* (2024)	140

Note: Sources refer to recent publications that used chromosome counts or flow cytometry to determine the ploidy level of their investigated *C. stoebe* populations. Populations refer to the number of occurrence data from each source included into our dataset.

Supplementary Table S4. List of unpublished cytogeographic records of *Centaurea stoebe* populations.

Cytotype	Locality	Collector	Latitude	Longitude	Date
Diploid	Austria, Oeynhausen, agricultural field	Kožić, K.	47.978	16.271	2020-09-11
Diploid	Austria, Pfaffstätten, meadow near houses	Kožić, K.	48.022	16.262	2020-09-11
Diploid	Bulgaria, Rila Monastery, schist rocks along the road to the Rilski Monastyr	Mráz, P. & Mrázová, V.	42.115	23.32	2008-08-01
Diploid	Czech Republic, Děčín, winter harbor	Hadinec, J.	50.765	14.206	2013-10
Diploid	Czech Republic, Prague, roadside near the station Modřanský cukrovar	Rosche, C.	49.999	14.406	2014-10
Diploid	Czech Republic, Prague, Nádraží Modřany, along the railway	Rosche, C.	50.004	14.403	2014-10
Diploid	Czech Republic, Kleneč, steppe meadow	Mráz, P.	50.39	14.257	2014-05
Diploid	Czech Republic, Bezděz, basaltic quarry	Mráz, P.	50.538	14.714	2014-05
Diploid	Czech Republic, Bezděz, railway station	Hadinec, J.	50.534	14.722	2015-07
Diploid	Czech Republic, Kolín, railway station	Štefánek, M.	50.026	15.212	2016
Diploid	Czech Republic, Lysá nad Labem-Dvorce, fen meadow	Mráz, P.	50.21	14.806	2016-05-26
Diploid	Czech Republic, Mnichovice, remnants of dry meadow – clearing at the oak forest margin	Mráz, P.	49.939	14.699	2017-08-11
Diploid	Czech Republic, Prague, Štvanice island, gravel sediments of Vltava river on the S bank	Štefánek, M.	50.097	14.449	2021-10-18
Diploid	France, Gigors, roadside	Mráz, P. & Mrázová, V.	44.806	5.11	2008
Diploid	France, Prades	Mráz, P. & Mrázová, V.	45.031	3.595	2008
Diploid	France, Polignac	Mráz, P. & Mrázová, V.	45.07	3.86	2008
Diploid	France, Le Chambon, basaltic rocks above the road N of the village	Mráz, P. & Mrázová, V.	45.147	3.464	2008
Diploid	France, Chilhac, dry slope above the road	Mráz, P. & Mrázová, V.	45.157	3.444	2008
Diploid	France, Haute Loire, château de Leotoing	Mráz, P. & Priestman, L.	45.359	3.226	2008-05-01
Diploid	France, Alsace, Bollenberg, dry grassland	Rosche, C.	47.94	7.263	2017-06-21
Diploid	Germany, Wolfen, meadow	Rosche, C.	51.67	12.267	2015
Diploid	Germany, Querfurt, on the walls of the castle	Rosche, C.	51.377	11.592	2017-05-31
Diploid	Germany, road from Seeburg to Höhnstedt	Rosche, C.	51.5	11.702	2017-07-12
Diploid	Germany, along the road from Wimmelburg to Wolferode	Rosche, C.	51.512	11.509	2017-07-19
Diploid	Germany, near Nature reserve “NSG Hasenwinkel”, slopes with dry grassland, open vegetation	Rosche, C.	51.519	11.614	2017-05-18
Diploid	Germany, Landsberg along the railway	Rosche, C.	51.525	12.148	2017-07-27
Diploid	Germany, near Rothenburg/Nelbe in dry meadows Nature reserve “NSG Zickeritzer Busch”	Rosche, C.	51.648	11.748	2017-08-04

Diploid	Germany, Saxony-Anhalt, Nelben near Rothenburg, dry grassland	Rosche, C.	51.655	11.746	2017-08-19
Diploid	Germany, Saxony-Anhalt, Bernburg, roadside	Rosche, C.	51.783	11.754	2017-07-30
Diploid	Germany, Bernburg, northern entry of the town, roadside	Rosche, C.	51.806	11.724	2017-08-08
Diploid	Germany, Gerwisch, inland dune, dry grassland on sands	Rosche, C.	52.192	11.737	2017-06-03
Diploid	Germany, Kolpin, Reichenwalde, dry grassland	Rosche, C.	52.291	13.986	2018
Diploid	Germany, Siemser Wiesen, Lübeck, meadow	Rosche, C.	53.901	10.761	2018
Diploid	Germany, near Neuenburg, Freyburg (Unstrut), dry grassland	Rosche, C.	51.21	11.777	2019-07-12
Diploid	Germany, Langer Berg, Unstrut valley, dry grassland	Rosche, C.	51.228	11.723	2019-07-29
Diploid	Germany, Nüssenberg, Unstrut valley, dry grassland	Rosche, C.	51.229	11.71	2019-07-29
Diploid	Germany, Saxony-Anhalt, Seeburg, dry grassland	Rosche, C.	51.5	11.7	2019-09-23
Diploid	Germany, highway stop “Seeberg West”, next to a gas station	Rosche, C.	52.543	13.693	2020-07-16
Diploid	Germany, Speck (Müritz), dry grassland	Rosche, C.	53.439	12.84	2020
Diploid	Germany, parking lot Belling	Rosche, C.	53.553	13.965	2020
Diploid	Germany, Mecklenburg-Western Pomerania, Belling, along the road	Rosche, C.	53.554	13.966	2020-07-20
Diploid	Germany, Mücheln (Geiseltal), ruderal area	Rosche, C.	51.299	11.773	2021
Diploid	Poland, Żyrardów, dry grassland	Kreiger, G.	52.064	20.465	2020
Diploid	Romania, Munţii Apuseni Mts, Săcuieu, rocks in the NW part of the village	Mráz, P. & Štefánek, M.	46.829	22.877	2014-08-12
Diploid	Romania, Munţii Apuseni Mts, Bologa, road margin, at the foothill of calcareous hill	Mráz, P. Štefánek, M.	46.872	22.877	2014-08-11
Diploid	Romania, Muntele Mare, Cheile Runcului	Şuteu, A. & Goia, I.	46.507	23.442	2019-08-26
Diploid	Russia, Khakasia, Minusinsk Basin, eastern periphery of Abakan City	Danihelka, J.	53.708	91.467	2004-07-29
Diploid	Russia, Altay Kray, roadside 5 km SW from Barnaul	Silantyeva, M.	53.18	83.883	2016
Diploid	Serbia, Vranje, ruins of medieval castle above the town	Mráz, P. & Mrázová, V.	42.583	21.889	2008-07-30
Diploid	Slovakia, Nové Mesto nad Váhom, steppe meadow on Mt. Plešivec	Mráz, P.	48.768	17.816	2008
Diploid	Slovakia, Gelnica	Mráz, P. & S. & Mrázová, V.	48.854	20.927	2008
Diploid	Slovakia, Trenčianska Bohuslavice, forest edge near houses	Kožić, K.	48.802	17.868	2021-07-18
Tetraploid	Belgium, Kieldrecht-Havenvlakte	Pieters, A.	51.283	4.24	2020-09-05
Tetraploid	Belgium, Midas, roadside	Pieters, A.	51.3	4.256	2020-09-05
Tetraploid	Bosnia and Herzegovina, Odvojak, roadsite ar river	Kožić, K.	44.232	18.439	2021-07-26
Tetraploid	Bulgaria, Mezdra, abandoned pasture	Mráz, P. & Mrázová, V.	43.009	23.704	2008-08-03
Tetraploid	Bulgaria, Pleshivets, steppe meadows	Mráz, P. & Mrázová, V.	43.592	22.859	2008-08-03

Tetraploid	Czech Republic, Lovosice, Lovosice train station	Zdvořák, P.	50.51	14.058	2015-09-02
Tetraploid	Czech Republic, Litoměřice, central part of the Litoměřice railway station upper railway station	Zdvořák, P.	50.541	14.13	2015-08-11
Tetraploid	Czech Republic, Lysá nad Labem-Dvorce, railway station	Mráz, P.	50.2	14.801	2016-05-26
Tetraploid	Czech Republic, Prague, freight station Žižkov, on the tracks	Hadinec, J.	50.086	14.478	2018-11
Tetraploid	Germany, Katzwang, railway station	Reger, P.	49.36	11.04	2008-05-05
Tetraploid	Germany, Morenbrunn, Moorenbrumfeld	Reger, P.	49.4	11.19	2008-08-03
Tetraploid	Germany, Nuremberg, railway station	Reger, P.	49.47	11.11	2008-08-28
Tetraploid	Germany, Tempelhofer Park (historical airport Berlin-Tempelhof)	Rosche, C.	52.475	13.392	2011
Tetraploid	Germany, Munich, Dreimühlen, ruderal habitat at a parking plot	Rosche, C. & Hartmann, M.	48.12	11.56	2012
Tetraploid	Germany, Munich, Hartmannshofen, ruderal habitat between a railway and roadside	Rosche, C. & Hartmann, M.	48.17	11.49	2012
Tetraploid	Germany, Bruckdorf near Halle (Saale), roadside	Rosche, C.	51.446	12.032	2016
Tetraploid	Germany, Halle, along the B6 road, near shopping mall HEP	Rosche, C.	51.449	12.022	2017-06-19
Tetraploid	Germany, lake shore of Seese-West, former opencast mine	Rosche, C.	51.82	13.916	2017-08-10
Tetraploid	Germany, Leipzig-Lützschena, railway station	Rosche, C.	51.389	12.299	2018-09-10
Tetraploid	Germany, Röttenbach, landfill	Rosche, C.	49.652	10.919	2019-12-30
Tetraploid	Germany, Bavaria, Würzburg, ruderal area	Rosche, C.	49.801	9.936	2019-11-26
Tetraploid	Germany, Maximiliansau, roadside near factory premises	Kožić, K.	49.036	8.301	2020-07-28
Tetraploid	Germany, near Gernsheim, sandy meadow	Kožić, K.	49.731	8.506	2020-07-28
Tetraploid	Germany, Mönchhof, industrial site, roadside	Kožić, K.	50.035	8.479	2020-07-27
Tetraploid	Germany, Hoyerswerda, ruderal area	Rosche, C.	51.456	14.314	2020
Tetraploid	Germany, Halle, industrial area Halle East	Rosche, C.	51.482	12.095	2020-08-18
Tetraploid	Germany, Bitterfeld, parking area near to Lake Goitsche	Rosche, C.	51.618	12.338	2020-08-16
Tetraploid	Germany, Heimburg, abandoned field	Rosche, C.	51.826	10.917	2020-08-12
Tetraploid	Romania, Banat, Svinița, schist slopes above the road	Mráz, P. & Mrázová, V.	44.55	22.04	2008-08-05
Tetraploid	Romania, Banat, calcareous slopes along the road north of Anina	Mráz, P. & Mrázová, V.	45.158	21.873	2008-08-05
Tetraploid	Romania, Apuseni Mts, ca 3 km S of the village of Posaga, calcareous conglomerate	Mráz, P. & Mrázová, V.	46.427	23.455	2008-08-07
Tetraploid	Romania, Cozia Mts., rocks above the road	Šingliarová, B.	45.32	24.33	2011
Tetraploid	Romania, Mesteacăn, dry slope NE of the village	Mráz, P. & Mrázová, V.	47.4	23.51	2011
Tetraploid	Romania, Braşov, NE part of the town, on the road margin of Strada Hărmanului street	Mráz, P. & Štefánek, M.	45.674	25.647	2014-08-09
Tetraploid	Romania, Cârţişoara, pastures on the gravel sediments of river bed	Štefánek, M.	45.707	24.57	2015-08-13

Tetraploid	Romania, Lunca, roadside of dirt road	Štefánek, M.	45.99	22.876	2015-08
Tetraploid	Romania, Porumbeşti, degraded grassland between fishpond and road	Filep, R.	47.572	22.592	2021-08-18
Tetraploid	Serbia, Kopaonik Mts, Brzeće, along the road, limestone	Mráz, P. & Mrázová, V.	43.306	20.868	2008-07-29
Tetraploid	Serbia, Kopaonik Mts., Brzeće, along the road, limestone	Mráz, P. & Mrázová, V.	43.311	20.866	2008-07-29
Tetraploid	Serbia, Stari Slankamen, east of Novi Sad, sandy slopes	Mráz, P. & Mrázová, V.	45.141	20.254	2008-07-28
Tetraploid	Serbia, Kopaonik Mts, Brzeće, on the serpentines NE of the village, along a local road	Kolář, F.	43.306	20.9	2013-05-16
Tetraploid	Serbia, Golubac, on the limestone rock above the road Golubac–Dobra	Kolář, F.	44.658	21.662	2013-05-16
Tetraploid	Slovakia, Senec, along the highway Bratislava–Senec northwest of the town	Mráz, P. & Mrázová, V.	48.239	17.372	2008-08
Tetraploid	Slovakia, Veľké Zálužie west of Nitra, motorway parking	Mráz, P.	48.325	17.945	2009
Tetraploid	Slovakia, Nové Mesto nad Váhom, Trenčianska street, roadside	Mráz, P.	48.763	17.844	2011
Tetraploid	Slovakia, Nové Mesto nad Váhom, Izbická street, roadside at the crossroad	Mráz, P.	48.765	17.835	2011
Tetraploid	Slovakia, Bratislava-Nové Mesto	Mráz, P.	48.187	17.134	2013
Tetraploid	Slovakia, village of Naháč, ruin of medieval monastery	Mráz, P. & Mrázová, V.	48.555	17.536	2014-05
Tetraploid	Slovakia, Bratislava-Petržalka, OMV gas station	Kožić, K.	48.088	17.098	2021-07-19
Tetraploid	Sweden, Skåne county, Åhus	Tyler, T.	55.925	14.298	2014
Tetraploid	Switzerland, Ganterschwill, grassland meadow in between roads	Treier, U. & Müller-Schärer, H.	47.378	9.085	2010
Tetraploid	Switzerland, roadside toward Salvan	Rosche, C.	46.114	7.052	2014
Tetraploid	Switzerland, Bière, military zone - training area of tanks and artillery	Rosche, C.	46.526	6.33	2014
Tetraploid	U.K., Aberford, Leeds, road verges	Wilcox, M.	53.843	-1.343	2020-09-25

Notes: Flow cytometry was performed according to the protocol in Mráz *et al.* (2011). For some of the flow cytometrical records, there are voucher specimens deposited in NHMF and PRC (samples collected by P. Mráz, J. Hadinec, F. Kolář, V. Mrázová, M. Štefánek and P. Zdvořák), BRNU (J. Danihelka), CL (A. Şuteu and I. Goia) and HAL (K. Kožić and C. Rosche).

Supplementary Table S5. Model comparisons of the generalized additive models (GAMs) predicting the proportion of tetraploid relative to all *Centaurea stoebe* records (P_T) as a function of time, range, and habitat type.

Model	Df	Expl. dev.	AIC
**(1) Accounting for spatial autocorrelation**			
P_T~s(year)+s(lat, long, bs="sos")	50.8	38%	4131.2
P_T~s(year)+s(lat, long, bs="tp")	31	34.2%	4336.3
P_T~s(year)+s(country, bs="re")	32.7	31.2%	4535.5
P_T~s(year)	6.2	4.4%	6222
**(2) Different smooths between both ranges**			
P_T~s(year, by range)+range+s(lat, long, bs="sos")	52.7	38.7%	4091
P_T~s(year)+range+s(lat, long, bs="sos")	51.6	38.1%	4128.1
P_T~s(year)+s(lat, long, bs="sos")	50.8	38%	4131.2
**(3) Different smooths between habitat types in the native range**			
P_T~s(year, by habitat type)+habitat type+s(lat, long, bs="sos")	26.8	26.3%	827.3
P_T~s(year)+habitat type+s(lat, long, bs="sos")	22.1	25.9%	821.6
P_T~s(year)+s(lat, long, bs="sos")	21.7	25.7%	823
**(4) Different smooths between habitat types in the expanded range**			
P_T~s(year, by habitat type)+habitat type+s(lat, long, bs="sos")	48.7	43.7%	2013.7
P_T~s(year)+habitat type+s(lat, long, bs="sos")	42.5	43.1%	2022
P_T~s(year)+s(lat, long, bs="sos")	45.7	37.2%	2230.8

Notes: The table is subdivided in four parts: (1) To account for spatial autocorrelation, we compared GAMs with different smoothing terms for latitude and longitude: "sos" (spline-on-the-sphere), "tp" (thin plate), and "re" (random effect using country instead of information on latitude and longitude). (2) We analyzed whether temporal dynamics in the P_T differed between native and expanded ranges. In the models below, we analyzed whether temporal dynamics in the P_T differed between the natural and ruderal habitat types, separately in the native (3) and in the expanded ranges (4). Abbreviations: P_T, proportion of tetraploid *Centaurea stoebe*; lat, latitude; long, longitude, Df, degrees of freedom, Expl. dev., percentage of explained deviance; AIC, Akaike information criterion.

Supplementary References

Adler B, Adler J, Kunzmann G (Eds.). 2017. *Flora von Nordschwaben: die Farn- und Blütenpflanzen der Landkreise Dillingen a.d. Donau und Donau-Ries: floristische Rasterkartierung 1990 bis 2016 auf der Grundlage der Kartierungsergebnisse zahlreicher ehrenamtlicher Mitarbeiter.* Druck und Verlag Steinmeier GmbH & Co. KG.

Agapova ND, Archarova KB, Vachtina LI, Zemskova EA, Travis LV. 1990. Chisla khromosom tsvetkovykh rastenii flory SSSR: Aceraceae – Menyanthaceae. Nauka, Saint Petersburg.

Andreev N. 1981. [Chromosome number reports] in Löve Á. (ed) Chromosome number reports LXX. *Taxon* **30**, 74–75 (1981)

Baksay L. 1958. The chromosome numbers of Ponto-Mediterranean plant species. *Ann. Hist. Nat. Mus. Natl. Hung*. **50**: 121–125.

Bancheva S, Greilhuber J. 2006. Genome size in Bulgarian Centaurea s.l. (Asteraceae). *Plant Systematics and Evolution* **257**: 95–117.

Barina Z, Somogyi G, Pifkó D, Rakaj M. 2018. Checklist of vascular plants of Albania. *Phytotaxa* **378**: 1–339.

Becker G. 2005. *Centaurea stoebe* subsp. *stoebe* in der südöstlichen Pfungstädter Gemarkung. *Hessische Floristische Briefe* **54**: 64–67.

Bohn & Gollub. 2006. The use and application of the map of the natural vegetation of Europe with particular reference to Germany. *Biology and Environment* **106**, 199-213.

Bónis É. 1969. *Die spätkeltische Siedlung Gellérthegy-Tabán in Budapest*. Budapest: Akadémiai Kiadó.

Brandza D, Ştefănescu S. 1898. *Flora Dobrogei*. C. Göbl.

Brennenstuhl G. 2020. Zum Verhalten von mit Grassaat ausgebreiteten gebietsfremden Sippen entlang der Bahnstrecke Stendal-Uelzen. *Mitteilungen zur floristischen Kartierung in Sachsen-Anhalt* **25**.

Broennimann O, Fitzpatrick MC, Pearman PB, Petitpierre B, Pellissier L, Yoccoz NG, Thuiller W, Fortin M, Randin C, Zimmermann NE, et al. 2012. Measuring ecological niche overlap from occurrence and spatial environmental data. *Global Ecology and Biogeography* **21**: 481–497.

Broennimann O, Guisan A. 2008. Predicting current and future biological invasions: both native and invaded ranges matter. *Biology Letters* **4**: 585–589.

Broennimann O, Mráz P, Petitpierre B, Guisan A, Müller-Schärer H. 2014. Contrasting spatio-temporal climatic niche dynamics during the eastern and western invasions of spotted knapweed in North America. *Journal of Biogeography* **41**: 1126–1136.

Broennimann O, Treier UA, Müller-Schärer H, Thuiller W, Peterson AT, Guisan A. 2007. Evidence of climatic niche shift during biological invasion. *Ecology Letters* **10**: 701–709.

Broz AK, Manter DK, Bowman G, Müller-Schärer H, Vivanco JM. 2009. Plant origin and ploidy influence gene expression and life cycle characteristics in an invasive weed. *BMC Plant Biology* **9**: 33.

Callaway RM, Waller LP, Diaconu A, Pal R, Collins AR, Mueller-Schaerer H, Maron JL. 2011. Escape from competition: Neighbors reduce Centaurea stoebe performance at home but not away. *Ecology* **92**: 2208–2213.

Carev I, Pustahija F, Ruščić M, Siljak-Yakovlev S. 2017. Chromosome number and ploidy level in seven *Centaurea species* from Croatia. *Flora* **27**, 289–294

Chytrý K, Willner W, Chytrý M, Divíšek J, Dullinger S. 2022. Central European forest–steppe: An ecosystem shaped by climate, topography and disturbances. *Journal of Biogeography* **49**: 1006–1020.

Clement EJ. 2002. More about *Centaurea rhenana* (and allies) in MW Yorks. *BSBI News* **89**: 33–34.

Collins AR, Müller-Schärer H. 2012. Influence of plant phenostage and ploidy level on oviposition and feeding of two specialist herbivores of spotted knapweed, *Centaurea stoebe*. *Biological Control* **60**: 148–153.

Collins AR, Naderi R, Mueller-Schaerer H. 2011. Competition between cytotypes changes across a longitudinal gradient in *Centaurea stoebe* (Asteraceae). *American Journal of Botany* **98**: 1935–1942.

Collins AR, Thalmann D, Müller-Schärer H. 2013. Cytotypes of *Centaurea stoebe* found to differ in root growth using growth pouches. *Weed Research* **53**: 159–163.

Czajlik Z, Tankó K, Timár L, Holl B. 2015. Remains of Celtic Settlement at Ráckeresztúr. In: Borhy L, Tankó K, Dévai K, eds. *Studia Archaeologica Nicolae Szabó LXXV Annos Nato dedicate*. Budapest: L'Harmattan, 77–94.

Damboldt J, Matthäs U. 1975. Chromosomenzahlen einiger mediterraner und mitteleuropäischer Centaurea-Arten (Asteraceae). *Plant Systematics and Evolution* **123**: 107–115.

D'Andrea L, Broennimann O, Kozlowski G, Guisan A, Morin X, Keller-Senften J, Felber F. 2009. Climate change, anthropogenic disturbance and the northward range expansion of *Lactuca serriola* (Asteraceae). *Journal of Biogeography* **36**: 1573–1587.

Divíšek J, Večeřa M, Welk E, Danihelka J, Chytrý K, Douda J, Chytrý M. 2022. Origin of the central European steppe flora: insights from palaeodistribution modelling and migration simulations. *Ecography* **2022**: e06293.

Dobrochayeva DM. 1965. *C. sarandinakiae* Illar. – Voloshka Sarandinaki. In: Visjulina OD (Ed.). *Flora of UkrSSR*. Kyiv: Naukova Dumka, 158–160.

Edgar RC. 2016. UNOISE2: improved error-correction for Illumina 16S and ITS amplicon sequencing. *BioRxiv* 081257.

Ellenberg H, Weber HE, Düll R, Wirth V, Werner W, Paulissen D. 1992. Zeigerwerte von Pflanzen in Mitteleuropa. *Scripta Geobotanica* **18**: 1–248.

Essl F, Dullinger S, Genovesi P, Hulme PE, Jeschke JM, Katsanevakis S, Kühn I, Lenzner B, Pauchard A, Pyšek P, et al. 2019. A conceptual framework for range-expanding species that track human-induced environmental change. *BioScience* **69**: 908–919.

Feurdean A, Grindean R, Florescu G, Tanţău I, Niedermeyer EM, Diaconu A-C, Hutchinson SM, Nielsen AB, Sava T, Panait A, et al. 2021. The transformation of the forest steppe in the lower Danube Plain of southeastern Europe: 6000 years of vegetation and land use dynamics. *Biogeosciences* **18**: 1081–1103.

Gastner MT, Seguy V, More P. 2018. Fast flow-based algorithm for creating density-equalizing map projections. *Proceedings of the National Academy of Sciences* **115**: E2156–E2164.

Geisen S, Kostenko O, Cnossen MC, Hooven FC ten, Vreš B, Putten WH van der. 2017. Seed and root endophytic fungi in a range expanding and a related plant species. *Frontiers in Microbiology* **8**: 1645.

Geisen S, Snoek LB, Ten Hooven FC, Duyts H, Kostenko O, Bloem J, Martens H, Quist CW, Helder JA, Van Der Putten WH. **2018**. Integrating quantitative morphological and qualitative molecular methods to analyse soil nematode community responses to plant range expansion (S Kembel, Ed.). *Methods in Ecology and Evolution* **9**: 1366–1378.

Georgiadis T. **1983**. Contribution à l´étude cytogéographique du genre Centaurea L. (section Acrolophus (Cass.) DC.) en Grèce. *Candollea* **38**: 3.

Gfeller V, Huber M, Förster C, Huang W, Köllner TG, Erb M. **2019**. Root volatiles in plant-plant interactions I: High root sesquiterpene release is associated with increased germination and growth of plant neighbours. *Plant, Cell & Environment* **42**: 1950–1963.

Gochu DI. **1973**. Nekotorye dannye o khromosomnykh chislakh vidov Centaurea L., proizrastayushchikh v Moldavii. *Bot Zhurn* **58**: 77–78.

Gregor T. **2001**. Fundmeldung [*Centaurea stoebe* subsp. *micrantha*]. *Botanische Vereinigung fuer Naturschutz Hessen* **13**: 78–79.

Gregor T, Hand R. **2008**. Chromosomenzahlen von Farn- und Samenpflanzen aus Deutschland. *Kochia* **3**, 55–60.

Guinochet M. **1957**. Contribution à l´étude caryologique du genre Centaurea L. sens. lat. *Bulletin de la Société d'historie naturelle d'Afrique du Nord* **48**: 282–300.

Gutte P, Fischer J. **2012**. Floristische Neufunde aus Nordwest-Saxony. *Sächsische floristische Mittteilungen (Leipzig)* **15**: 45–50.

Hahn MA, Buckley YM, Müller-Schärer H. **2012a**. Increased population growth rate in invasive polyploid *Centaurea stoebe* in a common garden. *Ecology Letters* **15**: 947–954.

Hahn MA, van Kleunen M, Müller-Schärer H. **2012b**. Increased phenotypic plasticity to climate may have boosted the invasion success of polyploid *Centaurea stoebe. PloS One* **7**: e50284.

Hahn MA, Lanz T, Fasel D, Müller-Schärer H. **2013**. Increased seed survival and seedling emergence in a polyploid plant invader. *American Journal of Botany* **100**: 1555–1561.

Hahn MA, Müller-Schärer H. **2013**. Cytotype differences modulate eco-geographical differentiation in the widespread plant *Centaurea stoebe*. *Ecology* **94**: 1005–1014.

He W-M, Feng Y, Ridenour WM, Thelen GC, Pollock JL, Diaconu A, Callaway RM. **2009**. Novel weapons and invasion: biogeographic differences in the competitive effects of *Centaurea maculosa* and its root exudate (±)-catechin. *Oecologia* **159**: 803–815.

He W-M, Thelen GC, Ridenour WM, Callaway RM. **2010**. Is there a risk to living large? Large size correlates with reduced growth when stressed for knapweed populations. *Biological Invasions* **12**: 3591–3598.

Hejcman M, Hejcmanová P, Pavlů V, Beneš J. **2013**. Origin and history of grasslands in Central Europe – a review. *Grass and Forage Science* **68**: 345–363.

Henery ML, Bowman G, Mráz P, Treier UA, Gex-Fabry E, Schaffner U, Müller-Schärer H. **2010**. Evidence for a combination of pre-adapted traits and rapid adaptive change in the invasive plant *Centaurea stoebe*. *Journal of Ecology* **98**: 800–813.

Hufbauer RA, Sforza R. **2008**. Multiple Introductions of Two Invasive Centaurea Taxa Inferred from cpDNA Haplotypes. *Diversity and Distributions* **14**: 252–261.

Illyés E. **2009.** Classification of semi-dry grassland vegetation in Hungary. *Preslia* **81**, 239–260.

Jamrichová E, Petr L, Jiménez-Alfaro B, Jankovská V, Dudová L, Pokorný P, Kołaczek P, Zernitskaya V, Čierniková M, Břízová E, et al. 2017. Pollen-inferred millennial changes in landscape patterns at a major biogeographical interface within Europe. *Journal of Biogeography* **44**: 2386–2397.

John H, Stolle J. **2006**. Wandlung der Flora durch Eingriffe des Menschen, dargestellt anhand aktueller Funde höherer Pflanzen in der Umgebung von Halle (Saale). *Mitteilungen zur floristischen Kartierung in Sachsen-Anhalt* **11**.

John H, Stolle J. **2007**. Aktuelle Nachweise von Samenpflanzen in der Umgebung von Halle (Saale). *Mitteilungen zur floristischen Kartierung in Sachsen-Anhalt* **12**: 87–94.

Kajtoch Ł, Cieślak E, Varga Z, Paul W, Mazur MA, Sramkó G, Kubisz D. **2016**. Phylogeographic patterns of steppe species in Eastern Central Europe: a review and the implications for conservation. *Biodiversity and Conservation* **25**: 2309–2339.

Kaplan JO, Krumhardt KM, Zimmermann N. **2009**. The prehistoric and preindustrial deforestation of Europe. *Quaternary Science Reviews* **28**: 3016–3034.

Katoh K, Toh H. **2008**. Recent developments in the MAFFT multiple sequence alignment program. *Briefings in Bioinformatics* **9**: 286–298.

Kiehn M**, Vitek E, Dobeš C**. **2000**. [Chromosome number reports].” In: Dobeš C, Vitek E (eds.) *Documented chromosome number checklist of Austrian vascular plants.* Verlag des Naturhistorischen Museums Wien, Vienna.

Kiehn M, Vitek E, Hellmayr E, Walter J, Tschenett J, Justin C, Mann M. **1991**. Beitraege zur Flora von Oesterreich: Chromosomenzaehlungen. *Verhandlungen der Zoologisch-Botanischen Gesellschaft in Oesterreich*. Wien **128**: 19–39.

Kirschner P, Záveská E, Gamisch A, Hilpold A, Trucchi E, Paun O, Sanmartín I, Schlick-Steiner BC, Frajman B, Arthofer W, et al. 2020. Long-term isolation of European steppe outposts boosts the biome’s conservation value. *Nature Communications* **11**: 1968.

Klotz J. **2015**. *Centaurea australis* als übersehene, jungneophytische Ruderalpflanze in Regensburger Bahnanlagen. *Hoppea - Denkschriften der Regensburgischen Botanischen Gesellschaft* **76**: 142–146.

Klug W. **2007**. Ausbreitung und pflanzensoziologische Amplitude des Neophyten *Centaurea stoebe* subsp. *micranthos* auf dem Seeberg bei Gotha. *Informationen zur floristischen Kartierung in Thüringen* **26**: 17–24.

Koorem K, Kostenko O, Snoek LB, Weser C, Ramirez KS, Wilschut RA, van der Putten WH. **2018**. Relatedness with plant species in native community influences ecological consequences of range expansions. *Oikos* **127**: 981–990.

Koorem K, Snoek BL, Bloem J, Geisen S, Kostenko O, Manrubia M, Ramirez KS, Weser C, Wilschut RA, van der Putten WH. **2020**. Community-level interactions between plants and soil biota during range expansion. *The Journal of Ecology* **108**: 1860–1873.

Koorem K, Wilschut RA, Weser C, Van Der Putten WH. **2021**. Disentangling nematode and arbuscular mycorrhizal fungal community effect on the growth of range-expanding *Centaurea stoebe* in original and new range soil. *Plant and Soil* **466**: 207–221.

Korneck. **2004**. *Centaurea stoebe* subsp. *micranthos*, Kleinköpfige Flockenblume, eine verkannte Sippe unserer Flora (vorläufige Mitteilung). *Hessische floristische Briefe* **51**, 1–5.

Korneck D. **2006**. *Centaurea stoebe* subsp. *stoebe* und subsp. *australis* in Südhessen. *Hessische floristische Briefe* **55**, 21–30.

Korneck D. 2016. Pflanzengesellschaften mit *Centaurea stoebe* s.l. im Rheingebiet. *Mittteilungen des badsichen Labdesvereins für Naturkunde* **22**, 23–58.

Kožić K, Hartmann M, Callaway RM, Hensen I, Nagy DU, Mráz P, Al-Gharaibeh MM, Bancheva S, Diaconu A, Danihelka J *et al.* 2024. Performance in the recruitment life stage and its potential contribution to invasive success in the polyploid invader *Centaurea stoebe. Neobiota.* DOI: 10.3897/neobiota.@@.127654

Kuneš P, Svobodová-Svitavská H, Kolář J, Hajnalová M, Abraham V, Macek M, Tkáč P, Szabó P. 2015. The origin of grasslands in the temperate forest zone of east-central Europe: long-term legacy of climate and human impact. *Quaternary Science Reviews* **116**: 15–27.

Kuzmanov B, Georgieva S. 1976. [Chromosome number reports]. In: Löve Á (ed) *IOPB chromosome number reports LIII. Taxon* **25**, 500.

Kuzmanov B, Georgieva S. 1977. [Chromosome number reports]. In: Löve Á (ed) *IOPB chromosome number reports LVII. Taxon* **26**, 443–452.

Kuzmanov B, Georgieva SB, Nikolova VA. 1986. Chromosome numbers of Bulgarian flowering plants. I. Fam. Asteraceae. *Fitologija (Sofia)* **31**: 71–74.

Kuzmanov B, Ninova D, Georgieva S. 1981. [Chromosome number reports]. In: Löve Á (ed) *IOPB chromosome number reports LXXII. Taxon* **30**, 702.

Landeck I, Eiser C, Ludwig I, Thümmel G. 2013. Zur aktuellen Verbreitung der Europäischen Gottesanbeterin, *Mantis religiosa* LINNAEUS, 1758 (Mantodea, Mantidae), im Land Brandenburg. *Märkische Entomologische Nachrichten*, **15**: 227–248.

Lang PLM, Willems FM, Scheepens JF, Burbano HA, Bossdorf O. 2019. Using herbaria to study global environmental change. *The New Phytologist* **221**: 110–122.

Li K, Veen GF, ten Hooven FC, Harvey JA, van der Putten WH. 2023. Soil legacy effects of plants and drought on aboveground insects in native and range-expanding plant communities. Ecology Letters **26**: 37–52.

Löve A, Löve D. 1982. [Chromosome number reports]. In: Löve Á (ed) *IOPB chromosome number reports LXXVI. Taxon* **31**, 583–587.

Lövkvist B, Hultgård U-M. 1999. Chromosome numbers in south Swedish vascular plants. *Opera Botanica* **137**: 1–42.

Lovrić AZ. 1982. [Chromosome number reports]. In: Löve Á (ed) *IOPB chromosome number reports LXXVII. Taxon* **31**, 762–763.

Lustenhouwer N, Parker IM. 2022. Beyond tracking climate: Niche shifts during native range expansion and their implications for novel invasions. *Journal of Biogeography* **49**: 1481–1493.

Maddox DM. 1979. The Knapweeds: Their Economics and Biological Control in the Western States, U.S.A. *Rangelands* **1,** 139–141.

Malten A, Zizka G, Dietz M, Fehlow M, Grenz M, Schmitt P, Simon O, Zub P. 2005. *Erfassung von Flora, Fauna und Vegetation auf dem Flughafen Frankfurt am Main.* Frankfurt: Senckenberg-Museum.

Maron JL, Klironomos J, Waller L, Callaway RM. 2014. Invasive plants escape from suppressive soil biota at regional scales. *Journal of Ecology* **102**: 19–27.

Maron JL, Waller LP, Hahn MA, Diaconu A, Pal RW, Müller-Schärer H, Klironomos JN, Callaway RM. 2013. Effects of soil fungi, disturbance and propagule pressure on exotic plant recruitment and establishment at home and abroad. *Journal of Ecology* **101**: 924–932.

Marrs RA, Sforza R, Hufbauer RA. 2008. Evidence for multiple introductions of *Centaurea stoebe micranthos* (spotted knapweed, asteraceae) to North America. *Molecular Ecology* **17**: 4197–4208.

Martin M. 2011. Cutadapt removes adapter sequences from high-throughput sequencing reads. *EMBnet.journal* **17**: 10.

Mártonfiová L, Mikoláš V, Mártonfi P. 2018. [Chromosome number reports]. In: Marhold K, Kučera J (eds) *IAPT chromosome data 27*. *Taxon* **67**: 1041–1047.

Meierott L. 2008. *Neue Flora von Schweinfurt Bd. 2*. Eching: IHW-Verlag.

Molnár Z, Biró M, Bölöni J, Horváth F. 2009. Distribution of the (semi-)natural habitats in Hungary I. Marshes and grasslands. *Acta Botanica Hungarica* **50**: 59–105.

Morais P, Reichard M. 2018. Cryptic invasions: A review. *Science of The Total Environment* **613–614**: 1438–1448.

Moysiyenko II, Sudnik-Wójcikowska B. 2010. Flora of kurgans in the forest steppe zone in Ukraine. *Chornomorsky Botanical Journal* **6**: 162–199.

Mráz P, Bourchier RS, Treier UA, Schaffner U, Müller-Schärer H. 2011. Polyploidy in Phenotypic Space and Invasion Context: A Morphometric Study of *Centaurea stoebe* s.l. *International Journal of Plant Sciences* **172**: 386–402.

Mráz P, Garcia-Jacas N, Gex-Fabry E, Susanna A, Barres L, Müller-Schärer H. 2012a. Allopolyploid origin of highly invasive *Centaurea stoebe* s.l. (Asteraceae). *Molecular Phylogenetics and Evolution* **62**: 612–623.

Mráz P, Španiel S, Keller A, Bowmann G, Farkas A, Šingliarová B, Rohr RP, Broennimann O, Müller-Schärer H. 2012b. Anthropogenic disturbance as a driver of microspatial and microhabitat segregation of cytotypes of *Centaurea stoebe* and cytotype interactions in secondary contact zones. *Annals of Botany* **110**: 615–627.

Mráz P, Španiel S, Skokanová K, Šingliarová B. 2022. Temporal stability of spatial cytotype structure in mixed-ploidy populations of *Centaurea stoebe. AoB PLANTS* **14**: plac052.

Mráz P, Tarbush E, Müller-Schärer H. 2014. Drought tolerance and plasticity in the invasive knapweed *Centaurea stoebe* s.l. (Asteraceae): effect of populations stronger than those of cytotype and range. *Annals of Botany* **114**: 289–299.

Nagel J. 2000. [Chromosome number reports]. In: Ochsmann J (ed) *Morphologische und molekularsystematische Untersuchungen an der Centaurea stoebe L. Gruppe (Asteraceae-Cardueae) in Europe. Dissertationes Botanicae* **324**: 233–234.

Novikov A. 2021. Developing the GIS-based maps of the geomorphological and phytogeographical division of the Ukrainian Carpathians for routine use in biogeography. *Biogeographia – The Journal of Integrative Biogeography* **36**: a009.

Ochsmann J. 1999. Chromosomenzahlen einiger europäischer Centaurea-Sippen. *Haussknechtia* **7**: 59–65.

Ochsmann J. 2000. *Morphologische und molekularsystematische Untersuchungen an der Centaurea stoebe L.-Gruppe (Asteraceae-Cardueae) in Europa*. Berlin Stuttgart: Cramer in der Gebr.-Borntraeger-Verl.-Buchh.

Olson DM, Dinerstein E, Wikramanayake, ED, Burgess, ND, Powell, GV, Underwood, EC, D'amico JA, Itoua I, Strand HE, Morrison JC, Loucks CJ, Allnutt TF, Ricketts TH, Kura Y, Lamoreux JF, Wettengel WW, Hedao P, Kassem, KR. 2001. Terrestrial Ecoregions of the World: A New Map of Life on Earth: A new global map of terrestrial ecoregions provides an innovative tool for conserving biodiversity. *BioScience*, **51**, 933-938.

Otisková V, Koutecký T, Kolář F, Koutecký P. 2014. Occurrence and habitat preferences of diploid and tetraploid cytotypes of *Centaurea stoebe* in the Czech Republic. *Preslia* **86,** 67-80.

Petitpierre B, Broennimann O, Kueffer C, Daehler C, Guisan A. 2017. Selecting predictors to maximize the transferability of species distribution models: lessons from cross-continental plant invasions. *Global Ecology and Biogeography* **26**: 275–287.

Petitpierre B, Kueffer C, Broennimann O, Randin C, Daehler C, Guisan A. 2012. Climatic Niche Shifts Are Rare Among Terrestrial Plant Invaders. *Science* **335**: 1344–1348.

Pokorný P, Chytrý M, Juřičková L, Sádlo J, Novák J, Ložek V. 2015. Mid-Holocene bottleneck for central European dry grasslands: Did steppe survive the forest optimum in northern Bohemia, Czech Republic? *The Holocene* **25**: 716–726.

Probatova NS, Motorykina TN, Rudyka EG, Kriukova MV, Nechaev VA. 2013. [Chromosome number reports]. In: Marhold K. (ed) *IAPT/IOPB chromosome data 15. Taxon* **62**: 1073–1083.

Pustahija F, Brown S, Bogunić F, Bašić N, Muratović E, Ollier S, Hidalgo O, Bourge M, Stevanović V, Siljak-Yakovlev S. 2013. Small genomes dominate in plants growing on serpentine soils in West Balkans, an exhaustive study of 8 habitats covering 308 taxa. *Plant and Soil* **373**.

Ramirez KS, Snoek LB, Koorem K, Geisen S, Bloem LJ, Ten Hooven F, Kostenko O, Krigas N, Manrubia M, Caković D, et al. 2019. Range-expansion effects on the belowground plant microbiome. *Nature Ecology & Evolution* **3**: 604–611.

Rechinger KH. 1950. Notizen zur Adventiv- und Ruderalflora von Wien. *Österreichische botanische Zeitschrift* **97**: 114–123.

Ridenour WM, Vivanco JM, Feng Y, Horiuchi J, Callaway RM. 2008. No Evidence for Trade-Offs: Centaurea Plants from America Are Better Competitors and Defenders. *Ecological Monographs* **78**: 369–386.

Romahn K. 2021. *Rote Listen der in Schleswig-Holstein gefährdeten Pflanzen und Tiere: Die Farn- und Blütenpflanzen Schleswig-Holsteins.* Verlag: Landesamt für Landwirtschaft, Umwelt und ländliche Räume des Landes Schleswig-Holstein).

Rosche C, Baasch A, Runge K, Brade P, Träger S, Parisod C, Hensen I. 2022. Tracking population genetic signatures of local extinction with herbarium specimens. *Annals of Botany* **129**: 857–868.

Rosche C, Durka W, Hensen I, Mráz P, Hartmann M, Müller-Schärer H, Lachmuth S. 2016. The population genetics of the fundamental cytotype-shift in invasive *Centaurea stoebe* s.l.: genetic diversity, genetic differentiation and small-scale genetic structure differ between cytotypes but not between ranges. *Biological Invasions* **18**: 1895–1910.

Rosche C, Hensen I, Lachmuth S. 2018a. Local pre-adaptation to disturbance and inbreeding-environment interactions affect colonisation abilities of diploid and tetraploid *Centaurea stoebe*. *Plant Biology* (Stuttgart, Germany) **20**: 75–84.

Rosche C, Hensen I, Mráz P, Durka W, Hartmann M, Lachmuth S. 2017. Invasion success in polyploids: the role of inbreeding in the contrasting colonization abilities of diploid versus tetraploid populations of *Centaurea stoebe* s.l. *Journal of Ecology* **105**: 425–435.

Rosche C, Schrieber K, Lachmuth S, Durka W, Hirsch H, Wagner V, Schleuning M, Hensen I. 2018b. Sex ratio rather than population size affects genetic diversity in *Antennaria dioica*. *Plant Biology* **20**: 789–796.

Ryff LE. 2009. Voloshka Sarandinaki – *Centaurea sarandinakiae* N.B.Illar. (*C. transcaucasica auct.* non Grossh.). In: Didukh YP (ed.), *Red Book of Ukraine*. Kyiv: Globalconsulting, 311.

Schaffner U, Ridenour WM, Wolf VC, Bassett T, Müller C, Müller-Schärer H, Sutherland S, Lortie CJ, Callaway RM. **2011**. Plant invasions, generalist herbivores, and novel defense weapons. *Ecology* **92**: 829–835.

Schneider T, Caspari S, Schneider C, Weicherding F-J. 2020. *Rote Liste und Gesamtartenliste der Gefäßpflanzen (Tracheophyta) des Saarlandes – 4. Fassung.* Saarbrücken and Landsweiler-Reden: Ministerium für Umwelt und Verbraucherschutz and DELATTINIA. 1-105.

Schönswetter P, Tribsch A. **2005**. Vicariance and dispersal in the alpine perennial *Bupleurum stellatum* L. (Apiaceae). *Taxon* **54**: 725–732.

Semenova I, Slizhe M. **2020**. Synoptic Conditions of Droughts and Dry Winds in the Black Sea Steppe Province Under Recent Decades. *Frontiers in Earth Science* **8**: 69.

Shipunov A, Newcombe G, Raghavendra AKH, Anderson CL. **2008**. Hidden diversity of endophytic fungi in an invasive plant. *American Journal of Botany* **95**: 1096–1108.

Shynder O. **2021**. The taxonomic complex *Centaurea stoebe* s. l. (Asteraceae) in the flora of Ukraine. *Novitates Theriologicae* **12**: 237–251.

Skalińska M, Czapik R, Piotrowicz M. **1959**. Further studies in chromosome numbers of Polish Angiosperms (Dicotyledons). *Acta Societatis Botanicorum Poloniae* **28**: 487–529.

Smarda P, Knápek O, Šilerová A, Horová L, Grulich V, Danihelka J, Veselý P, Smerda J, Rotreklová O, Bures P, et al. 2019. Genome sizes and genomic guanine+cytosine (GC) contents of the Czech vascular flora with new estimates for 1700 species. *Preslia* **91**: 117–142.

Somodi I, Molnár Z, Czúcz B, Bede-Fazekas Á, Bölöni J, Pásztor L, Laborczi A, Zimmermann NE. **2017**. Implementation and application of multiple potential natural vegetation models – a case study of Hungary (I Kühn, Ed.). *Journal of Vegetation Science* **28**: 1260–1269.

Španiel S, Marhold K, Hodálová I, Zozomova-Lihova J. **2008**. Diploid and Tetraploid Cytotypes of *Centaurea stoebe* (Asteraceae) in Central Europe: Morphological Differentiation and Cytotype Distribution Patterns. *Folia Geobotanica* **43**: 131–158.

Ssymank A. **2013**. Die Steppenlebensräume im Natura 2000-Netzwerk der EU 27-Staaten. In: Baumbach H, Pfützenreuter S (eds) *Steppenlebensräume Europas: Gefährdung, Erhaltungsmaßnahmen und Schutz*. Harxheim: ConchBooks, 13–24.

Sudnik-Wójcikowska B, Moysiyenko II, Zachwatowicz M, Jabłońska E. **2011**. The value and need for protection of kurgan flora in the anthropogenic landscape of steppe zone in Ukraine. *Plant Biosystems - An International Journal Dealing with all Aspects of Plant Biology* **145**: 638–653.

Sun Y, Collins AR, Schaffner U, Müller-Schärer H. **2013**. Dissecting impact of plant invaders: do invaders behave differently in the new range? *Ecology* **94**: 2124–2130.

Sun Y, Müller-Schärer H, Maron JL, Schaffner U. **2015**. Biogeographic effects on early establishment of an invasive alien plant. *American Journal of Botany* **102**: 621–625.

Sun Y, Müller-Schärer H, Schaffner U. **2014**. Plant neighbours rather than soil biota determine impact of an alien plant invader. *Functional Ecology* **28**: 1545–1555.

Sun Y, Müller-Schärer H, Schaffner U. **2016**. Neighbour Origin and Ploidy Level Drive Impact of an Alien Invasive Plant Species in a Competitive Environment. *PloS One* **11**: e0155712.

Stoyanov S, Apostolova-Stoyanova N. 2024. Checklist of the flora in the Rusenski Lom River Valley (Northeast Bulgaria). *Phytologia Balcanica* **30**:45–72.

Tamis WLM. **2005**. Changes in the flora of the Netherlands in the 20th century. *Gorteria Supplement* **6**: 1–233.

Taylor J. **2000**. *Centaurea rhenana* on a Yorkshire motorway. *BSBI News* **85**: 45–46.

Thakur MP, van der Putten WH, Apon F, Angelini E, Vreš B, Geisen S. **2021**. Resilience of rhizosphere microbial predators and their prey communities after an extreme heat event. *Functional Ecology* **35**: 216–225.

Thébault A, Frey B, Mitchell EAD, Buttler A. **2010**. Species-specific effects of polyploidisation and plant traits of *Centaurea maculosa* and *Senecio inaequidens* on rhizosphere microorganisms. *Oecologia* **163**: 1011–1020.

Thébault A, Gillet F, Müller-Schärer H, Buttler A. **2011**. Polyploidy and invasion success: trait trade-offs in native and introduced cytotypes of two Asteraceae species. *Plant Ecology* **212**: 315–325.

Thébault A, Stoll P, Buttler A. **2012**. Complex interactions between spatial pattern of resident species and invasiveness of newly arriving species affect invasibility. *Oecologia* **170**: 1133–1142.

Thiers B. **2023**. Index Herbariorum: A global directory of public herbaria and associated staff. New York Botanical Garden's Virtual Herbarium. Available at: http://sweetgum.nybg.org/ih.

Treier UA, Broennimann O, Normand S, Guisan A, Schaffner U, Steinger T, Müller-Schärer H. **2009**. Shift in cytotype frequency and niche space in the invasive plant *Centaurea maculosa*. *Ecology* **90**: 1366–1377.

Tupu E, Chifu T. **2021**. *Flora şi vegetaţia Dealurilor Tulcei: Dobrogea de Nord*. Iaşi: Editura Universităţii "Al.I. Cuza.

Tyler T. **1999**. Några sentida skånska invandrares historia. *Lunds Botaniska Förening Medlemsblad* **4**: 14–30.

Van Loon JC, De Jong H. **1978**. [Chromosome numbers]. In: Löve Á (ed) *IOPB chromosome number reports LXXVI*. *Taxon* **31**: 589–592.

Van Loon JC, Van Setten AK. **1982**. [Chromosome numbers]. In: Löve Á (ed) *IOPB chromosome number reports LIX*. *Taxon* **27**: 56-60.

Vassilev K, Apostolova I. **2013**. Bulgarian steppic veg-etation: an overview. In: Baumbach H, Pfützenreuter S (eds) *Steppenlebensräume Europas: Gefährdung, Erhaltungsmaßnahmen und Schutz*. Harxheim: ConchBooks, 191–200.

Venables WN, Ripley BD. **2002**. *Modern applied statistics with S.* New York: Springer.

Vollmann F. 1914. *Flora von Bayern*. Stuttgart: E. Ulmer.

Welss W, Reger P, Nezadal W. **2008**. Zur Verbreitung von *Centaurea stoebe* L. subsp. *stoebe* und *Centaurea stoebe* subsp. *australis* (A. Kern.) Greuter (Asteraceae im Nürnberger Becken. *RegnitzFlora - Mittelungen des Vereins zur Erforschung der Flora des Regnitzgebietes* **2**: 44–53.

Wilschut RA, Geisen S, Martens H, Kostenko O, de Hollander M, Ten Hooven FC, Weser C, Snoek LB, Bloem J, Caković D, et al. 2019. Latitudinal variation in soil nematode communities under climate warming-related range-expanding and native plants. *Global Change Biology* **25**: 2714–2726.

Wilschut RA, Kostenko O, Koorem K, van der Putten WH. **2018**. Nematode community responses to range-expanding and native plant communities in original and new range soils. *Ecology and Evolution* **8**: 10288–10297.

Wilschut RA, Magnée KJH, Geisen S, van der Putten WH, Kostenko O. **2020**. Plant population and soil origin effects on rhizosphere nematode community composition of a range-expanding plant species and a native congener. *Oecologia* **194**: 237–250.

Wit SW, Schwabe A. 2010. The fate of sheep-dispersed seeds: Plant species emergence and spatial patterns. *Flora - Morphology, Distribution, Functional Ecology of Plants* **205**: 656–665.

Wood SN, Pya N, Säfken B. 2016. Smoothing parameter and model selection for general smooth models. *Journal of the American Statistical Association* **111**: 1548–1563.

Záborský J. 1970. [Reports in Cyanus]. In: Májovský J *et al.* (eds) *Index of chromosome numbers of Slovakian flora (Part 1). Acta Facultatis Rerum Naturalium Universitatis Comenianae, Botanica* **16**: 9.

Zalasiewicz J, Waters CN, Williams M, Barnosky AD, Cearreta A, Crutzen P, Ellis E, Ellis MA, Fairchild IJ, Grinevald J, et al. 2015. When did the Anthropocene begin? A mid-twentieth century boundary level is stratigraphically optimal. *Quaternary International* **383**: 196–203.

Zhang J, Kobert K, Flouri T, Stamatakis A. 2014. PEAR: a fast and accurate Illumina Paired-End reAd mergeR. *Bioinformatics* (Oxford, England) **30**: 614–620.

Zündorf H-J, Günther K-F, Korsch H, Westhus W. 2006. *Flora von Thüringen: die wildwachsenden Farn- und Blütenpflanzen Thüringens.* Jena: Weissdorn-Verlag.
